# Description and evaluation of the Community Multiscale Air Quality (CMAQ) modeling system version 5.1

**DOI:** 10.5194/gmd-10-1703-2017

**Published:** 2017

**Authors:** K. Wyat Appel, Sergey L. Napelenok, Kristen M. Foley, Havala O. T. Pye, Christian Hogrefe, Deborah J. Luecken, Jesse O. Bash, Shawn J. Roselle, Jonathan E. Pleim, Hosein Foroutan, William T. Hutzell, George A. Pouliot, Golam Sarwar, Kathleen M. Fahey, Brett Gantt, Robert C. Gilliam, Nicholas K. Heath, Daiwen Kang, Rohit Mathur, Donna B. Schwede, Tanya L. Spero, David C. Wong, Jeffrey O. Young

**Affiliations:** 1 Computational Exposure Division, National Exposure Research Laboratory, Office of Research and Development, US Environmental Protection Agency, Research Triangle Park, NC, USA; 2 Air Quality Analysis Division, Office of Air Quality Planning and Standards, Office of Air and Radiation, US Environmental Protection Agency, Research Triangle Park, NC, USA; 3 Systems Exposure Division, National Exposure Research Laboratory, Office of Research and Development, US Environmental Protection Agency, Research Triangle Park, NC, USA

## Abstract

The Community Multiscale Air Quality (CMAQ) model is a comprehensive multipollutant air quality modeling system developed and maintained by the US Environmental Protection Agency’s (EPA) Office of Research and Development (ORD). Recently, version 5.1 of the CMAQ model (v5.1) was released to the public, incorporating a large number of science updates and extended capabilities over the previous release version of the model (v5.0.2). These updates include the following: improvements in the meteorological calculations in both CMAQ and the Weather Research and Forecast (WRF) model used to provide meteorological fields to CMAQ, updates to the gas and aerosol chemistry, revisions to the calculations of clouds and photolysis, and improvements to the dry and wet deposition in the model. Sensitivity simulations isolating several of the major updates to the modeling system show that changes to the meteorological calculations result in enhanced afternoon and early evening mixing in the model, periods when the model historically underestimates mixing. This enhanced mixing results in higher ozone (O_3_) mixing ratios on average due to reduced NO titration, and lower fine particulate matter (PM_2.5_) concentrations due to greater dilution of primary pollutants (e.g., elemental and organic carbon). Updates to the clouds and photolysis calculations greatly improve consistency between the WRF and CMAQ models and result in generally higher O_3_ mixing ratios, primarily due to reduced cloudiness and attenuation of photolysis in the model. Updates to the aerosol chemistry result in higher secondary organic aerosol (SOA) concentrations in the summer, thereby reducing summertime PM_2.5_ bias (PM_2.5_ is typically underestimated by CMAQ in the summer), while updates to the gas chemistry result in slightly higher O_3_ and PM_2.5_ on average in January and July. Overall, the seasonal variation in simulated PM_2.5_ generally improves in CMAQv5.1 (when considering all model updates), as simulated PM_2.5_ concentrations decrease in the winter (when PM_2.5_ is generally overestimated by CMAQ) and increase in the summer (when PM_2.5_ is generally underestimated by CMAQ). Ozone mixing ratios are higher on average with v5.1 vs. v5.0.2, resulting in higher O_3_ mean bias, as O_3_ tends to be overestimated by CMAQ throughout most of the year (especially at locations where the observed O_3_ is low); however, O_3_ correlation is largely improved with v5.1. Sensitivity simulations for several hypothetical emission reduction scenarios show that v5.1 tends to be slightly more responsive to reductions in NO_*x*_ (NO + NO_2_), VOC and SO_*x*_ (SO_2_ + SO_4_) emissions than v5.0.2, representing an improvement as previous studies have shown CMAQ to underestimate the observed reduction in O_3_ due to large, widespread reductions in observed emissions.

## 1 Introduction

Numerous federal (e.g., United States Environmental Protection Agency, USEPA), state and private entities rely on numerical model simulations of atmospheric chemistry, transport and deposition of airborne emissions as well as the resulting pollutants as part of their decision-making process for air quality management and mitigation (e.g., Scheffe et al., 2007). Chemical transport models (CTMs), such as the Community Multiscale Air Quality (CMAQ) model (Byun and Schere, 2006), are often employed to provide information about the potential effects of emission control strategies (e.g., Fann et al., 2009) and climate change (e.g., Nolte et al., 2008), and to provide next-day air quality forecasts (e.g., Eder et al., 2006) in order to inform and protect the public from potentially harmful air pollutants. Since these models are often used to inform the standard setting and implementation for criteria pollutants (e.g., ozone (O_3_) and fine particulate matter (PM_2.5_)), they must be maintained at the state-of-the-science level. New versions of the CMAQ model have been released periodically over the past 15 years, with each new version consisting of numerous updates to the scientific algorithms within the model, while also improving the quality of the input data used. Collectively, these updates are aimed at improving the underlying science of atmospheric dynamics and chemistry represented in the model, extending the capabilities for emerging applications, and reducing systematic biases in the modeling system. Every new release of the CMAQ model undergoes extensive evaluation in order to establish its credibility (e.g., Mebust et al., 2003; Appel et al., 2007, 2008, 2013; Foley et al., 2010) and documents its performance relative to previous versions. Most recently, the CMAQ modeling system version 5.1 (v5.1) has been tested and evaluated against observations and was publicly released in December 2015 (http://www.cmaq-model.org/).

The scientific upgrades in the CMAQv5.1 modeling system include major revisions to the Pleim-Xiu land-surface model (PX-LSM; Pleim and Xiu, 1995) and the asymmetric convective mixing version 2 (ACM2; Pleim, 2007a, b) planetary boundary layer (PBL) model in the Weather Research And Forecast (WRF) model version 3.7 (Skamarock et al., 2008), which required revisions to the ACM2 scheme in CMAQ to maintain consistency. Corrections were also made to the Monin-Obukhov length (MOL) calculation in CMAQv5.1 to make it consistent with the calculation in the WRF model. The changes to the PX-LSM, ACM2 and MOL calculations in CMAQ had significant impact on the mixing within both WRF and CMAQ, and hence large impacts on the pollutant concentrations in CMAQ. These updates are described in Sect. 2.1. A new explicit treatment of secondary organic aerosol (SOA) formation from isoprene, alkenes and polycyclic aromatic hydrocarbons (PAHs) was also added in CMAQv5.1. Additionally, two aerosol mechanisms are now available in v5.1, AERO6 and AERO6i (with isoprene extensions), which include updates to the SOA and ISORROPIA algorithms (Nenes et al., 1998, 1999). The AERO5 mechanism has been deprecated and is no longer available. The updates to the aerosol treatment in v5.1 are described in Sect. 2.2. Significant changes were also made to the in-line calculation of photolysis rates (described in Sect. 2.3). The photochemistry in v5.1 underwent major changes; specifically, the photochemical cross sections and quantum yields for the carbon bond 2005 e51 (CB05e51) chemical mechanism were updated, along with updates to inorganic and organic chemical reaction rates and products to ensure consistency with the International Union of Pure and Applied Chemistry (IUPAC). Finally, the representation of organic nitrate species in CB05e51 was added. These updates are described in Sect. 2.4.

Section 2 provides a brief description of the major scientific and structural improvements included in v5.1. The model configuration and observational data sets used in the model evaluation are provided in Sect. 3. The evaluation of v5.1 is then presented in two parts. Section 4 documents the evaluation of several specific changes that were isolated as part of the overall testing of the model. Specifically, Sect. 4.1 evaluates the meteorological updates in WRF and CMAQ; Sect. 4.2 evaluates the aerosol updates; Sect. 4.3 evaluates the changes to the inline photolysis calculation and the representation of clouds within CMAQv5.1; and Sect. 4.4 evaluates the updates to the CB05e51 chemical mechanism. These increments were chosen as the focus of this paper because they represent a fundamental change from the previously released model version and had the propensity to impact model performance for criteria pollutants. The second portion of the evaluation, presented in Sect. 5, summarizes the overall change in PM_2.5_ and O_3_ model performance with v5.1 compared to the previously released version (CMAQ version 5.0.2: v5.0.2). Section 6 provides a discussion of the model response of O_3_ and PM_2.5_ to hypothetical reductions in emissions. Finally a summary discussion in provided in Sect. 7.

## 2 Review of scientific improvements in CMAQv5.1

Improvements to the v5.1 modeling system are the result of many years of scientific advancements derived from laboratory, field and numerical experiments as well as the efforts of a relatively small group of model developers that both investigate avenues for model improvements and then update the model (i.e., write code). Given the large community of CMAQ model users, there are never sufficient resources to diagnose and address every issue in the modeling system that has been reported. As such, it is necessary to prioritize updates to the model based on many different factors, including results from evaluations of past model versions, existing and upcoming regulatory needs, emerging scientific issues, requests from the CMAQ user community, and the expertise within the model developer group to meet those needs and requests. The updates presented herein represent the “major” updates made to the CMAQ modeling system from the previous model version, and therefore do not constitute a fully comprehensive description of all the changes made to the system. This section briefly describes these “major” updates to CMAQ, providing the reader with an understanding of what was updated in the model and why. A comprehensive description of all the updates made in v5.1 and indepth technical documentation of those changes can be found on the CMAS Center website for the CMAQv5.1 release at https://cmaswiki-cempd.vipapps.unc.edu/index.php/.

### 2.1 WRF and CMAQ meteorological and transport updates

The WRF and CMAQ models were updated to improve the representation of land-surface processes and vertical mixing. There were two changes made to the PX-LSM in WRF. First, the stomatal conductance function for photosynthetically active radiation (PAR) was revised based on measurements of net photosynthetic rate as a function of PAR for cotton plants, reported by Echer and Rosolem (2015). The new functions yield a significantly lower magnitude when shortwave radiation is less than 350 Wm^-2^. This in turn results in reduced latent heat flux and enhanced sensible heat flux, causing a delay in surface stabilization (prolongs mixing) during evening transition hours (i.e., sunset). This reduces overestimations (reduced positive bias) in water vapor mixing ratios, which are common in the WRF-CMAQ modeling system during the evening transition. Similarly, overestimation of concentrations of surface-emitted species (e.g., NO, NO_2_, CO and elemental carbon (EC)) are also reduced during the evening transition. This change was released in WRFv3.7 and further revised in WRFv3.8. The second change made to the PX-LSM is an increase of the coefficient to the surface energy forcing in the soil temperature force-restore equation (*C*_v_), which is related to volumetric heat capacity (*c*_v_) and heat conductivity (λ) (Pleim and Gilliam 2009) as follows:

(1) $$C_{v}=2{\left(\frac{\pi }{c_{v}\lambda \tau }\right)}^{\frac{1}{2}},$$

where τ is 1 day (86400 s), from the previous value of 8 × 10^−6^ Km^2^ J^−1^ recommended by Giard and Bazile (2000) to 1.2 ×10^−5^Km^2^ J^-1^. The new value for *C*_v_ results from updated values for *c*_v_ and λ or vegetation based on measurements of various leaves by Jayakshmy and Philip (2010) (*c*_v_ = 2.0 × 10^6^ Jm^−3^ K^−1^, λ = 0.5 Wm^−1^ K^−1^). These changes reduce overestimations of minimum 2 m temperature (i.e., warmer surface temperatures) during the early morning (dawn) hours while also reducing underestimations of 2m temperature during the post-dawn hours.

There were also two major revisions made to the ACM2 vertical mixing scheme in both WRF and CMAQ. In WRF, the ACM2 was updated to estimate and apply different eddy diffusivities for momentum (*K*_m_) and heat (*K*_h_) so that the Prandtl number (*Pr*) is no longer assumed to be unity *(Pr =* K_m_*/K*_*h*_ = 1). The second major modification to ACM2 is the implementation of new stability functions for both heat and momentum for stable conditions, which allows for more mixing in the stable regimes, particularly moderately stable conditions that often occur in the early evening hours. CMAQv5.1 has also been modified to include the same stability functions that are used in WRFv3.7, and therefore, for consistency, WRFv3.7 (or newer) and CMAQv5.1 should be used together. Both of these revisions to the ACM2 are described in Pleim et al. (2016).

The MOL values used in the ACM2 model in CMAQ were found to differ from the MOL values used in the ACM2 model in WRF. Specifically, the output from WRF was for a preliminary estimate of MOL that was computed in the surface layer model in WRF (module__sf__pxsfclay.F). The MOL was later re-computed in ACM2 in WRF but not loaded into the output array. This inconsistency has been fixed in v5.1 by re-computing the MOL in CMAQ exactly as it is computed in ACM2 in WRF. However, starting with WRFv3.8, this recomputed MOL value will be available in the WRF output, and therefore it will be unnecessary to re-compute the MOL value in CMAQ.

### 2.2 Scientific improvements in the CMAQv5.1 aerosol treatment

CMAQ has historically underestimated SOA in both urban (Woody et al., 2016) and rural (Pye et al., 2015) locations. Thus, improvements to the representation of aerosol from anthropogenic and biogenic hydrocarbons were needed. The updates to SOA formed from anthropogenic volatile organic compounds (VOCs) focus on VOCs in existing emission inventories, such as the EPA National Emissions Inventory (NEI), that are likely to fall in the intermediate VOC (IVOC) range. These include long-chain alkanes such as heptadecane and PAHs such as naphthalene. Since these compounds are much less volatile than traditional VOCs, they readily form aerosol in high yields. Long-chain alkanes and PAHs were included in other VOC categories in CMAQ versions prior to v5.1, but were lumped with smaller, more-volatile compounds that did not form SOA with the same efficiency. By separating long-chain alkanes and naphthalene at the emission processing step, CMAQ can better account for their higher yields. Several studies (e.g., Pye and Pouliot, 2012; Jathar et al., 2014) have indicated that a large fraction of VOC emissions, particularly IVOC-type compounds, may not be characterized in emission inventories, which limits how much SOA can be formed from anthropogenic VOCs in current CTMs.

Several new SOA species were introduced in v5.1 AERO6, specifically AALK1 and AALK2 (from long-chain alkanes) and APAH1, APAH2, and APAH3 (from naphthalene). CMAQv5.1-predicted alkane SOA is responsible for ~ 20 to 50% of SOA from anthropogenic VOCs, with the largest absolute concentrations occurring during summer in urban areas. Naphthalene oxidation is predicted to produce more modest amounts of SOA (Pye and Pouliot, 2012). Note that PAH SOA in v5.1 only considers naphthalene as the parent hydrocarbon, which is about half of the PAHs considered as SOA precursors in Pye and Pouliot (2012). This approach was used since naphthalene is a high-priority hazardous air pollutant (HAP) and necessary in the model for purposes other than SOA formation.

CMAQv5.1 has been updated to include the isoprene epoxydiol (IEPOX) SOA resulting from aqueous reactions for most chemical mechanisms including CB05 and SAPRC07, as described in Pye et al. (2013). Later-generation isoprene oxidation products formed under low-NO_*x*_ conditions, specifically IEPOX, are recognized as a significant source of SOA based on laboratory (Surratt et al., 2010), field (Hu et al., 2015) and modeling (McNeill et al., 2012; Pye et al., 2013; Marais et al., 2016) studies. This SOA is linked to sulfate and acidity and thus represents an anthropogenically controlled source of biogenic SOA.

In addition to the SOA updates for anthropogenic VOCs, AISO_3_ (acid-catalyzed isoprene epoxide aerosol) was also revised in CMAQv5.1 to represent SOA from IEPOX. For the CB05tucl, CB05e51 and SAPRC07 chemical mechanisms with IEPOX formation in the gas phase, heterogeneous uptake of IEPOX on acidic aerosol results in SOA (Pye et al., 2013). This IEPOX SOA replaces the AISO_3_ treatment based on Carlton et al. (2010). The AISO3J species name is now retained for IEPOX SOA and represents the sum of IEPOX- derived organosulfates and 2-methyltetrols. Explicit isoprene SOA species including 2-methyltetrols, 2-methylglyceric acid, organosulfates, and oligomers (e.g. dimers) are available in the SAPRC07tic with AERO6i mechanism now available in CMAQv5.1. See Table 1 for more information regarding these new SOA species.

### 2.3 Improvements to the CMAQv5.1 in-line photolysis and cloud model

The in-line calculation of photolysis rates in CMAQ has undergone significant changes. The calculation of photolysis rates in v5.1 still uses the same approach for calculating actinic fluxes by solving a two-stream approximation of the radiative transfer equation (Binkowski et al., 2007; Toon et al., 1989) over wavebands based on the FAST-J photolysis model (Wild et al., 2000). Each layer includes scattering and extinction using simulated air density, cloud condensates, aerosols and trace gaseous such as O_3_ and NO_2_. The first area changed in v5.1 is how clouds are described in the actinic flux calculation. In v5.0.2, a vertical column had a single cloud deck with constant cloud fraction, liquid water content and water droplets as the source of scattering and extinction from clouds. These cloud parameters were diagnosed from humidity and air temperature predicted by the meteorological model (e.g., WRF). CMAQv5.1 uses additional information available from WRF that describes the resolved cloud cover, which allows the vertical column to have multiple cloud decks with variable cloud fractions and multiple types of water condensates. In addition to the resolved cloud cover, v5.1 also includes the radiative effect from CMAQ’s subgrid convective clouds in the calculation of actinic fluxes. CMAQ uses the ACM cloud model to describe subgrid convective clouds based on convective precipitation rates from WRF. These updates to the clouds used in the photolysis rates improved CMAQ’s internal consistency between cloud mixing, aqueous chemistry and gas-phase chemistry.

The second area of change to the in-line photolysis calculation addressed the radiative effect from aerosols. The mixing model used to compute the refractive indices of aerosol modes (an internal-volume weighted average model) allows the refractive index of each aerosol component to depend on wavelength. Most importantly, the refractive index for elemental (black) carbon reflects the current scientific consensus (Bond and Bergstrom, 2006; Chang and Charalam-popoulos, 1990; Segelstein, 1981; Hess et al., 1998) and increases its absorptive capacity from the v5.0.2 value. Additionally, estimating aerosol optical properties includes new options to solve Mie scattering theory, or the option to use the core-shell model with an elemental carbon core (Bohren and Huffman, 2004). A user can choose to use these options by setting environment variables before executing the CMAQ model (http://www.airqualitymodeling.org/). By default, v5.1 uses approximate solutions to Mie scattering and the internal-volume weighted average model (Binkowski et al., 2007). Third, several new variables (e.g., resolved cloud fraction, subgrid cloud fraction, resolved cloud water content) have been added to the cloud diagnostic file that describe the optical properties of aerosol and clouds and their radiative effects.

### 2.4 Improvements in CMAQv5.1 atmospheric chemistry

Several changes were made to the CB05TUCL chemical mechanism in v5.1 (Whitten et al., 2010; Sarwar et al., 2012), which is now referred to as CB05e51. These changes include updates to reactions of oxidized nitrogen (NO_*y*_) species, incorporation of new research on the atmospheric reactivity of isoprene photooxidation products, addition of several high-priority HAPs to the standard CB05e51 mechanism (following the protocol in the multipollutant version of CMAQ), and other changes to update the mechanism and make it compatible with updates to the aerosol chemistry, but overall retaining the fundamental core of the CB05 mechanism. A more detailed explanation of the changes made in the CB05e51 mechanism is provided below.

#### 2.4.1 NO_*y*_ updates and additions

The most extensive changes made consisted of updates and extensions of the NO_*y*_ species, including peroxyacyl nitrates, alkyl nitrates, and NO_*x*_ reactions with HO_*x*_. The thermal formation and degradation of peroxyacetyl nitrate (PAN) were modified to correct the parameters that describe the rate constant pressure dependence in the fall-off region between the high-pressure limit and the low-pressure limit based on the values determined by Bridier et al. (1991). An additional species, MAPAN, was added to explicitly represent PANs from methacrolein because these are a possible contributor to SOA formation. The OH + NO_2_ reaction rate was updated based on Troe (2012), and a small yield of HNO3 (< 1 % at standard temperature and pressure, varying with temperature and pressure) was added to the reaction of HO2 + NO (Butkovskaya et al., 2007). The single alkyl nitrate species in CB05, NTR, was replaced with seven species to better investigate the variety of chemical and physical fates of alkyl nitrates. The first-generation monofunctional alkyl nitrates and difunctional hydroxy nitrates were assigned Henry’s law constants of 6.5 × 10^−1^ and 6.5 × 10^3^ M, respectively, while second-generation carbonyl nitrates were assigned 1.0 × 10^3^ M and multifunctional hydroxy nitrates were assigned a value of 1.7 × 10^4^ M. Five species are predominantly from anthropogenic sources, with the relative distribution of monofunctional (alkyl nitrates) and multifunctional (hydroxy, carbonyl, hydroxycarbonyl, and hydroperoxy) nitrate products determined based on the nitrates produced from the five alkanes and alkenes, with the largest emissions as listed in the NEI (Simon et al., 2010). The other two nitrate species represent first-generation and later-generation nitrates from biogenic (isoprene and terpene) sources. Biogenic nitrate products were based on reaction products from Lee et al. (2014), with NO_*x*_ recycling from secondary biogenic nitrate products (Jenkin et al., 2015) and photolysis rates with quantum yields of unity. Finally, a heterogeneous hydrolysis rate of alkyl nitrates was added (Hildebrandt-Ruiz et al., 2013), with a 6 h lifetime on aerosol at high relative humidity (Liu et al., 2012; Rollins et al., 2013). Additional details can be found in the CMAQv5.1 release documentation (http://www.airqualitymodeling.org/).

#### 2.4.2 Other changes

The high HO_*x*_ pathways for isoprene oxidation have been modified to explicitly account for production of IEPOX, which can form SOA and modify the gas-phase concentrations. The high-NO_*x*_ pathways have been modified to explicitly produce methacrolein PAN (MAPAN, described in Sect. 2.4.1) because it reacts faster with OH than other PAN species. Several high-priority HAPs were added to the standard version of CB05e51 as either active species or reactive tracers, specifically acrolein, 1,3-butadiene (which produces acrolein), toluene, xylene isomers,*α* - and *β*-pinene, and naphthalene, using reaction pathways and rates as defined by IUPAC. Refer to the CMAQv5.1 release documenation for additional details on these updates.

Several other, smaller changes were made to the chemistry to either improve consistency with IUPAC, enhance the integration with heterogeneous chemistry, or for numerical consistency. These include the following: the updates to the products of ethanol reaction with OH using recommended yields from IUPAC (http://iupac.pole-ether.fr; accessed 11 May 2016); updates to the reactions of acylperoxy radicals with HO_2_ to include a 44 % yield of OH; the addition of a new species, SOAALK, to account for SOA formation from alkanes; and the addition of gas-phase and heterogeneous nitryl chloride formation (ClNO_2_) and CINO_2_ photolysis as described by Sarwar et al. (2012).

### 2.5 Updates to air-surface exchange processes in CMAQv5.1

Meteorologically dependent emissions and deposition, hereafter referred to as air-surface exchange, were extensively updated in v5.1. A data module was developed to share meteorological and calculated atmospheric transport environmental variables between vertical diffusion, deposition and meteorologically dependent emissions to more consistently represent processes common to both deposition and emissions. Additionally, sea-salt and biogenic emissions as well as dry deposition routines were updated.

#### 2.5.1 Sea-salt aerosol emission

The sea-salt aerosol emissions module was updated to better reflect emission estimates from recent field observations and to incorporate ocean thermodynamic impacts on emissions. The size distribution of sea-salt aerosol was expanded to better reflect recent fine-scale aerosol measurements in laboratory and field studies (de Leeuw et al., 2011) by modifying the O parameter of Gong (2003) from 30 to 8. A sea- surface temperature (SST) dependency to the sea-salt aerosol emissions following Jaeglé et al. (2011) and Ovadnevaite et al. (2014) was also added, which increased accumulation and coarsemode sea-salt emissions in regions with high SSTs and reduced the emissions in regions with low SSTs. Finally, the surf-zone emissions of sea-salt aerosol were reduced by 50 %, assuming a decrease in the surf-zone width from 50 to 25 m to address a systematic overestimation of near-shore coarse sea-salt aerosol concentrations (Gantt et al., 2015).

#### 2.5.2 Biogenic emissions

There were also several updates to the calculation of nonmethane biogenic volatile organic carbon (BVOC) emissions in v5.1. The Biogenic Emissions Inventory System (BEIS; https://www.epa.gov/) model was updated to include the implementation of a dynamic two-layer, sun and shaded, vegetation canopy model, while the PAR response function was integrated into the canopy model following Niinemets et al. (2010) for each canopy layer. In earlier versions of BEIS, emissions were a function of the 2 m temperature which was inconsistent with measured emission factors that were empirically correlated with leaf temperature. BEIS 3.6.1, released with v5.1, was updated to model emissions as a function of the leaf temperature rather than 2 m temperature to be more consistent with how BVOC emission factors are typically estimated. For additional details see Bash et al. (2016). Finally, the Biogenic Emission Land-use Data (BELD) version 4.0 and emission factors for herbaceous wetlands were updated to address overestimates of BVOCs at coastal sites (Guenther et al., 2006), and the BELD land-use and vegetation species were updated using high-resolution satellite data and in situ survey observations from 2002 to 2012 (Bash et al., 2016).

#### 2.5.3 Dry deposition

There were two important updates to the dry deposition calculation in v5.1. First, the dry deposition of O_3_ over oceans was updated to include the additional sink due to interaction with iodide in the seawater (marine halogen chemistry), with the iodide concentrations estimated based on sea-surface temperature (Sarwar et al., 2015), which increased the O_3_ deposition velocity over oceans. Second, over vegetative surfaces, the wet cuticular resistance was updated following Altimir et al. (2006) (385 sm^−1^), and dry cuticular resistance was set to the value of Wesley (1989) for lush vegetation (2000 sm^−1^). These changes resulted in an approximately 2.0 ppbv reduction in the modeled O_3_ mixing ratios, with the largest reductions, ~ 10 %, occurring during the nighttime and early morning hours, and approximately a 2 % reduction in the modeled midday O_3_ mixing ratio. It was later discovered (after the release of v5.1) that the 385 s m^−1^ value represents a canopy resistance rather than a leaf resistance, and therefore should be closer to a value of 1350 s m^−1^ following Altimer et al. (2006). The value will be corrected in the next CMAQ model release.

#### 2.5.4 Gravitational settling

Previous evaluations of the ground-level coarse particle (PM_1_o-PM_2.5_) concentrations in CMAQ have shown that the model significantly underestimated the total PM_10_ concentrations (Appel et al., 2012). Contributing to this underestimation is the fact that CMAQ previously did not have a mechanism in place to allow coarse particles to settle from upper layers to lower layers (although coarse particles in layer one can settle to the surface). As a result, large particles that would normally settle to lower layers in the model could remain trapped in the layers in which they were emitted or formed. To account for this deficiency in the model, the effects of gravitational settling of coarse aerosols from upper to lower layers have been added to v5.1 to more realistically simulate the aerosol mass distribution. The net effect of this update is an increase in ground-level PM_10_ concentrations in v5.1 compared to v5.0.2, particularly near coastal areas impacted by sea spray (Nolte et al., 2015).

As stated in the beginning of this section, but is useful to reiterate here, the information provided in this section only covers a portion of the vast number of updates that went into v5.1, and was intended to make the reader aware of the more significant changes made and why, but often avoids including the very specific detailed code changes that were made to the model. Those seeking a complete detailed list of all the changes made to the model should refer to the v5.1 technical documentation using the link provided at the beginning of this section.

## 3 Modeling setup and observational data sets

The modeling setup for the evaluation of v5.1 utilizes a domain covering the entire contiguous United States (CONUS) and surrounding portions of northern Mexico and southern Canada, as well as the eastern Pacific and western Atlantic oceans. The modeling domain consists of 299 north-south by 459 east-west grid cells utilizing 12 km × 12 km horizontal grid spacing, 35 vertical layers with varying thickness extending from the surface to 50 hPa and an approximately 10 m midpoint for the lowest (surface) model layer. The simulation time period covers the year 2011, which is a base year for the EPA’s NEI and also a period during which specialized measurements from a variety of trace species are available from the Deriving Information on Surface Conditions from Column and Vertically Resolved Observations Relevant to Air Quality (DISCOVER-AQ; http://www.nasa.gov/) campaign.

All the CMAQ simulations presented here employed the Euler backward iterative (EBI) solver. The v5.0.2 simulations utilized the windblown dust treatment available, while the v5.1 simulations did not due to errors in the implementation of the windblown dust model in v5.1. However, the overall contribution of windblown dust to PM_2.5_ is small on a seasonal average and does not affect the seasonal comparisons shown in Sect. 5. Additional details regarding the options employed in the CMAQ simulations are available upon request from the corresponding author. For the annual simulations, a 10-day spin-up period in December 2010 was used (and then discarded) to reduce the effects of the initial conditions, after which the model was run continuously for the entire year 2011 (one continuous simulation stream). For the 1-month January and July sensitivity simulations presented, 10-day spin-up periods in the previous month were used and then discarded. Boundary conditions for the 12 km CMAQ simulations are provided by a 2011 hemispheric GEOS-Chem (Bey et al., 2001) with the chemical species mapped to the corresponding CMAQ species.

Several sets of CMAQ simulations were performed to help thoroughly evaluate both the overall change in model performance between v5.0.2 and v5.1 and to examine the individual impact of specific model process changes on the model performance. As such, different input data sets were used for the v5.0.2 and v5.1 simulations. The base v5.0.2 simulation (CMAQv5.0.2_Base) utilized WRFv3.4 meteorological input data, while WRFv3.7-derived meteorological data were used for all the v5.1 simulations presented here. As stated previously, different versions of WRF were used for the v5.0.2 and v5.1 simulations due to the updates made in both WRF and CMAQ (Sect. 2.1) that would have made performing the CMAQ simulations with output from the same version of WRF difficult and introduced some inconsistencies. While there were other updates made to WRF between versions 3.4 and 3.7, those changes were minor and did not impact the WRF results significantly for the configuration of the model used here.

Both WRF simulations employed the same options, which include the Rapid Radiation Transfer Model Global (RRTMG) long-wave and shortwave radiation (Iacono et al., 2008), Morrison microphysics (Morrison et al., 2005), and the Kain-Fritsch version 2 cumulus parametrization (Kain, 2004). For the LSM and PBL models, the PX-LSM and ACM2 were used. Four-dimensional data assimilation (FDDA) was also employed in the WRF simulations. The name lists used for each WRF simulation are provided in the Supplement (see Sects. 4 and 5). Model-ready meteorological input files were created using version 4.1.3 of the meteorology-chemistry interface processor (MCIP; Otte and Pleim, 2010) for the WRFv3.4 data and MCIP version 4.2 (https://www.cmascenter.org/) for the WRFv3.7 data.

Two sets of emission input data were utilized for the analysis presented here. Both sets of emission data were based on the 2011 NEI, with version 1 (v1) of the 2011 NEI modeling platform developed by the USEPA from regulatory applications (https://www.epa.gov/) utilized for the majority of the simulations, while version 2 (v2) of the 2011 modeling platform was utilized for one set of sensitivity simulations. However, all the comparisons of model simulations presented here are shown with simulations that utilized the exact same emissions inventory, and as such any differences in model performance are not the result of differences in emissions. See Table 2 for information regarding which version of the emission inventory was utilized for each simulation.

The raw emission files were processed using versions (v1 emissions) and 3.6.5 (v2 emissions) of the Sparse Matrix Operator Kernel Emissions (SMOKE; https://www.cmascenter.org/smoke/) program to create gridded speciated hourly model-ready input emission fields for input to CMAQ. Electric generating unit (EGU) emissions were obtained using data from EGUs equipped with a continuous emission monitoring system (CEMS). Plume rise for point and fire sources were calculated in-line for all simulations (Foley et al., 2010; https://www.cmascenter.org/). Biogenic emissions were generated in-line in CMAQ using BEIS versions 3.14 for v5.0.2 and 3.61 (Bash et al., 2016) for v5.1. All the simulations employed the bidirectional (bi-di) ammonia flux option for estimating the air-surface exchange of ammonia, as well as the in-line estimation of NO_*x*_ emissions from lightning strikes.

Output from the various CMAQ simulations is paired in space and time with observed data using the atmospheric model evaluation tool (AMET; Appel et al., 2011). There are several regional and national networks that provide routine observations of gas and particle species in the US. The national networks include the EPA’s Air Quality System (AQS; 2086 sites; https://www.epa.gov/aqs) for hourly and daily gas and aerosol PM species; the Interagency Monitoring of Protected Visual Environments (IMPROVE; 157 sites; http://vista.cira.colostate.edu/improve/) and Chemical Speciation Network (CSN; 171 sites; https://www3.epa.gov/ttnamti1/speciepg.html) for daily average (measurements typically made every third or sixth day) total and speciated aerosol PM species; and the Clean Air Status and Trends Network (CASTNET; 82 sites; http://www.epa.gov/castnet/) for hourly O_3_ and weekly aerosol PM species. In addition to these routinely available observations, the DISCOVER-AQ campaign (https://www.nasa.gov/mission_pages/discover-aq/) during July 2011 provides additional ground-based gas and aerosol PM measurements, along with unique aloft measurements made by aircraft, vertical profilers (e.g., light detection and ranging (lidar) measurements), ozonesondes and tethered balloons (not utilized in this analysis, however).

## 4 Evaluation of major scientific improvements

In this section we evaluate the impact that several of the major scientific improvements in v5.1 have on the operational model performance. Unlike Foley et al. (2010), in which several individual major scientific improvements in CMAQ v4.7 were evaluated incrementally (e.g., each subsequent improvement is evaluated against the previous improvement), here we examine each scientific improvement separately by comparing simulations with the specific improvement removed (i.e., as it was in v5.0.2) to the base v5.1 simulation (CMAQv5.1_Base_NEIv1) which includes all the updates. While this has the disadvantage of not showing the incremental change in model performance due to each improvement, it does limit the number of simulations that need to be performed. In addition, it allows for easier examination of the effect of nonlinear increments on total model performance, as some updates to the modeling system may be affected by updates to other parts of the model, the effects of which on model performance may not be captured in an incremental testing format. Note that while some attempt is made to broadly identify the processes involved that cause the observed changes in model performance between v5.0.2 and v5.1, it would be too laborious (both to the reader and to the investigators) to comprehensively describe and investigate in depth the processes involved that result in each observed difference in model performance described in this section. Where appropriate, the analyses presented in this section use the v5.0.2 base simulation (CMAQv5.0.2_Base) for comparison to the scientific improvement while for other improvements the v5.1 base simulation is used for comparison. In each case, the simulations being compared are noted. Table 2 provides a description of the CMAQ model simulations referred to in the following sections.

### 4.1 WRF and CMAQ meteorological updates

As discussed in Sect. 2.1, there were several significant corrections and improvements made to the meteorological calculations in both WRF and CMAQ. While the focus of this work is on updates to the CMAQ model, certain options within WRF and CMAQ are linked, and therefore it is necessary to discuss the WRF model updates alongside the corresponding CMAQ model updates.

**Figure 1.**
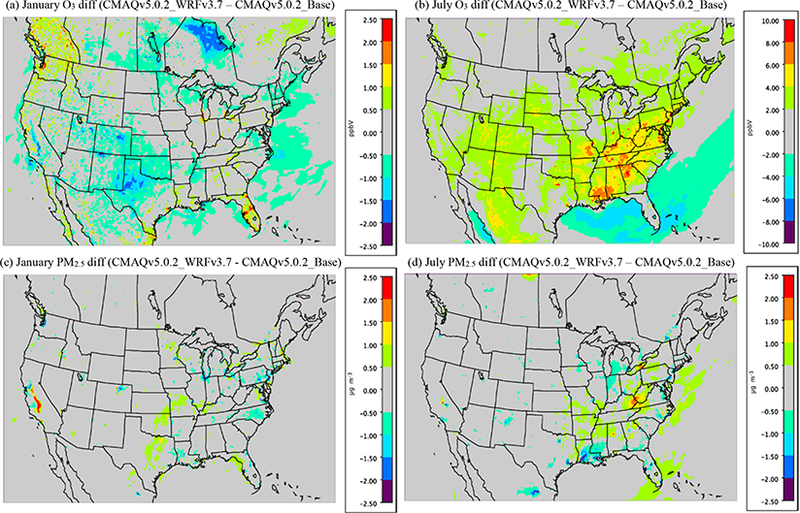
Monthly average difference in O_3_ (ppbv) for **(a)** January and **(b)** July and PM_2.5_ (μgm-^3^) for **(c)** January and **(d)** July between CMAQv5.0.2 using WRFv3.4 (CMAQv5.0.2_Base) and CMAQv5.0.2 using WRFv3.7 (CMAQv5.0.2_WRFv3.7) (CMAQv5.0.2_WRFv3.7-CMAQv5.0.2_Base). Note that the scales between each plot may vary.

Figure 1 shows the cumulative impact that all the meteorological changes in WRF and CMAQ (i.e., changes to ACM2 and MOL) had on O_3_ and PM_2.5_ in January and July by comparing the CMAQv5.0.2_Base simulation to a CMAQv5.0.2 simulation using WRFv3.7 (CMAQv5.0.2_WRFv3.7) which includes the ACM2 and MOL updates. The effect of the changes on O_3_ in January is mixed, with some areas (e.g., Florida, Chicago and the northwest) showing a relatively large (2.5 ppbv) increase in O_3_, while other areas (e.g., the southwest and Texas panhandle) show a relatively large decrease (−2.5 ppbv) in O_3_. For PM_2.5_, the differences in January are generally small and isolated; however, there is a relatively large increase in PM_2.5_ (> 2.5 μgm^−3^) in the San Joaquin Valley (SJV) of California due to the updates, which, combined with the decrease in O_3_ there as well, indicates a likely reduction in PBL height and mixing as the cause. There are also some relatively large decreases (1.5μg m^−3^) in PM_2.5_ in the northeast and around in the Great Lakes region (i.e., Chicago). Otherwise, most of the remaining impacts on PM_2.5_ are relatively small (< 1.0 pgm^−3^).

For July, the meteorological updates in WRF and CMAQ result in exclusively increased O_3_ mixing ratios over land, which are considerably larger than the impacts observed in January. The largest increases (4.0–10.0 ppbv) occur in the eastern US, particularly in the southeast. Smaller increases of 2.0–4.0 ppbv occur across much of the US, while in the Gulf of Mexico and the Caribbean O_3_ mixing ratios decrease roughly 2.0–6.0 ppbv across a large area. The difference in PM_2.5_ in July is similar to that in January, with mostly small, isolated increases or decreases occurring in the eastern US. The largest increase (2.0–2.5 μg m^−3^) occurs in the southern Ohio Valley (Kentucky and West Virginia), while the largest decreases (> 2.5 pg m^−3^) occur in Louisiana and Texas (i.e., Houston).

It makes intuitive sense to see summertime O_3_ mixing ratios increasing due to the meteorological changes in WRF and CMAQ, since the net effect of those changes was to increase mixing, particularly in the late afternoon and early evening, which in turn decreases the amount of NO titration of O_3_ that occurs in the model, and ultimately results in higher O_3_ mixing ratios on average. Conversely, PM_2.5_ concentrations would be expected to decrease due to the increased mixing in the model, which would effectively decrease the concentrations of primary emitted pollutants (e.g., EC and organic carbon (OC)), which was observed in areas with the largest emissions (i.e., urban areas). In addition, changes in the oxidant (i.e., OH) concentrations would also potentially affect PM_2.5_ concentrations through increased or decreased SOA formation (spatial heterogeneity of PM_2.5_ formation), which results in spatially varying increases and decreases in PM_2.5_ concentrations.

### 4.2 Aerosol updates

**Figure 2.**
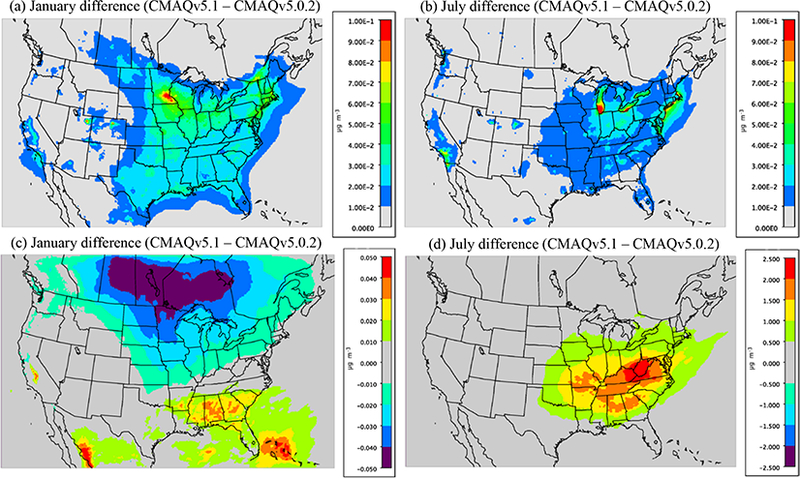
Monthly average sum total of AALK1, AALK2, APAH1, APAH2 and APAH3 for **(a)** January and **(b)** July (upper right) and the monthly average difference is the sum total of AISO1, AISO2, AISO3 and AOLGB for **(c)** January and **(d)** July between the aerosol treatments in CMAQ v5.0.2 and v5.1 (v5.1-v5.0.2). All plots are in units of micrograms per cubic meter (μgm^−3^). Note that the scales between each plot may vary.

**Figure 3.**
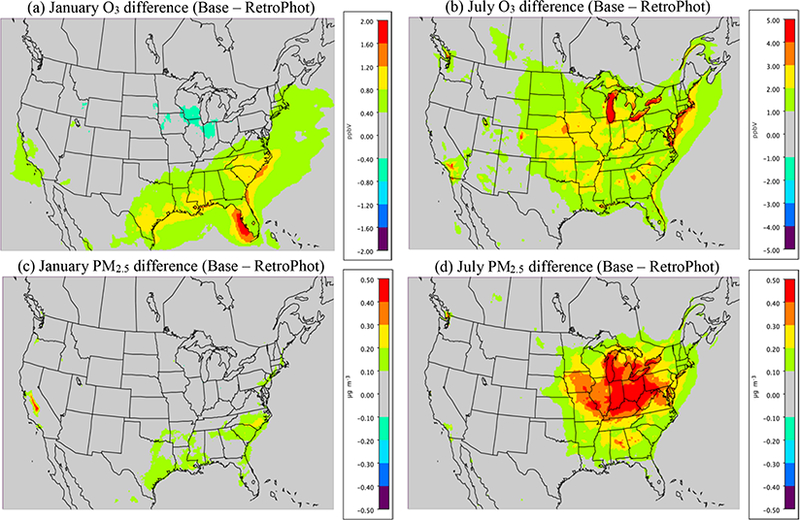
Difference in the monthly average O_3_ for **(a)** January and **(b)** July and PM_2.5_ for **(c)** January and **(d)** July between CMAQv5.1_base and v5.1_RetroPhot (v5.1_Base - v5.1_RetroPhot). O_3_ plots are in units of parts per billion by volume (ppbv) and PM_2.5_ plots are in units of micrograms per cubic meter (μg m^−3^). Note that the scales between each plot may vary.

Several new SOA species from anthropogenic VOCs (i.e., AALK1, AALK2, APAH1, APAH2 and APAH3; Table 1) were added to AERO6 in v5.1 that were not present in v5.0.2. Figure 3 shows the difference in the monthly average sum total concentration of these five species for January and July 2011 between the CMAQv5.0.2_Base and CMAQv5.1_Base simulations. Since none of these species were present in v5.0.2, the difference totals in Fig. 2 represent the additional SOA mass that these five species contribute to the total PM_2.5_ mass in v5.1. For both January and July, the monthly average concentration of these species is small, ranging between 0.0 and 0.1 μgm^−3^, with the largest concentrations in the eastern half of the US, particularly in the upper Midwest. However, the concentration of these new species during shorter time periods and smaller, isolated regions would be larger. In addition, the inclusion of these new species is potentially important for health-related studies on the impact of PAHs. Overall, however, these new species represent a small addition to the total PM_2.5_ concentration in the model.

Along with the introduction of the new SOA species above, the pathways for the formation of acid-enhanced isoprene SOA were also updated. The bottom panels in Fig. 2 show the monthly average difference in the sum of the species containing isoprene SOA (AISO1, AISO2, AISO3 and AOLGB) between v5.1 and v5.0.2. For January, the difference in the sum of these species is relatively small, with minimum and maximum values peaking around ±0.5 μg m^−3^, consistent with the fact that isoprene emissions are low in winter. For July the difference is always positive (v5.1 higher than v5.0.2) and much larger compared to January, with peak differences exceeding 2.5 μgm^−3^, primarily in the areas with the highest aerosol ${\mathrm{SO}}_{4}^{2-}$ concentrations (i.e., Ohio Valley). Therefore, the updated IEPOX-SOA formation pathways in v5.1 represent a potentially significant contribution to the total PM_2.5_, particularly during the summer. Increased isoprene emissions in v5.1 with BEISv3.61 compared to v5.0.2 with BEISv3.14 also contribute to the larger contribution of isoprene SOA in v5.1.

### 4.3 Cloud model and in-line photolysis updates

Changes in the photolysis and cloud model treatment in v5.1 have potentially significant impacts on the O_3_ and PM_2.5_ estimates from the model. Figure 3 shows the difference in O_3_ and PM_2.5_ for the CMAQv5.1_Base simulation and the CMAQv5.1_RetroPhot simulation (see Table 2 for simulation description). The CMAQv5.1_RetroPhot simulation is the same as the CMAQv5.1_Base simulation except it employs the same (old) photolysis and cloud model treatment as in v5.0.2. For January, O_3_ mixing ratios (Fig. 3a) and PM_2.5_ concentrations (Fig. 3c) are both higher across the southeast and portions of California in the v5.1 simulation, indicating that v5.1 has much less photolysis attenuation due to the updates in the representation of cloud effects on photolysis.

The impact of the updated photolysis in v5.1 is considerably larger in July (when there is more convection) than in January. Peak O_3_ differences in January were around 2.0 ppbv, whereas in July peak differences of greater than 5.0 ppbv (Fig. 3b) occur over the Great Lakes (where low PBL heights can enhance the impact of changes in O_3_). However, in general the difference in O_3_ mixing ratios is larger in both magnitude and spatial coverage in July compared to January, indicating that the updated photolysis and cloud model treatment in v5.1 increases O_3_ to a greater extent in July compared to January, as expected due to increased photolysis rates in the summer compared to winter. Overall, differences in O_3_ in July range on average from 1.0 to 3.0 ppbv, with larger differences occurring in the major urban areas (e.g., Atlanta, Charlotte and Los Angeles) and off the coast of the northeast corridor. The change in PM_2.5_ is also larger (both in magnitude and spatial coverage) in July than January (Fig. 3d). The greatest change is primarily confined to the eastern US, resulting in a roughly 0.1 to 0.5 μg m^−3^ increase in PM_2.5_ in v5.1, with the maximum increase located over the Great Lakes region and areas to the south, the result of increased SOA and gas-phase production of ${\mathrm{SO}}_{4}^{2-}$ due to greater OH^-^ concentrations in v5.1.

**Figure 4.**
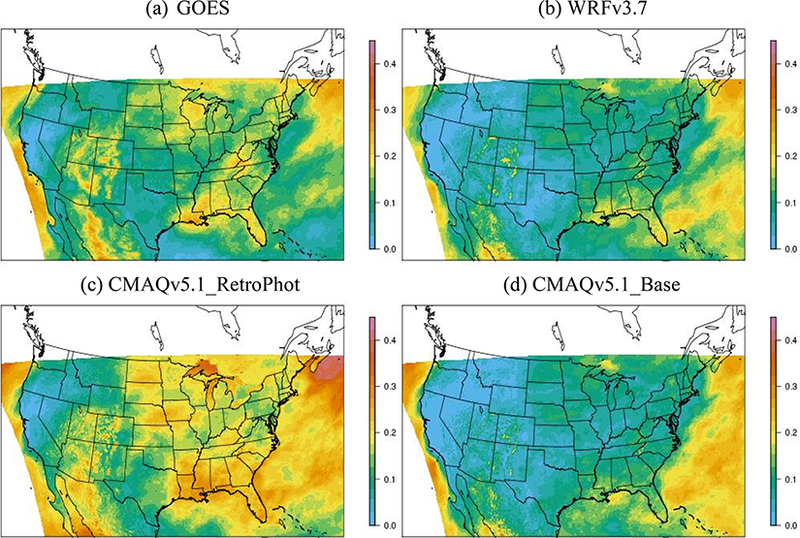
The average cloud albedo during daytime hours in July 2011 with available satellite data (*n* =301 h total) derived from **(a)** the GOES satellite product, **(b)** WRF3.7, **(c)** CMAQv5.1 with photolysis and cloud model treatment from v5.0.2 and WRF3.7 inputs (CMAQv5,1 _RetroPhot), and **(d)** CMAQv5.1 using WRF3.7 inputs (CMAQv5.1_Base_NEIv2).

Additional diagnostic evaluation of photolysis and cloud model treatment in CMAQ was conducted based on the model-predicted cloud albedo at the top of the atmosphere. The predicted cloud albedo from WRFv3.7, CMAQv5.0.2 and CMAQv5.1 were evaluated against cloud albedo from NASA’s Geostationary Operational Environmental Satellite (GOES) Imager product. This evaluation was used to qualitatively determine whether one CMAQ version better considers how clouds affect calculated photolysis rates. The GOES product has a 4 km horizontal resolution and was re-gridded to the 12 km grid structure used in the WRF and CMAQ simulations using the Spatial Allocator utility (https://www.cmascenter.org/sa-tools/). The satellite data are available at 15 min prior to the top of the hour during daytime hours and were matched to model output at the top of the hour. There were 301 h with available satellite data across the domain in July 2011. Figure 4 shows the average cloud albedo (i.e., reflectivity at the top of the atmosphere) during these 301 h in July derived from the GOES 35 satellite product (Fig. 4a), and the cloud parameteri- zations within: WRF3.7 (Fig. 4b), CMAQv5.1_RetroPhot (Fig. 4c) and CMAQv5.1_Base_NEIv2 (Fig. 4d). Comparison of Fig. 4b and c shows the dramatic differences between the clouds predicted by WRFv3.7 and the predictions from the cloud parameterization in v5.0.2. Most of these large differences, particularly over land, are now gone in model predictions from the CMAQv5.1_Base simulation, which uses resolved clouds from WRF and subgrid clouds from the convective cloud model within CMAQ (compare Fig. 4b to d).

Two notable issues remain with the v5.1 modeled cloud parametrization. The photolysis cloud parameterization in v5.1 produces more clouds over water compared to the WRF parameterization, which is itself biased high for some parts of the Atlantic Ocean compared to GOES. This issue will be addressed by science updates planned for the CMAQ system, and evaluation results are expected to improve in the next CMAQ release. A more significant issue, from an air quality perspective, is the underprediction of clouds over much of the eastern and west-central US in the WRF-predicted clouds, which is now directly passed along to CMAQ. This misclas-sification of modeled clear sky conditions can contribute to an overprediction of O_3_ in these regions. Resolving this issue will require changes to the WRF cloud parameterization. Future research will also include changing the subgrid cloud treatment currently used in the CMAQ system to be consistent with the subgrid parameterization used in WRF. Section S1 in the Supplement provides a table with additional evaluation metrics of the modeled clouds over oceans vs. over land and also describes how cloud albedo was calculated for the three model simulations.

### 4.4 Atmospheric chemistry updates

As detailed in Sect. 2.4, numerous updates were implemented in the representation of atmospheric chemistry in v5.1. It would be extremely cumbersome to attempt to isolate the impact of each chemistry update individually. Instead, in order to assess the overall impact that the combined chemistry changes have on the model results, model comparisons are conducted using the CMAQv5.1_Base simulation, which employs the CB05e51 chemical mechanism (the v5.1 default chemical mechanism) and the CMAQv5.1_TUCL simulation (see Table 2 for description). The CMAQv5.1_TUCL simulation is the same as the CMAQv5.1_Base simulation except that it employs the CB05TUCL chemical mechanism (Whitten et al., 2010; Sarwar et al., 2012), the default mechanism in v5.0.2. Note that the aerosol updates discussed in Sect. 4.2 were incorporated into the CB05e51 chemical mechanism (in the past that portion of the aerosol chemistry was separate from the gas-phase chemical mechanism). As such, differences between the CMAQv5.1_TUCL and CMAQv5.1_Base_NEIv2 simulations include impacts from those changes (i.e., Fig. 2). In order to isolate primarily just the effect on PM_2.5_ from the atmospheric chemistry changes, the organic matter (ΑΟΜΠ; see Sect. 2 and 3 for species definition descriptions) mass has been removed from the comparisons of total PM_2.5_ mass discussed below.

**Figure 5.**
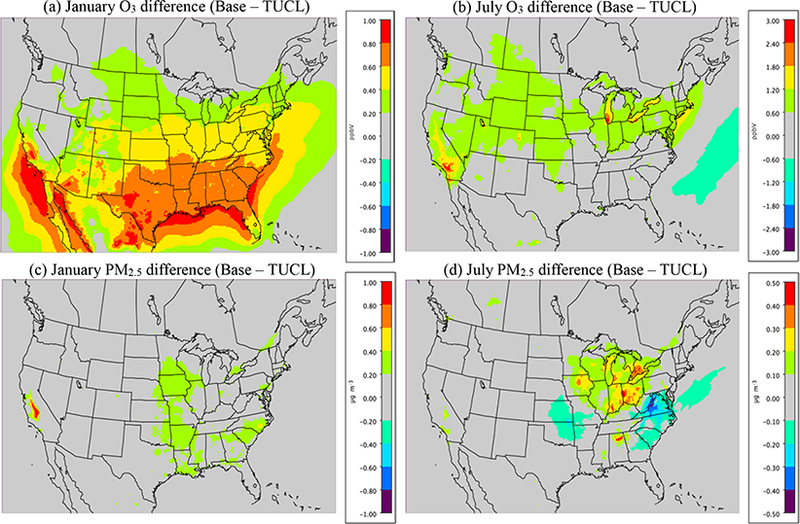
Difference in the monthly average O_3_ for **(a)** January and **(b)** July and PM_2.5_ (with organic matter mass removed) for **(c)** January and **(d)** July between CMAQv5.1_Base_NEIv2 and v5.1_TUCL (CMAQv5.1_Base_NEIv2-CMAQv5.1_TUCL). O_3_ plots are in units of parts per billion by volume (ppbv) and PM_2.5_ plots are in units of micrograms per cubic meter (μg m^−3^). Note that the scales for each plot can vary.

Figure 5 shows the difference in monthly average O_3_ and PM_2.5_ for January and July between the CMAQv5. l_Base_NEIv2 and CMAQv5.1_TUCF simulations. For January, O_3_ mixing ratios are higher in the simulation using the CB05e51 mechanism (CMAQv5.1_Base_NEIv2 simulation); however, the overall impact of CB05e51 on O_3_ is generally small (~ 2–4 %), with maximum differences of only approximately l.0 ppbv (~6 %), primarily along the southern coastal areas of the US. PM_2.5_ is also higher in January in the simulation using the CB05e51 mechanism (CMAQv5.1_Base_NEIv2 simulation), with the largest changes in PM_2.5_ of 0.2–0.4 μg m^−3^ (~ 2–6 %) primarily occurring in the eastern US and greater than l.0 μ gm^−3^ (~ 6–8 %) in the SJV of California.

For July, O_3_ mixing ratios are higher across most areas in the CMAQv5.1_Base_NEIv2 simulation, primarily across northern portions of the US, the Great Fakes region and in California (i.e., Eos Angeles and the SJV). Most increases in O_3_ in the CMAQv5.1_Base simulation range between 0.6 and 1.2 ppbv (~2–4 %); however, larger increases of over 3.0 ppbv (~4–8%) occur in southern California and over Fake Michigan (likely influenced in part by low PBF heights over the lake). A small area of lower O_3_ mixing ratios occurs off the eastern coast of the US. For July, the difference in PM_2.5_ due to the CB05e51 chemical mechanism is relatively small, with differences in concentrations generally ranging from ±0.5 μg m^−3^ (~2–4 %) across the eastern US.

## 5 Evaluation of CMAQv5.1

In this section, comparisons are made of the perfonnance of the CMAQv5.0.2_Base and CMAQv5.1_Base_NEIvl simulations by initially comparing the simulations to each other (model to model) and then evaluating them against a wide variety of available air quality measurements (see Sect. 3). Several common measurements of statistical performance are used, namely mean bias (MB), mean error (ME), root mean square error (RMSE) and Pearson correlation. Note that representativeness (incommensurability) issues are present whenever gridded values from a deterministic model such as CMAQ are compared to observed data at a particular point in time and space, as deterministic models calculate the average outcome over a grid for a certain set of given conditions, while the stochastic component (e.g., subgrid variations) embedded within the observations cannot be accounted for in the model (Swall and Foley, 2009). These issues are some what mitigated for networks that observe for longer durations, for example the CSN and IMPROVE networks, which are daily averages, and the CASTNET observations, which are weekly averages. The longer temporal averaging helps reduce the impact of stochastic processes, which can have a large impact on shorter (e.g., hourly) periods of observation (Appel et al., 2008).

There are a couple of important differences to keep in mind between the comparison of the CMAQv5.0.2_Base and CMAQv5.1_Base_NEIv1 simulations beyond the obvious changes to the model process representations discussed in the previous sections. First, the simulations use different versions of WRF (as discussed in Sects. 2.2 and 4.1). This was intentional, as it was determined that the changes made from WRFv3.4 (used in the CMAQv5.0.2_Base simulation) to WRFv3.7 (used in the CMAQv5.1_Base_NEIv1 simulation) and subsequent required changes made to the CMAQ code represent a change to the overall WRF-CMAQ modeling system and therefore should be evaluated together. It should also be noted that the windblown dust treatment was employed in the CMAQv5.0.2_Base simulation but not in the CMAQv5.1_Base_NEIv1 simulation. This was due to issues with the implementation of the updated windblown dust treatment in v5.1 that were not discovered until after the model was released and the CMAQv5.0.2_Base simulation was completed. However, the contribution of windblown dust to total PM_2.5_ in v5.0.2 tends to be small and episodic and therefore should not constitute a significant impact to the performance differences between v5.0.2 and v5.1, especially for the monthly averages generally shown here. However, we make an attempt to note when and where the impact from windblown dust is apparent. For reference, the v5.0.2-simulated seasonal average values of PM_2.5_ and maximum daily 8 h average (MDA8) O_3_ are provided in Figs. S1 and S2 in the Supplement, respectively.

### 5.1 PM_2.5_

**Figure 6.**
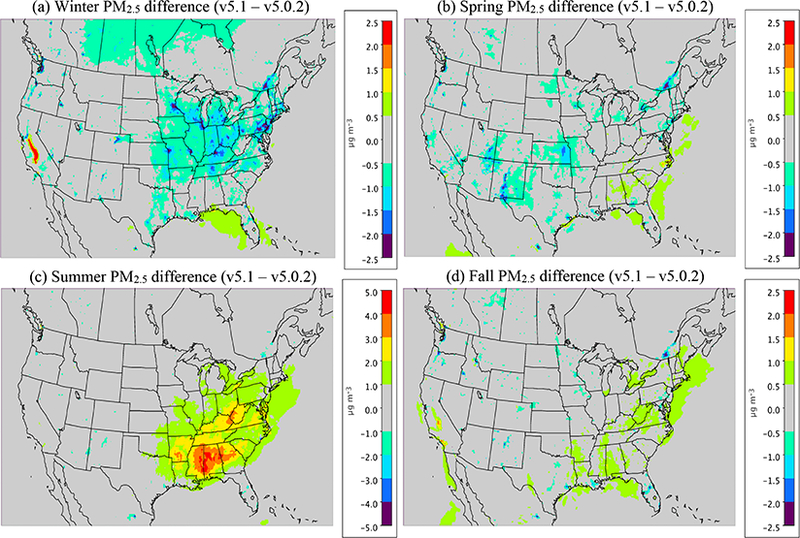
Difference in the seasonal average PM_2.5_ for **(a)** winter (DJF), **(b)** spring (MAM), **(c)** summer (JJA) and **(d)** fall (SON) between CMAQv5.0.2_Base and CMAQv5.1_Base_NEIv1 (CMAQv5.1_Base_NEIv1-CMAQv5.0.2_Base). All plots are in units of micrograms per cubic meter (μg m^−3^).

**Figure 7.**
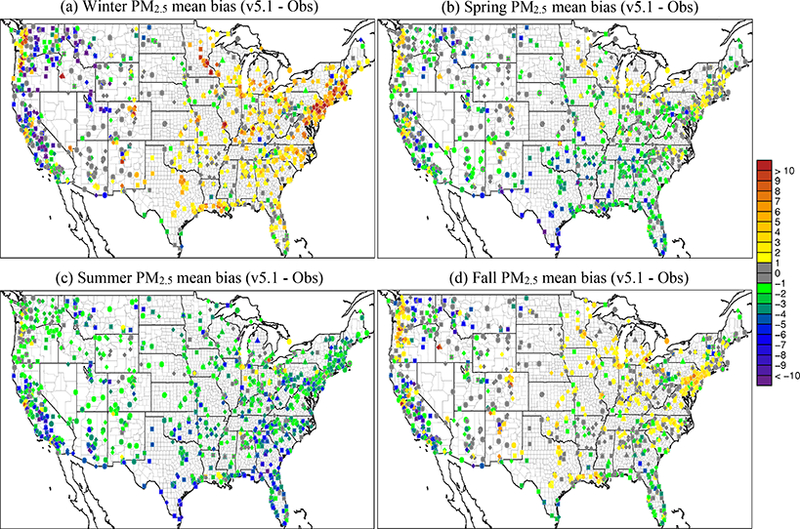
Seasonal average PM_2.5_ mean bias (μg m at IMPROVE (circles), CSN (triangles), AQS Hourly (squares) and AQS Daily (diamonds) sites for **(a)** winter (DJF), **(b)** spring (MAM), **(c)** summer (JJA) and **(d)** fail (SON) for the CMAQv5.1_Base simulation.

**Figure 8.**
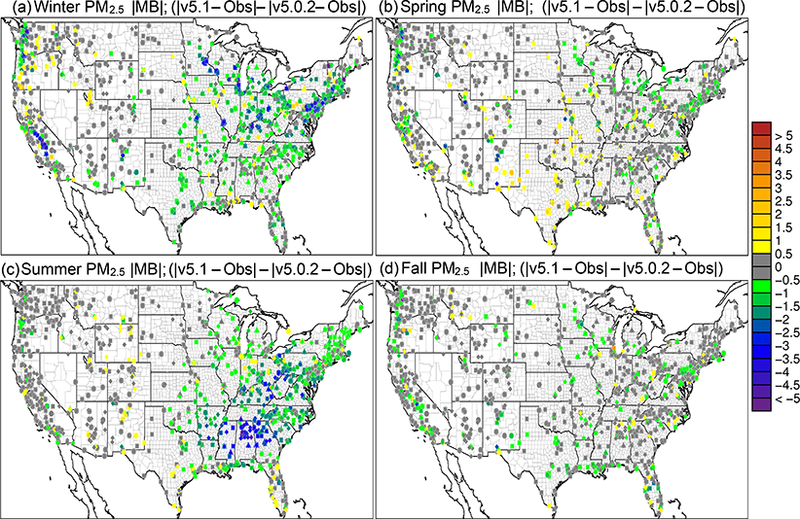
Difference in the absolute value of seasonal average PM_2.5_ mean bias for **(a)** winter (DJF), **(b)** spring (MAM), **(c)** summer (JJA) and **(d)** fall (SON) between CMAQ v5.0.2_Base and v5.1_Base_NEIvl (CMAQv5.1_Base_NEIvl-CMAQv5.0.2_Base). All plots are in units of micrograms per cubic meter (μg m^−3^). Cool colors indicate a reduction in PM_2.5_ mean bias in v5.1, while warm colors indicate an increase in PM_2.5_ mean bias v5.1.

Figure 6 shows the seasonal average difference in model-simulated PM_2.5_ between v5.0.2 and v5.1 (CMAQv5.1_Base_NEIv1-CMAQv5.0.2_Base), with cool colors indicating a decrease in PM_2.5_ in v5.1 (vs. v5.0.2) and warm colors indicating an increase in PM_2.5_. Figure 7 shows the seasonal MB for PM_2.5_ for the CMAQv5.1_Base_NEIv1 simulation, while Figure 8 shows the change in the absolute value of the seasonal MB in PM_2.5_ between the CMAQv5.0.2_Base and CMAQv5.1_Base_NEIv1 simulations. Cool colors indicate smaller PM_2.5_ MB in the CMAQv5.1_Base_NEIv1 simulation (vs. the CMAQv5.0.2_Base simulation), while warm colors indicate larger MB in the CMAQv5.1_Base_NEIv1 simulation.

During winter, v5.1 simulates lower PM_2.5_ concentrations in the eastern US and portions of central Canada compared to v5.0.2, and higher PM_2.5_ concentrations in the SJV (Fig. 6). PM_2.5_ is largely overestimated in the eastern US and underestimated in the western US (the exception being portions of the northwest) in the winter in the CMAQv5.1_Base_NEIv1 simulation (Fig. 7a). The change in MB between v5.0.2 and v5.1 is negative (reduced MB in v5.1) across the majority of the sites, with relatively large reductions (3–5 μg m^−3^) in MB in the northeast, upper Midwest (i.e., Great Lakes region) and the SJV (Fig. 8a). Figure S3 presents a histogram of the change in PM_2.5_ MB using the same data and color scale as in Fig. 8. It is clear from the histogram that there is a large percentage (72.3 %) of sites where the MB decreases in the CMAQv5.1_Base_NEIv1 simulation in the winter (Fig. S3a), demonstrating a widespread improvement in the PM_2.5_ performance for v5.1 vs. v5.0.2.

**Figure 9.**
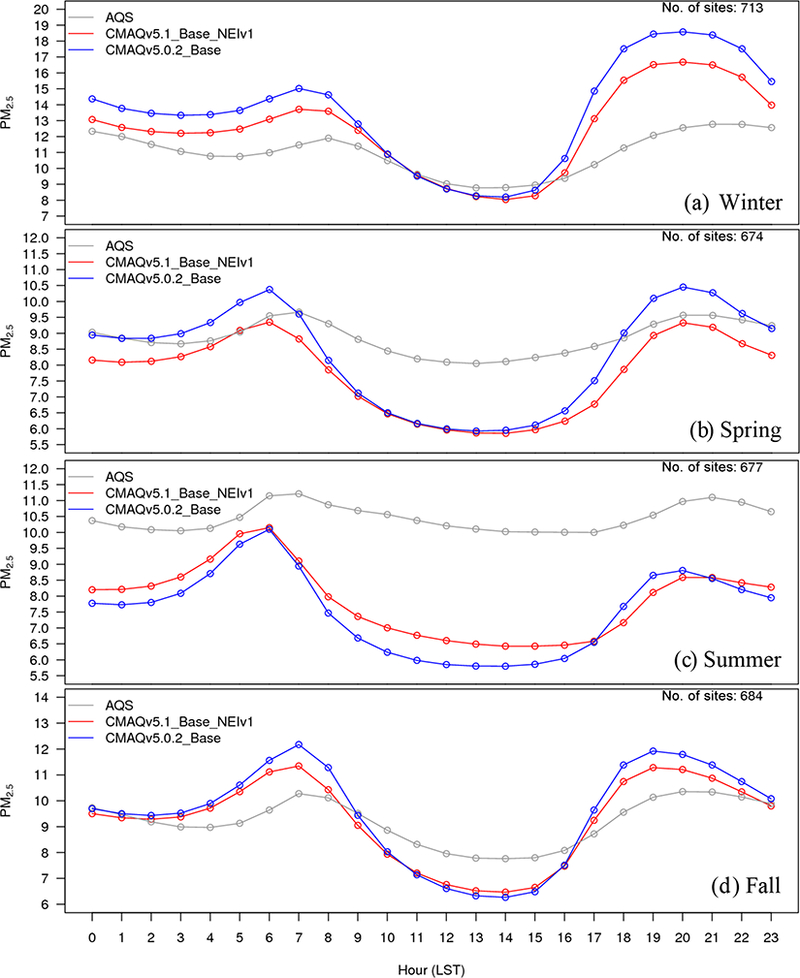
Diurnal time series of seasonal PM_2.5_ (μg m-^3^) for AQS observations (gray), CMAQv5.0.2_Base simulation (blue) and CMAQv5.1_Base_NEIv1 simulation (red) for **(a)** winter, **(b)** spring, **(c)** summer and **(d)** fall.

**Figure 10.**
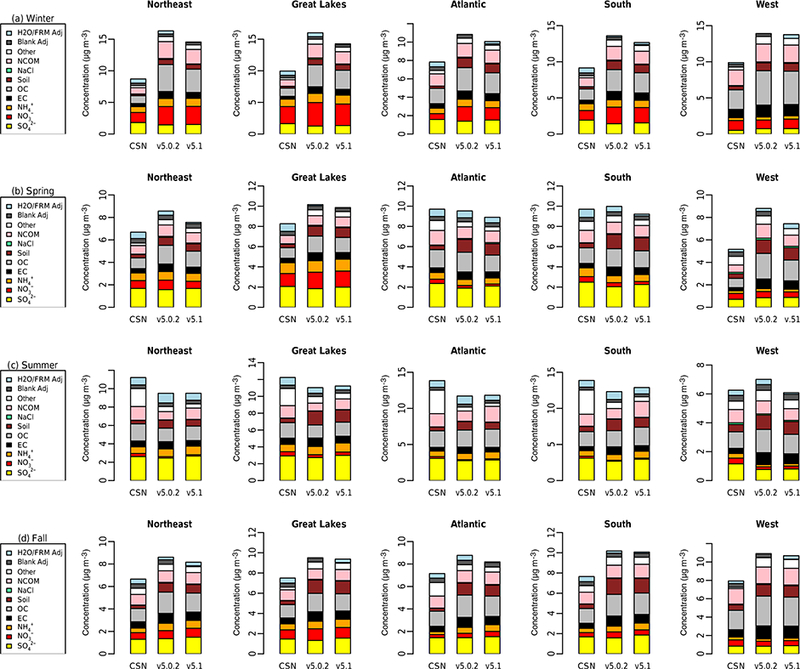
Regional and seasonal stacked bar plots of PM_2.5_ composition at the CSN sites (left), CMAQv5.0.2_Base simulation (middle) and CMAQv5.1_Base_NEIv1 simulation (right). In order from top to bottom are **(a)** winter, **(b)** spring, **(c)** summer and **(d)** fall seasons and left to right the northeast, Great Lakes, Atlantic, south and west regions. The individual PM_2.5_ components (in order from bottom to top) are ${\mathrm{SO}}_{4}^{2-}$ (yellow), ${\mathrm{NO}}_{3}^{-}$(red), ${\mathrm{NH}}_{4}^{+}$(orange), EC (black), OC (light gray), soil (brown), NaCl (green), NCOM (pink), other (white), blank adjustment (dark gray) and H_2_O / FRM adjustment (blue).

The diurnal profile of PM_2.5_ for winter (Fig. 9a) indicates a relatively large decrease in MB throughout most of the day with v5.1 vs. v5.0.2, particularly during the overnight, morning and late afternoon hours. A similar improvement is seen in the RMSE, and the correlation also improves for all hours (Fig. S5). Figure 10 shows seasonal and regional stacked bar plots of PM_2.5_ composition (SO_4_^2−^, ${\mathrm{NO}}_{3}^{-}$, ${\mathrm{NH}}_{4}^{+}$, EC, OC, soil, NaCl, NCOM and Other). Soil is based on the IMPROVE soil equation and contains both primary and secondary sources of soil (Appel et al., 2013), while Other represents the unspeciated PM mass in the inventory (see Appel et al., 2008) The five regions shown in Fig. 10 are the northeast (Maine, New Hampshire, Vermont, Massachusetts, New York, New Jersey, Maryland, Delaware, Connecticut, Rhode Island, Pennsylvania, District of Columbia, Virginia and West Virginia), Great Lakes (Ohio, Michigan, Indiana, Illinois and Wisconsin), Atlantic (North Carolina, South Carolina, Georgia and Florida), south (Kentucky, Tennessee, Mississippi, Alabama, Louisiana, Missouri, Oklahoma and Arkansas) and west (California, Oregon, Washington, Arizona, Nevada and New Mexico). These regions are derived from principle component analysis to group states with similar PM_2.5_ source regions together. For winter, the total PM_2.5_ high bias is reduced across all five regions, with most of the improvement coming from reductions in OC, non-carbon organic matter (NCOM; see Sect. 2 or 3 for definition) and Other, indicating that improvements in the representation of mixing under stable conditions helped in reducing the high bias. Still, a large bias remains for OC, which may be due in part to an overestimation of the residential wood combustion in the NEI.

For spring, the changes in PM_2.5_ are much more isolated than in winter (Fig. 6b), with the largest decreases occurring around Montreal (Canada) and portions of the Midwest and desert southwest (lack of windblown dust in v5.1 contributes to the decrease in the desert southwest). The MB for PM_2.5_ in the spring is relatively small, with most sites (75 %) reporting a MB between ±3.0 μg m^−3^, with some larger underestimations in Texas and larger overestimations in the northeast, Great Lakes and northwest (Fig. 7b). As expected with the relatively small change in modeled PM_2.5_ concentrations with v5.1 in the spring (Fig. 6b), the difference in MB between v5.0.2 and v5.1 is relatively small, with most differences in MB less than ±1.0 μg m^−3^ (Fig. 8b). Some slightly larger decreases in MB occur in the northeast and northwest, while some larger increases in MB occur in the Midwest and Texas. A little more than half (53.0 %) of the sites report an improvement in MB (Fig. S3b). The diurnal profile of PM_2.5_ for spring shows a consistent underestimation of PM_2.5_ throughout most of the day in the v5.0.2 simulation, which becomes larger in the CMAQv5.1_Base_NEIv1 simulation, with an overall decrease in PM_2.5_ in the spring (Fig. 9b). However, the RMSE is lower during the overnight, morning and afternoon hours in the CMAQv5.1_Base_NEIv1 simulation, and the correlation improves throughout most of the day as well (Fig. S5). Total PM_2.5_ MB improves in three of the five regions shown in Fig. 10, with most of the improvement coming from lower concentrations of OC and NCOM.

In the summer, PM_2.5_ is considerably higher (> 5.0 μg m^−3^) across a large portion of the eastern US in the CMAQv5.1_Base_NEIv1 simulation, particularly in Mississippi, Alabama, Georgia and portions of the Ohio Valley (Fig. 6c). The increase in PM_2.5_ is primarily due to the updates to the IEPOX-SOA chemistry in v5.1 (Fig. 2), updates to BVOC emissions in BEISv3.61 (approximately 0 μg m^−3^ increase PM_2.5_ in the southwestern US), and the ACM2 and MOL updates in WRF and CMAQ (Fig. 1), with smaller contributions from the updates in CB05e51 chemical mechanism (Fig. 5) and updates to the clouds/photolysis (Fig. 3). Despite the increase in PM_2.5_ with v5.1, PM_2.5_ still remains largely underestimated in the summer, with the largest underestimations in the southeastern US, Texas and California (Fig. 7c). However, the result of the widespread increase in PM_2.5_ with v5.1 is a similar large, widespread reduction in the MB across the eastern US, particularly in the southeast and the Ohio Valley, where reductions in MB range from 3.0 to 5.μgm^−3^ (Fig. 8c). Smaller increases in the MB (typically less than 2.μg m^−3^) occur in Florida and isolated areas in the western US. Of all the sites, 69.8 % report an improvement in MB, with a number of sites showing reductions in MB greater than 5.μg m^−3^ (Fig. S3c). PM_2.5_ is underestimated throughout the day in both the v5.0.2 and v5.1 simulations (Fig. 9c) in summer, with the underestimation improving slightly with v5.1, particularly during the afternoon and overnight hours. RMSE improves during the daytime hours with v5.1, while correlation is considerably higher with v5.1 than v5.0.2 throughout the entire day (Fig. S6). Total PM_2.5_ is underestimated in the CMAQv5.0.2_Base simulation in four of the five regions (the west region being the exception), which improves in the CMAQv5.1_Base_NEIv1 simulation (Fig. 10). The overestimation in the west region with v5.0.2 also improves with v5.1. Small increases in ${\mathrm{SO}}_{4}^{2-}$ and ${\mathrm{NH}}_{4}^{+}$ and larger increases in OC and NCOM contribute to the improvement.

For the fall, the difference in PM_2.5_ between v5.0.2 and v5.1 is again small, with the largest increases occurring in isolated portions of the eastern US and California, and the largest decreases occurring in Montreal and isolated areas in the western US (Fig. 6d). The MB pattern in the fall (Fig. 7d) is similar to the one in the spring as well (Fig. 7b), with relatively small MBs in the eastern US (±2.0 μg m^−3^) and larger MBs along the west coast (underestimated in California and overestimated in the northwest). As expected, the change in the MB between v5.0.2 and v5.1 is also relatively small in the fall, with the majority of the sites reporting a change in MB of less than ±2.0 μg m^−3^ (Fig. 8d), and 68.1 % of the sites reporting a reduction in MB (Fig. S3d). The average diurnal profile of PM_2.5_ in the fall (Fig. 9d) is similar to the spring, with improved MB with v5.1 during the overnight, morning and late afternoon or evening hours and reduced RMSE and improved correlation throughout the entire day (Fig. S8). Total PM_2.5_ is overestimated in all five regions in the fall (Fig. 10), but improves with v5.1 in all of those regions (albeit only very slightly for the south region), with decreases in EC and OC responsible for most of the improvement.

### 5.2 Ozone

**Figure 11.**
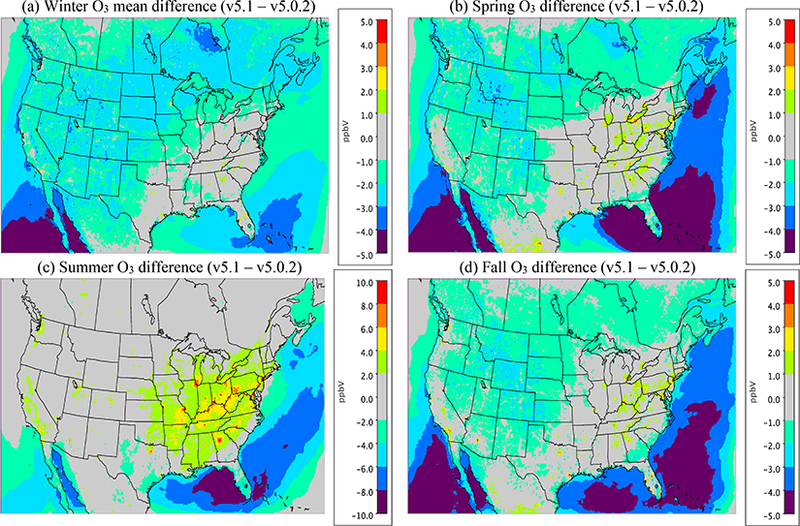
Difference in the monthly average hourly O_3_ (ppbv) for winter (DJF; top) left), spring (MAM; top right), summer (JJA; bottom left) and fall (SON; bottom right) between CMAQ v5.0.2_Base and v5.1_Base_NEIv1 (CMAQv5.1_Base_NEIv1-CMAQv5.0.2_Base). Note that the scales between each plot may vary.

**Figure 12.**
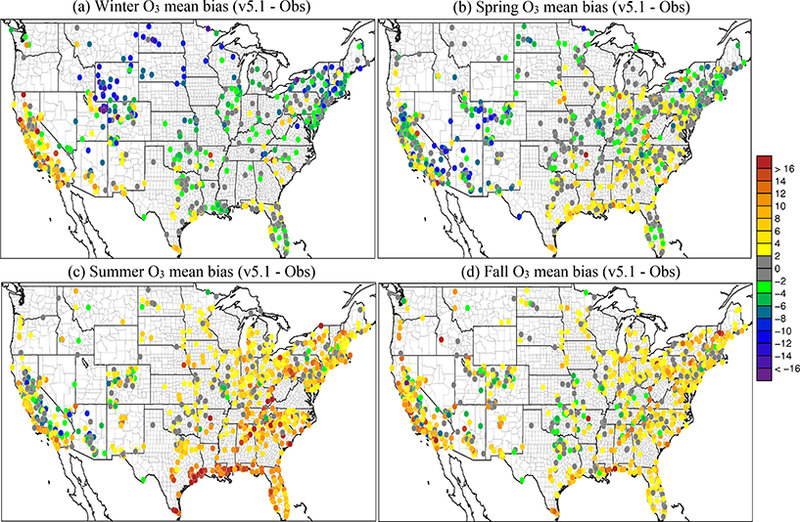
Seasonal average hourly O_3_ (ppbv) mean bias at AQS sites for **(a)** winter (DJF), **(b)** spring (MAM), **(c)** summer (JJA) and **(d)** fall (SON) for the CMAQv5.1_Base_NEIv1 simulation.

**Figure 13.**
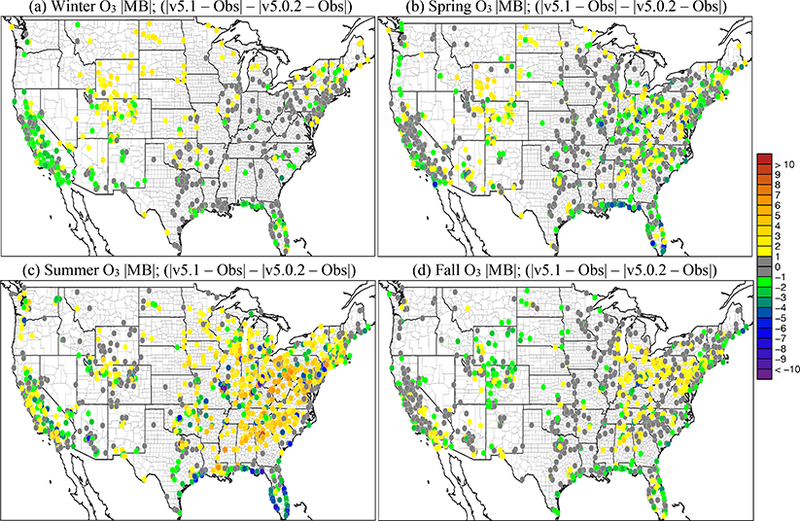
Difference in the absolute value of monthly. average O_3_ (ppbv) mean bias for **(a)** winter (DJF), **(b)** spring (MAM), **(c)** summer (JJA) and **(d)** fall (SON) between CMAQ v5.0.2_Base and v5.1_Base_NEIv1 (CMAQv5.1_Base_NEIv1-CMAQv5.0.2_Base). Cool colors indicate a reduction in O_3_ mean bias in v5.1, while warm colors indicate an increase in O_3_ mean bias v5.1.

**Figure 14.**
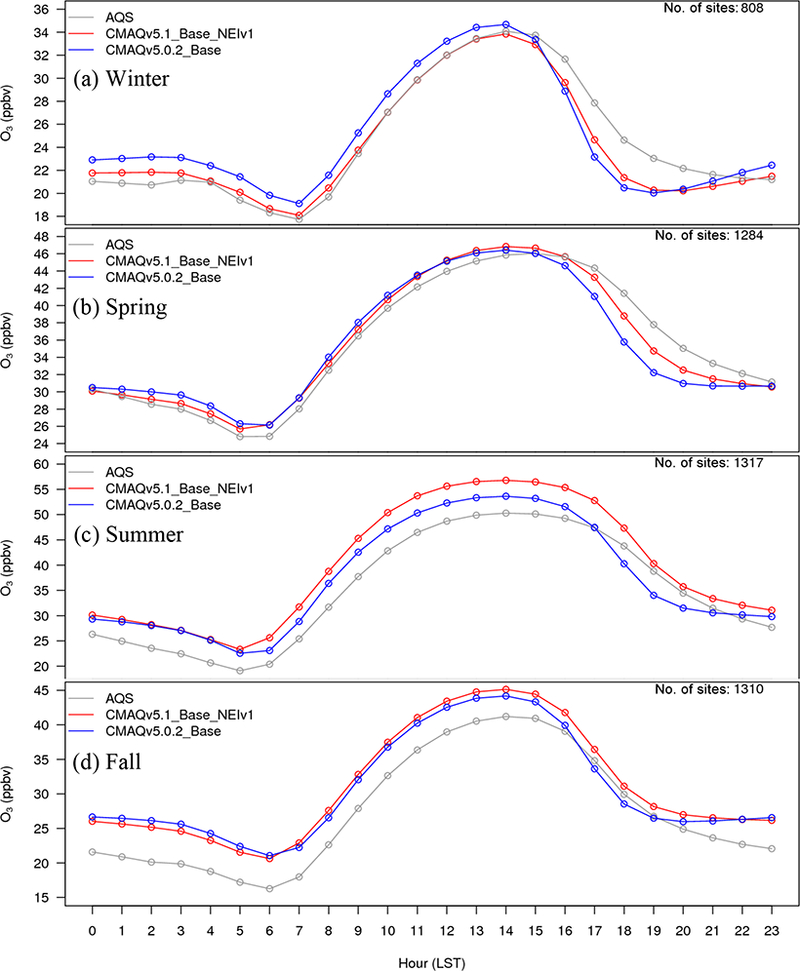
Diurnal time series of seasonal O_3_ (ppbv) for AQS observations (gray), CMAQv5.0.2_Base simulation (blue) and CMAQv5.1_Base_NEIv1 simulation (red) for **(a)** winter, **(b)** spring, **(c)** summer and **(d)** fall

**Figure 15.**
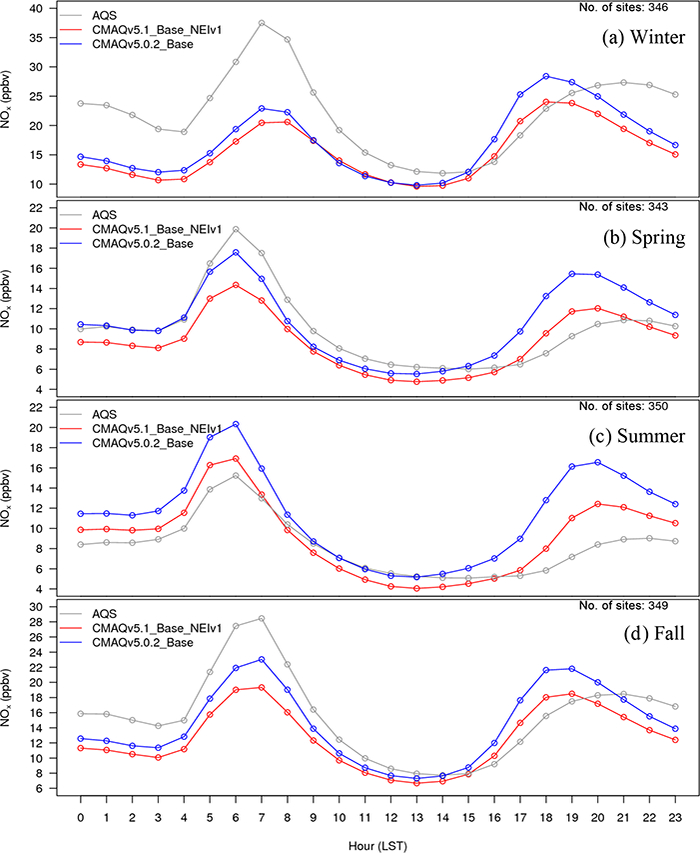
Diurnal time series of seasonal NO_*x*_ (ppbv) for AQS observations (gray), CMAQv5.0.2_Base simulation (blue) and CMAQv5.1_Base_NEIv1 simulation (red) for **(a)** winter, **(b)** spring, **(c)** summer and **(d)** fall.

For the winter, O_3_ widely decreases in the CMAQv5.1_Base_NEIv1 simulation vs. the CMAQv5.0.2_Base simulation across the western US, with the seasonal average decreases ranging between 1.0 and 3.0 ppbv, and several areas where decreases exceed 3.0 ppbv, primarily over the oceans (Fig. 11a). In the eastern US, the change in O_3_ is relatively small and isolated. Ozone is underestimated at most sites across the northern portion of the US, with the largest underestimations occurring in Colorado, Wyoming and Utah. Despite the decreases in O_3_ with v5.1, O_3_ is still overestimated in the southwestern US and California (Fig. 12a). There is a widespread reduction in the O_3_ MB in California and increased MB in the upper Midwest with v5.1, while across the rest of the domain the change in MB is relatively small (Fig. 13a). The majority of the change in O_3_ falls between ±5.0 ppbv, with 56.5 % reporting a reduction in MB (Fig. S8a). The average diurnal profile of O_3_ in the winter (Fig. 14a) shows slightly lower mixing ratios during most of the day with v5.1, the exception being the late afternoon and early evening hours, when mixing ratios are slightly higher. The result is reduced MB and RMSE, and higher correlation throughout the day with v5.1 vs. v5.0.2 (Fig. S9). The NO_*x*_ diurnal profile also generally improves throughout the day in winter (Fig. 15a), with decreased MB and RMSE in the afternoon or early evening and increased correlation throughout the day (Fig. S11).

The pattern of change in O_3_ between v5.0.2 and v5.1 in spring is similar to winter, with lower O_3_ mixing ratios in the western US and higher mixing ratios in the eastern US in v5.1 compared to v5.0.2 (Fig. 11b). Decreases in O_3_ mixing ratios in the western US in v5.1 range from roughly 1.0 to 3.0 ppbv (similar to winter), while in the eastern US the increases generally range from 1.0 to 2.0 ppbv, with isolated areas of larger increases. The MB of O_3_ for the v5.1 simulation primarily ranges from slightly overestimated to slightly underestimated across most the sites, with larger overestimations along the Gulf Coast and larger underestimations in the western US (Fig. 12b). The change in MB between v5.0.2 and v5.1 shows mixed results (Fig. 13b), with slight increases and decreases across much of the eastern US and a relatively large increase in MB in the Midwest (i.e., Colorado and Wyoming). The MB mostly improved across the Gulf Coast and in California due to reduced O_3_ mixing ratios from the new marine halogen chemistry and enhanced O_3_ deposition to ocean surfaces. Half of the sites reported a reduction in MB (Fig. S8b) with v5.1. The diurnal profile of O_3_ for spring (Fig. 14b) shows a relatively large increase in mixing ratios in the late afternoon and evening (04:00 to 22:00 LST), resulting in a large improvement in MB during that time (Fig. S11). Similar improvements are noted in RMSE and correlation in the after-noon and evening hours. The NO_*x*_ diurnal profile also shows a large decrease in the late afternoon and early evening mixing ratios (Fig. 15b), with a large decrease in both MB and RMSE during that time, and improved correlation throughout the day (Fig. S12).

For the summer, the pattern of change in O_3_ is markedly different from the winter and spring, with a large widespread increase in O_3_ mixing ratios across the eastern US and decreases in the Gulf of Mexico, southern Florida and over the eastern Atlantic Ocean (Fig. 11c). Increases in O_3_ in the eastern US range from 2.0 to 10.0 ppbv, with isolated areas of larger increases in the major urban areas (e.g., Chicago, Illinois, and Atlanta, Georgia) that can be largely attributed to the updates to the ACM2 and the MOL calculation in WRF and CMAQ (Fig. 2b) as well as increased photolysis in v5.1 vs. v5.0.2 (Fig. 1). Smaller increases in O_3_ occur in the western US, particularly southern California and the SJV. Large decreases in O_3_ over the oceans are likely the result of the inclusion of the marine halogen chemistry in v5.1, with some decreases exceeding 10.0 ppbv. The MB of O_3_ for the v5.1 simulation shows widespread overestimations in the eastern US, particularly along the Gulf of Mexico, while in the western US the MB is mixed, with the largest overestimations occurring along the California coast (Fig. 12c).

As expected, the consequence of the widespread increase in O_3_ in the eastern US in v5.1 is a corresponding widespread increase in the MB compared to v5.0.2, particularly in the mid-Atlantic and southeast (Fig. 13c). Ozone MB decreases along the coast of Florida and along the Gulf of Mexico, the result of decreased O_3_ over the water. The change in MB in the western US is mixed, with some areas showing improved MB (e.g., SJV), while others show increased MB (e.g., southern California). The diurnal profiles of O_3_ show that mixing ratios increase throughout most of the day in v5.1 (the exception being 00:00–05:00 LST) (Fig. 14c), resulting in increased MB and RMSE throughout the morning and early afternoon hours (Fig. S13). However, RMSE decreases substantially during the late afternoon and overnight hours, and the correlation improves throughout the entire day. The NO_*x*_ concentrations are lower throughout the day with v5.1 compared to v5.0.2 (Fig. 15c), which results in large improvements in the MB in the morning and afternoon periods and slightly increased MB in the middle of the day, while RMSE and correlation improve throughout the day (Fig. S14).

For the fall, the pattern of change in O_3_ for v5.1 vs. v5.0.2 is nearly identical to spring (Fig. 11d), with widespread decreases in O_3_ in the western US (possibly due to reduced cloud mixing and entrainment from the free troposphere) and mostly small increases in O_3_ in the eastern US, with the exception of larger increases in several of the major urban areas (e.g., St. Louis, Missouri and Atlanta, Georgia). The changes are generally small, between ±2.0 ppbv, with isolated areas of larger increases or decreases. Ozone is also lower over the Pacific and Atlantic oceans and the Gulf of Mexico. While the change in O_3_ between v5.0.2 and v5.1 is very similar to the spring, the MB pattern for v5.1 is not. Unlike the spring, where O_3_ was underestimated in many areas, in the fall O_3_ is overestimated for almost all the sites (Fig. 12d). Sites in the Midwest have the lowest overall MB, while the east and west coasts show large overestimations of O_3_. The increased O_3_ in the eastern US with v5.1 results in generally higher MB compared to v5.0.2, while in the western US the result is slightly lower MB on average, the exception being southern California (Fig. 13d). As was the case in the spring, slightly less than half the sites (48.4 %) report a reduction in MB, with the majority of the change falling between ±5.0 ppbv (Fig. S8d). The diurnal profile of O_3_ in the fall shows increased mixing ratios with v5.1 during the daytime hours and slightly decreased mixing ratios overnight (Fig. 14d), resulting in increased MB during the daytime and lower MB overnight. However, the RMSE is reduced and the correlation is higher throughout the day (Fig. S15). Similar to the other seasons, the diurnal profile of NO_*x*_ in the fall shows lower mixing ratios throughout the day (Fig. 15d), particularly in the early morning and late afternoon hours, resulting in higher MB in the morning and lower MB in the afternoon, while RMSE is reduced and correlation is higher throughout the entire day with v5.1 (Fig. S16).

### 5.3 ANs, PNs and NO_*y*_

Previous studies have shown that CMAQ can significantly overestimate NO_*y*_ mixing ratios (e.g., Anderson et al., 2014). To help address the NO_*y*_ overestimation in CMAQ, updates were made to the atmospheric chemistry in v5.1 pertaining to the formation and cycling of alkyl nitrates (ANs), peroxy nitrates (PNs) and NO_*y*_ in the model (Sect. 2.4.1). Overall, monthly average hourly NO_*y*_ mixing ratios at AQS sites decreased between approximately 13 % (January) and 21 % (July) in the CMAQv5.1_Base_NEIv1 simulation vs. the CMAQv5.0.2_Base simulation. The result is a slight decrease in the normalized mean error (NME) in January from 70 % (v5.0.2) to 61 % (v5.1), but a much larger decrease in NME in July from 151 % (v5.0.2) to 101 % (v5.1). Mixing ratios of ANs and PNs are not routinely measured; however, the DISCOVER-AQ campaign (https://www.nasa.gov/ mission_pages/discover-aq/) that took place over the Baltimore, Maryland and Washington DC area in July 2011 provides aircraft measurements of PNs and ANs, along with NO_2_, NO_*y*_, HNO_3_ and O_3_. The National Oceanic and Atmospheric Administration (NOAA) P3B aircraft performed measurement flights on a number of days during the DISCOVER-AQ campaign. Those flights included vertical spirals over several locations, one of which was Edgewood, MD (39.41° N, 76.30° W; 11 m above sea level), a site that often measures very high O_3_, and in recent years has measured some of the highest O_3_ in the eastern US.

**Figure 16.**
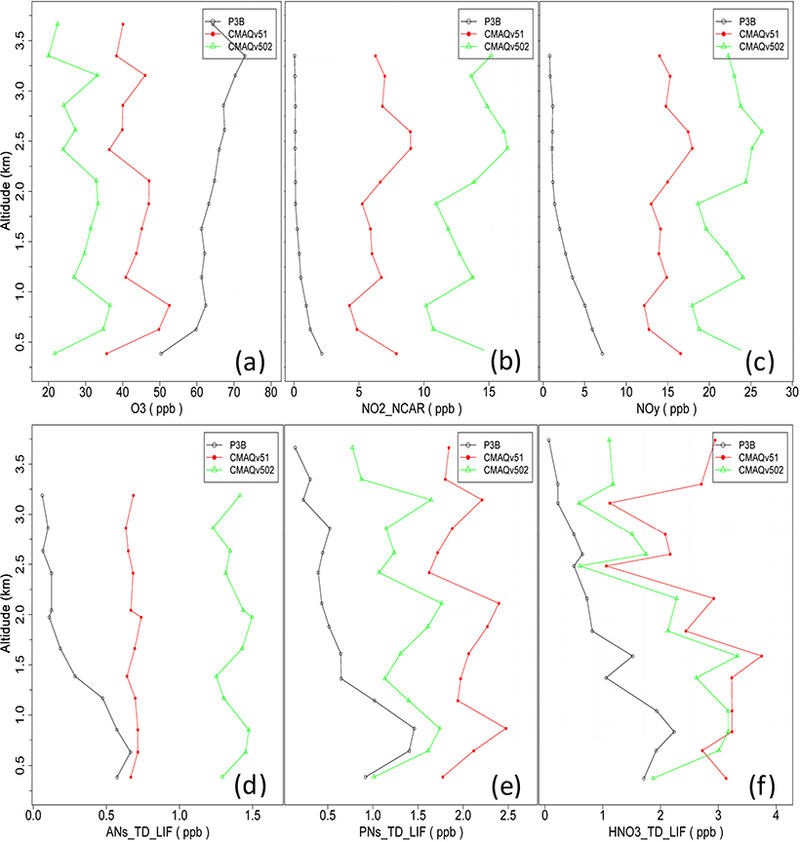
Observed (black) and CMAQ-simulated vertical profiles of **(a)** O_3_, **(b)** NO_2_, **(c)** NO_*y*_, **(d)** alkyl nitrates (ANs), **(e)** peroxy nitrates (PNs) and **(f)** HNO_3_ for the Edgewood site in Baltimore, MD, on 5 July 2011. CMAQv502_Base simulation profiles are shown in green and CMAQv51_Base_NEIv1 simulation profiles are shown in red. Altitude (km) is given on the *y* axis, while mixing ratio (ppbv) is given on the x axis.

Figure 16 shows vertical profiles of observed and CMAQ (v5.0.2 and v5.1)-simulated O_3_, NO_2_, NO_*y*_, ANs, PNs and nitric acid (HNO_3_) for the Edgewood site on 5 July 2011. Several spirals were performed over the Edgewood site that day, roughly taking place in the late morning, early afternoon and late afternoon, so the profiles shown represent an average profile throughout the day. While O_3_ is underestimated throughout the PBL by both versions of the model on that day, the underestimation improves significantly in the v5.1 simulation. NO_2_ and NO_*y*_ are overestimated throughout the PBL by both versions of the model, but again, the overestimation is greatly improved in the v5.1 simulation. The PNs, ANs and HNO_3_ show mixed results, with the AN performance improving, the PN performance degrading and the HNO3 performance relatively unchanged with v5.1. Note that there has been an update in the recommended PAN formation and degradation equilibrium constant (http://iupac.pole-ether.fr) which lowers the predicted PAN concentrations in CMAQ and is currently being examined for its impact on other species. On this particular day, v5.1 generally shows a large improvement in performance over v5.0.2; however, the results on other days may be different, but it does highlight the large change in NO_*y*_ mixing ratios that can be expected with v5.1.

## 6 Modeled response to emission changes

One of the primary applications of air quality models is to determine the impact that changes (e.g., reductions from abatement strategies) in emissions have on ambient air quality. Examples of this type of application include federal rules and state implementation plans (SIPs) which aim to reduce emissions (through regulations) in order to meet mandated air quality standards. In this type of application, the air quality model is run using both baseline (often current year) and future year emissions (when emissions are typically lower due to state and national regulatory efforts) and then the change in criteria pollutant (e.g., O_3_ and PM_2.5_) concentrations between the two simulations is quantified in order to assess the impact (benefit) that emission reductions will have on future ambient air quality. As such, it is important to establish the ability of the model to accurately simulate the future ambient air quality given a known change in emissions, which here is referred to as the model responsiveness (to emission changes).

Some previous analyses comparing observed changes in ambient air quality (over periods witnessing large reductions in emissions) to CMAQ-estimated changes in ambient air quality (with estimated reductions in emissions) during the same period have shown that the model tends to underestimate the observed change in ambient O_3_, suggesting the model may be under responsive to the emission reductions impacting O_3_ (Gilliland et al., 2008; Foley et al., 2015). The over- or under responsiveness of the model to emission projections can have implications in the planning process for determining the extent to which emissions must be reduced in order to meet future air quality standards. In the following sections, we examine the model responsiveness to emission reductions in CMAQ v5.0.2 and v5.1 by computing the ratio of maximum daily 8 h average (MDA8) O_3_ mixing ratios and total PM_2.5_ (and select PM_2.5_ component species) between simulations using the base emission inventories and those employing 50 % reductions in NO_*x*_, VOC and SO_*x*_ emissions in order to estimate a model responsiveness to the emission reductions for each version of the model. The model responsiveness for v5.1 is then compared to that of v5.0.2 to determine whether the model responsiveness increased, decreased or was unchanged in the new version of the model.

### 6.1 O_3_

**Figure 17.**
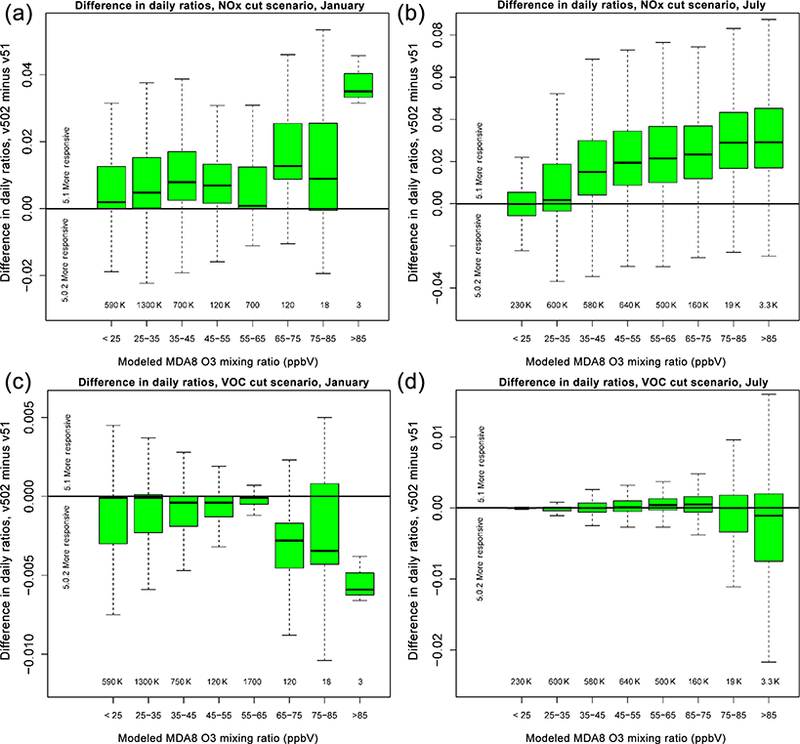
Difference in MDA8 O_3_ daily ratios (cut scenario / base) for CMAQv5.0.2 and v5.1 (v5.0.2-v5.1) for a 50 % cut in anthropogenic NO_*x*_ **(a-b)** and VOC **(c-d)** for January **(a, c)** and July **(b, d)** binned by the modeled MDA8 O_3_ mixing ratio (ppbv). Values greater than 1 indicate v5.1 is more responsive than v5.0.2 to the emissions cut, while values less than 1 indicate v5.0.2 is more responsive. Given above the x axis is the number of model grid cells in each bin.

Figure 17 shows the difference in the ratio (emission cut simulation / base simulation) of MDA8 O_3_ for the 50 % cut in anthropogenic NO_*x*_ and VOC scenarios, binned by model MDA8 O_3_ mixing ratio. Values greater than zero indicate v5.1 is more responsive to the NO_*x*_ or VOC cut than v5.0.2, while values less than zero indicate v5.1 is less responsive than v5.0.2. For both January and July, the median difference in ratio values for all bins for the 50 % NO_*x*_ cut scenario are greater than zero, indicating that v5.1 is more responsive than v5.0.2 to the cut in NO_*x*_. For the 50 % cut in VOC emissions, the difference in the ratio values is mixed across the 2 months and the different bins. For January, all of the bins indicate that v5.0.2 is more responsive than v5.1 to the 50 % VOC cut, with the greatest difference occurring for MDA8 O_3_ mixing ratios greater than 65 ppbv. For July, v5.1 is slightly more responsive to the VOC cut for MDA8 O_3_ mixing ratios less than 75 ppbv and less responsive for MDA8 O_3_ mixing ratios greater than 85 ppbv.

### 6.2 PM_2.5_

**Figure 18.**
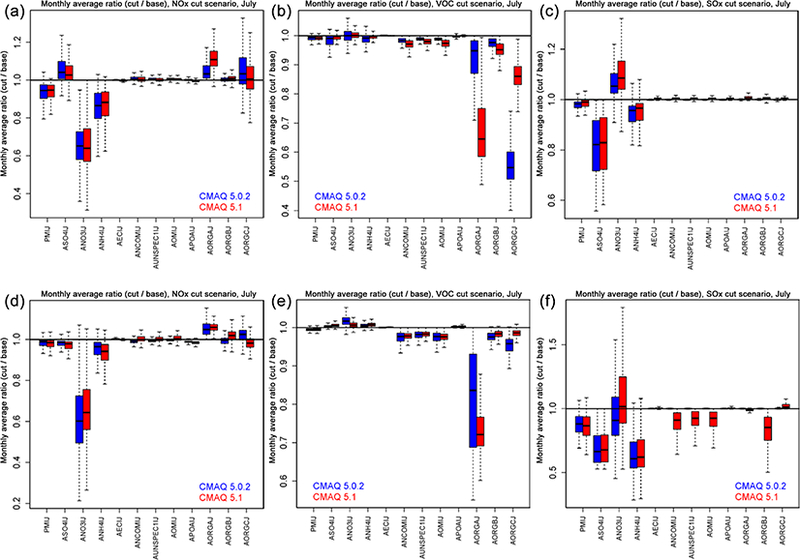
Box plots of monthly average ratio values (cut / base) of PMIJ (total PM_2.5_), ASO4IJ, ANO3IJ, ANH4IJ, AECIJ, ANCOMIJ, AUNSPECIJ, AOMIJ, APOAIJ, AORGAJ, AORGBJ and AORGCJ for v5.0.2 (blue) and v5.1 (red) for a 50% cut in anthropogenic NO_*x*_ **(a, d)**, VOC **(b, e)** and SO_*x*_ **(c, f)** for January **(a-c)** and July **(d-f)**.

Figure 18 shows the difference in the ratio (emission cut simulation / base simulation) of PM_2.5_ and select PM_2.5_ component species between v5.0.2 and v5.1 for January and July for a 50 % cut in anthropogenic emissions of NO_*x*_, VOC and SO_*x*_. For January, the overall response of modeled PM_2.5_ (PMU) to a 50 % reduction in NO_*x*_ is primarily driven by a decrease in nitrate and its associated ammonium. CMAQv5.1 PM_2.5_ is slightly less responsive to NO_*x*_ reductions compared to v5.0.2, but is still overall quite similar. The VOC cut shows greater response with v5.1 than v5.0.2 in January in ANCOMIJ (non-carbon organic matter attached to primary organic carbon; Simon and Bhave, 2012), AUNSPECIJ (unspeciated PM), AOMIJ (all organic matter), AORGAJ (SOA from anthropogenic VOCs), AORGBJ (SOA from biogenic VOCs) and total PM_2.5_ (see Sects. 2 and 3). Note that the letters I and J after the species name indicate which CMAQ modal distributions are being included in the total species mass, with I indicating the Aitken mode and J indicating the accumulation mode. Since NCOMIJ is nonvolatile, its change reflects how reducing VOCs changes oxidants such as OH. In general, the model PM_2.5_ is not very sensitive to VOC cuts in January. And finally, for the 50 % SO_*x*_ cut scenario PM_2.5_ is only slightly less responsive with v5.1, with all the species being similarly responsive to the SO_*x*_ cut using v5.1 compared to v5.0.2.

For July, the NO_*x*_ cut scenario with v5.1 shows greater responsiveness for the ASO4IJ (sulfate), ANH4U (nitrate), AECIJ (elemental carbon), APOAIJ (primary organic aerosol) and AORGCJ (SOA from glyoxal and methylgly-oxal processing in clouds) species and total PM_2.5_ vs. v5.0.2. For the VOC cut scenario, the AORGAJ species show increased responsiveness with v5.1. CMAQv5.1 alkane SOA is not dependent on NO_*x*_ levels or HO_2_ : NO ratios, so the decrease in VOC precursors have a more direct effect than for the aromatic systems (the only AORGAJ in v5.0.2), where decreasing the VOC precursors can also modify the HO_2_ :NO ratio and thus yields. CMAQv5.1 PMIJ becomes slightly more responsive to SO_*x*_ cut as a result of an increased sensitivity of biogenic SOA to sulfur containing compounds. This link results from the IEPOX acid-catalyzed SOA in the model which has been shown to be correlated with sulfate (Pye et al., 2016).

## 7 Summary

A new version of the CMAQ model (v5.1) containing numerous scientific updates has been released and evaluated in terms of the change in performance against the previous version of the model (v5.0.2), performance compared to observations, and response to changes in inputs (i.e., emissions). Specifically, updates were made to the ACM2 scheme in both WRF and CMAQ to improve the vertical mixing in both models, along with updates to the MOL calculation, which also directly impacted the vertical mixing in the WRF-CMAQ system. The overall net effect of these updates was to increase the ventilation in the model, particularly during the transition periods (morning and evening), which in turn reduced the concentration of primary emitted species (e.g., NO_*x*_ and OC) and consequently increased simulated O_3_ (a result of reduced NO_*x*_ titration) and decreased PM_2.5_ concentrations due to greater dilution. Several new SOA formation pathways and species were added to v5.1, resulting in increased SOA, particularly in the southeastern US, and improved PM_2.5_ performance in the summer, as PM_2.5_ is generally underestimated by CMAQ during the summer in the US.

The in-line photolysis model within CMAQ was updated in v5.1. Cloud cover for the photolysis model in v5.0.2 used a single cloud deck with a constant cloud fraction and water droplet mixing ratio. In v5.1, multiple cloud decks with variable cloud fractions and multiple types of water condensates are used in the photolysis model to be more consistent with the WRF meteorological model and the CMAQ cloud model. The net effect of this change was to decrease the amount of subgrid clouds in the photolysis calculation in v5.1, which in turn results in higher photolysis rates and thus higher predicted O_3_ mixing ratios on average. In addition to the change to the photolysis model, the refractive indices for aerosol species are now both wavelength-and composition- dependent. Changes in aerosol scattering and extinction also introduce options for how to calculate their optical properties and allow the user to specify which aerosol mixing model and method to use to solve the Mie scattering theory. The atmospheric chemistry in the model has also been updated from CB05TUCL to CB05e51 in v5.1, which includes, among other things, updates to the NO_*y*_ reactions, additional isoprene extensions, explicit representation of several HAPs and a simple parameterization of the effects of halogens on O_3_ in marine environments. The net effect of going from CB05TUCL to CB05e51 was to increase O_3_ in the winter and summer, while increasing PM_2.5_ slightly in the winter and increasing or decreasing PM_2.5_ slightly in the summer.

Overall, the scientific updates in v5.1 resulted in improved model performance for PM_2.5_ in the winter and summer and a very small overall change in performance for the spring and fall. Wintertime PM_2.5_ concentrations are considerably lower with v5.1 vs. v5.0.2, a season when PM_2.5_ is typically overestimated by CMAQ over the US. Conversely, during the summer when PM_2.5_ is largely underestimated by CMAQ over the US, PM_2.5_ concentrations are typically higher with v5.1 vs. v5.0.2, particularly in the southeastern US. The change in O_3_ mixing ratios in v5.1 resulted in mixed improvement in MB, both spatially and temporally, with the summer showing the largest increase in MB. However, RMSE largely improved regardless of season and showed a larger improvement spatially across the sites than MB, and the correlation was almost always higher with v5.1. Comparisons of vertical profiles of several species taken over Edgewood, MD, during the DISCOVER-AQ campaign showed improved performance with v5.1 throughout the PBL for O_3_, NO_2_, NO_*y*_, ANs and CO, with the PNs being the only species to show degraded performance on that day.

The response of the model to changes in emission inputs was examined by comparing the ratio of the base v5.0.2 and v5.1 simulations to sensitivity simulations with 50% cuts each to anthropogenic NO_*x*_, VOC and SO_*x*_ emissions. CMAQv5.1-simulated MDA8 O_3_ exhibited more responsiveness (greater reduction) to the 50 % NO_*x*_ cut in January and July than v5.0.2, which is considered an improvement as previous studies suggested CMAQ O_3_ to be underresponsive to large changes in emissions. The responsiveness of PM_2.5_ to the emission cuts is more complicated than for O_3_ since there are many more species comprising PM_2.5_ and some of those have greater or smaller response with v5.1. However, the new pathways of formation for several PM_2.5_ components in v5.1 generally result in greater responsiveness in v5.1 compared to v5.0.2 for the various emission cut scenarios.

Finally, a number of important science updates are in development and will be available in the next release of CMAQ (v5.2), which improve upon the existing science in the model. These updates include a new version of the wind blown dust treatment (Foroutan et al., 2017), the carbon bond 6 (CB6) chemical mechanism (Ramboll Environ, 2016), enhancements to the calculation of semi-volatile primary organic aerosol (POA) and SOA from combustion sources in CMAQ (Pye et al., 2016), and additional updates to the calculation of clouds. In addition to the model updates, a number of instrumented versions of the model (e.g., decoupled direct method, sulfur tracking) will also be released with v5.2. These updates represent potentially significant improvements over the current options in v5.1 (specifically the updated windblown dust treatment) and therefore are being made available to the community more quickly than they might have in the past.

## Supplementary Material

Supp

## Figures and Tables

**Table 1.** New and revised SOA species in the CMAQv5.1 AERO6 mechanism.

Aerosol species	Change since v5.0.2	Applicable mechanism	Description of modification
AH3OP	added	all	Hydronium ion (predicted by ISORROPIA for I + J modes); used for IEPOX uptake
APAH1,2	added	cb05e51, saprc07tb, saprc07tc, saprc07tic, racm	Naphthalene aerosol from RO_2_ + NO reactions
APAH3	added	cb05e51, saprc07tb, saprc07tc, saprc07tic, racm	Naphthalene aerosol from RO_2_ + HO_2_ reactions
AISO1,2	updated	cb05e51, saprc07tb, saprc07tc*, racm	Aerosol from isoprene reactions NO_3_ added to existing OH (all yields follow the OH pathway)
AISO3	updated	cb05e51, saprc07tb, saprc07tc*, racm	Aerosol from reactive uptake of IEPOX on aqueous aerosol particles. Specifically intended to be the sum of 2-methyltetrols and IEPOX- derived organosulfates
AALK1,2	added	cb05e51, saprc07tb, saprc07tc, saprc07tic, racm	Alkane aerosol
AALK	removed	all	Deprecated alkane aerosol

* AERO6i does not include SOA from isoprene + NO_3_ in AISO1,2 (it is included in AISOPNNJ). AERO6i does not include IEPOX SOA in AISO3 (it is included in AITETJ, AIEOSJ, AIDIMJ, etc.). AISO3 is approximately zero in AERO6i.

**Table 2.** Description of the CMAQ model simulations utilized.

CMAQ simulation name	CMAQ version	WRF version	NEI version	Photolysis scheme	Chemical mechanism	Simulation period (all 2011)
CMAQv5.0.2_Base	v5.0.2	v3.4	v1	v5.0.2	CB05TULC	Annual
CMAQv5.0.2_WRFv3.7	v5.0.2	v3.7	v1	v5.0.2	CB05TUCL	January and July
CMAQv5.1_Base_NEIv1	v5.1	v3.7	v1	v5.1	CB05e51	Annual
CMAQv5.1_Base_NEIv2	v5.1	v3.7	v2	v5.1	CB05e51	Annual
CMAQv5.1_Retrophot	v5.1	v3.7	v2	v5.0.2	CB05e51	January and July
CMAQv5.1_TUCL	v5.1	v3.7	v2	v5.1	CB05e51	January and July
