# Supplementary material for: Description and evaluation of the Community Multiscale Air Quality (CMAQ) modeling system version 5.1
Source: Geosci Model Dev. Author manuscript; Available in PMC 2018 Aug 22. (PMC6104654; doi:10.5194/gmd-10-1703-2017)
Supplement: Supp [file NIHMS982687-supplement-Supp.pdf]

Supplement of Geosci. Model Dev., 10, 1703–1732, 2017
http://www.geosci-model-dev.net/10/1703/2017/
doi:10.5194/gmd-10-1703-2017-supplement
© Author(s) 2017. CC Attribution 3.0 License.

Geoscientific Model Development
Open Access

Supplement of

Description and evaluation of the Community Multiscale Air Quality (CMAQ) modeling system version 5.1

K. Wyat Appel et al.

Correspondence to: K. Wyat Appel (appel.wyat@epa.gov)

The copyright of individual parts of the supplement might differ from the CC-BY 3.0 licence.

Section S.1: Use of satellite measured cloud albedo to evaluate cloud parameterizations in CMAQ and WRF

Cloud albedo is a measure of the solar radiation that is reflected by a cloud and is one of the Imager products available from NASA's Geostationary Operational Environmental Satellite (GOES). There were 301 hours with available GOES cloud albedo data for the modeling domain during July 1 – July 31, 2011.

Although the WRF and CMAQ systems do not use cloud albedo directly in their cloud parameterizations, this variable provides a useful model diagnostic for identifying areas where the models are over or under-predicting the degree of cloudiness over a region.

WRF cloud albedo is calculated as:

$$CLDALB\_WRF = (SWUPT - SWUPTC)/SWDNT*100\% \quad (1)$$

where SWUPT is the upwelling shortwave flux, SWUPTC is the upwelling clear sky shortwave flux and SWDNT is the downwelling shortwave flux. All three fluxes are instantaneous and at the top of the model.

Cloud albedo within the CMAQv5.1 and CMAQv5.0.2 photolysis module is calculated as:

$$CLDALB\_NEW = \pi*(REFLECTION - CLR\_REFLECTION) \quad (2)$$

where REFLECTION is the shortwave reflection, CLR_REFLECTION is the clear sky shortwave reflection, both are instantaneous and at the top of the model.

Table S1 provides categorical evaluation metrics for model predicted clouds based on the CMAQv5.02 photolysis module, the CMAQv5.1 photolysis module and WRFv3.7 cloud parameterization compared to GOES satellite data. Cloud albedo is used to determine the presence of clouds in each grid cell for each hour. A cloud albedo > 5% (modeled or observed) is used as the threshold to indicate cloudy conditions. The Agreement Index (Biazer et al., 2014) is the fraction of correct model predictions (true clear skies OR true cloudy conditions) out of all grid cell/hours with available GOES data. Model over-predictions (3rd row) are the number of grid cell/hours where the model predicted cloudy conditions but the GOES product showed clear skies. Model under-predictions indicate the model predicted clear skies when the GOES product show cloudy conditions. Each metric is calculated separately for all hourly data available at land grid cells (N=20,440,007) and all hourly data available at ocean grid cells (N= 9,994,404).

The relative frequency of model over-prediction of cloudy conditions over land decreased from 0.20 in CMAQv5.02 to 0.06 in CMAQv5.1 and is now consistent with the WRF evaluation over land. However, CMAQv5.1 over-predicts clouds over the ocean to a greater extent than WRF3.7 (i.e. the over prediction relative frequency is 0.32 compared to 0.20). This issue will be addressed by new science updates in the CMAQ system and evaluation results are expected to improve in upcoming CMAQ releases. WRVv3.7 and CMAQv5.1 under-predict cloudy conditions over land 28% and 27% of the time, respectively. Resolving this issue will require changes to the WRF cloud parameterization.

Table S1.

Categorical Metric	CMAQv5.02 Photolysis		CMAQv5.1 Photolysis		WRFv3.7	
	Land	Ocean	Land	Ocean	Land	Ocean

Agreement Index	0.68	0.61	0.66	0.59	0.66	0.67
Over-predict clouds	0.20	0.32	**0.06**	**0.32**	0.06	0.20
Under-predict clouds	0.12	0.07	**0.27**	0.09	0.28	0.13

Reference:
Biazar, A.P., 2014: Cloud Correction and its Impact on Air Quality Simulations, presented at the 94th AMS Annual Meeting, Atlanta, GA.

Note about access to GOES datasets:
GOES albedo data used in this study were downloaded from the following site: http://satdas.nsstc.nasa.gov/. Note that access to this GOES dataset will eventually be available from a new address: http://www.nsstc.uah.edu/satdas

S.2 CMAQv5.1 CB05e51 AE6 Species Definitions (available with the CMAQv5.1 release code)

! Updated AOCIJ, AOMIJ, and AORGAJ definition for CMAQv5.1 based on recommendations from Havala Pye (Dec 2014)

! The formulas used in this file implicitly assume that the model-ready emission files were prepared using a GSPRO from the 2002 emissions platform or later, in which POC emissions (hence, the CMAQ species APOCI and APOCJ) represent pure organic carbon without any scaling factor for OM:OC ratios.

! Output variables that begin with 'PM' represent those in which a size cut was applied. For example, PM25_NA is all sodium that falls below 2.5 um diameter. These 'PM' variables are used for comparisons at IMPROVE and STN sites.

! Output variables beginning with 'A' (aside from AIR_DENS) represent a combination of aerosol species in which no size cut was applied. For example, ASO4IJ is the sum of i-mode and j-mode sulfate. These 'A' variables are used for comparisons at CASTNet sites.

! Output variables beginning with 'PMC' is the coarse mode of total PM, i.e., sums all modes then subtracts the fine mode (PM2.5). These 'PMC' variables are used for comparisons at SEARCH sites.

```
/ File [1]: CMAQ conc/aconc file
/ File [2]: AEROVIS file
/ File [3]: METCRO3D file
/ File [4]: AERODIAM file
/ File [5]: METCRO2D file
/
/new species    ,units     ,expression

! Gases
ALD2            ,ppbV      ,1000.0*ALD2[1]
ALDX            ,ppbV      ,1000.0*ALDX[1]
BENZENE         ,ppbV      ,1000.0*BENZENE[1]
CO              ,ppbV      ,1000.0*CO[1]
ETH             ,ppbV      ,1000.0*ETH[1]
ETHA            ,ppbV      ,1000.0*ETHA[1]
FORM            ,ppbV      ,1000.0*FORM[1]
H2O2            ,ppbV      ,1000.0*H2O2[1]
HNO3            ,ppbV      ,1000.0*HNO3[1]
HNO3_UGM3       ,ug/m3     ,1000.0*(HNO3[1]*2.1756*DENS[3])
HONO            ,ppbV      ,1000.0*HONO[1]
CLNO2           ,ppbV      ,1000.0*CLNO2[1]
HOX             ,ppbV      ,1000.0*(OH[1]+HO2[1])
OH              ,ppbV      ,1000.0*(OH[1])
IOLE            ,ppbV      ,1000.0*IOLE[1]
ISOP            ,ppbV      ,1000.0*ISOP[1]
N2O5            ,ppbV      ,1000.0*N2O5[1]
NH3             ,ppbV      ,1000.0*NH3[1]
NH3_UGM3        ,ug/m3     ,1000.0*(NH3[1]*0.5880*DENS[3])
NHX             ,ug/m3     ,1000.0*(NH3[1]*0.5880*DENS[3])+ANH4I[1]+ANH4J[1]+ANH4K[1]
NO              ,ppbV      ,1000.0*NO[1]
NO2             ,ppbV      ,1000.0*NO2[1]
ANO3_PPB        ,ppbV      ,(ANO3I[1]+ANO3J[1]+ANO3K[1])/(DENS[3]*(62.0/28.97))
NTR             ,ppbV      ,1000.0*(NTROH[1]+NTRALK[1]+NTRCN[1]+NTRCNOH[1]+NTRM[1]+NTRI[1]+NTRPX[1])
PANS            ,ppbV      ,1000.0*(PAN[1]+PANX[1]+OPAN[1]+MAPAN[1])
NOY             ,ppbV
,1000.0*(NO[1]+NO2[1]+NO3[1]+2*N2O5[1]+HONO[1]+HNO3[1]+PNA[1]+CRON[1]+CRNO[1]+CRN2[1]+CRPX[1]+CL
NO2[1])+PANS[0]+NTR[0]+ANO3_PPB[0]
O3              ,ppbV      ,1000.0*O3[1]
OLE             ,ppbV      ,1000.0*OLE[1]
PAR             ,ppbV      ,1000.0*PAR[1]
PAN             ,ppbV      ,1000.0*PAN[1]
PANX            ,ppbV      ,1000.0*PANX[1]
```

SO2 ,ppbV ,1000.0*SO2[1]
SO2_UGM3 ,ug/m3 ,1000.0*(SO2[1]*2.2118*DENS[3])
SULF ,ppbV ,1000.0*SULF[1]
TERP ,ppbV ,1000.0*TERP[1]
TOL ,ppbV ,1000.0*TOL[1]
VOC ,ppbC
,1000.0*(PAR[1]+2.0*ETH[1]+2.0*ETOH[1]+2.0*OLE[1]+7.0*TOL[1]+8.0*XYLMN[1]+FORM[1]+2.0*ALD2[1]+5.0*ISOP[1]+2.0*ETHA[1]+4.0*IOLE[1]+2.0*ALDX[1]+10.0*TERP[1]+10.0*NAPH[1])
XYLMN ,ppbV ,1000.0*XYLMN[1]

! Particles
!! crustal elements
AFEJ ,ug/m3 ,AFEJ[1]
AALJ ,ug/m3 ,AALJ[1]
ASIJ ,ug/m3 ,ASIJ[1]
ATIJ ,ug/m3 ,ATIJ[1]
ACAJ ,ug/m3 ,ACAJ[1]
AMGJ ,ug/m3 ,AMGJ[1]
AKJ ,ug/m3 ,AKJ[1]
AMNJ ,ug/m3 ,AMNJ[1]
ASOILJ ,ug/m3 ,2.20*AALJ[1]+2.49*ASIJ[1]+1.63*ACAJ[1]+2.42*AFEJ[1]+1.94*ATIJ[1]
!! other PM species
AHPLUSIJ ,ug/m3 ,(AH3OPI[1]+AH3OPJ[1])*1.0/19.0
ANAK ,ug/m3 ,0.8373*ASEACAT[1]+0.0626*ASOIL[1]+0.0023*ACORS[1]
AMGK ,ug/m3 ,0.0997*ASEACAT[1] +0.0032*ACORS[1]
AKK ,ug/m3 ,0.0310*ASEACAT[1]+0.0242*ASOIL[1]+0.0176*ACORS[1]
ACAK ,ug/m3 ,0.0320*ASEACAT[1]+0.0838*ASOIL[1]+0.0562*ACORS[1]
ACLIJ ,ug/m3 ,ACLI[1]+ACLJ[1]
AECIJ ,ug/m3 ,AECI[1]+AECJ[1]
ANAIJ ,ug/m3 ,ANAJ[1]+ANAI[1]
ANO3IJ ,ug/m3 ,ANO3I[1]+ANO3J[1]
ANO3K ,ug/m3 ,ANO3K[1]
TNO3 ,ug/m3 ,2175.6*(HNO3[1]*DENS[3])+ANO3I[1]+ANO3J[1]+ANO3K[1]
ANH4IJ ,ug/m3 ,ANH4I[1]+ANH4J[1]
ANH4K ,ug/m3 ,ANH4K[1]
AOCIJ ,ugC/m3
,(AXYL1J[1]+AXYL2J[1]+AXYL3J[1])/2.0+(ATOL1J[1]+ATOL2J[1]+ATOL3J[1])/2.0+(ABNZ1J[1]+ABNZ2J[1]+ABNZ3J[1])/2.0
+(AISO1J[1]+AISO2J[1])/1.6+AISO3J[1]/2.7+(ATRP1J[1]+ATRP2J[1])/1.4+ASQTJ[1]/2.1+AALK1J[1]/1.17+AALK2J[1]/1.17+AORGCJ[1]/2.0+(AOLGBJ[1]+AOLGAJ[1])/2.1+APOCI[1]+APOCJ[1]+(APAH1J[1]+APAH2J[1]+APAH3J[1])/2.03
AOMIJ ,ug/m3
,AXYL1J[1]+AXYL2J[1]+AXYL3J[1]+ATOL1J[1]+ATOL2J[1]+ATOL3J[1]+ABNZ1J[1]+ABNZ2J[1]+ABNZ3J[1]+AISO1J[1]+AISO2J[1]+AISO3J[1]+ATRP1J[1]+ATRP2J[1]+ASQTJ[1]+AALK1J[1]+AALK2J[1]+AORGCJ[1]+AOLGBJ[1]+AOLGAJ[1]+APOCI[1]+APOCJ[1]+APNCOMI[1]+APNCOMJ[1]+APAH1J[1]+APAH2J[1]+APAH3J[1]
AORGAJ ,ug/m3
,AXYL1J[1]+AXYL2J[1]+AXYL3J[1]+ATOL1J[1]+ATOL2J[1]+ATOL3J[1]+ABNZ1J[1]+ABNZ2J[1]+ABNZ3J[1]+AALK1J[1]+AALK2J[1]+AOLGAJ[1]+APAH1J[1]+APAH2J[1]+APAH3J[1]
AORGBJ ,ug/m3 ,AISO1J[1]+AISO2J[1]+AISO3J[1]+ATRP1J[1]+ATRP2J[1]+ASQTJ[1]+AOLGBJ[1]
AORGCJ ,ug/m3 ,AORGCJ[1]
APOCIJ ,ugC/m3 ,APOCI[1]+APOCJ[1]
APOAIJ ,ug/m3 ,APOCIJ[0]+APNCOMI[1]+APNCOMJ[1]
ASO4IJ ,ug/m3 ,ASO4I[1]+ASO4J[1]
ASO4K ,ug/m3 ,ASO4K[1]
ATOTI ,ug/m3
,ASO4I[1]+ANO3I[1]+ANH4I[1]+ANAI[1]+ACLI[1]+AECI[1]+APOCI[1]+APNCOMI[1]+AOTHRI[1]
ATOTJ ,ug/m3 ,ASO4J[1]+ANO3J[1]+ANH4J[1]+ANAJ[1]+ACLJ[1]+AECJ[1]+AOMIJ[0]-(APOCI[1]+APNCOMI[1])+AOTHRJ[1]+AFEJ[1]+ASIJ[1]+ATIJ[1]+ACAJ[1]+AMGJ[1]+AMNJ[1]+AALJ[1]+AKJ[1]
ATOTK ,ug/m3 ,ASOIL[1]+ACORS[1]+ASEACAT[1]+ACLK[1]+ASO4K[1]+ANO3K[1]+ANH4K[1]
PMIJ ,ug/m3 ,ATOTI[0]+ATOTJ[0]
PM10 ,ug/m3 ,PMIJ[0]+ATOTK[0]

```
AUNSPEC1IJ      ,ug/m3     ,PMIJ[0] - (ASOILJ[0] + ANO3IJ[0] + ASO4IJ[0] + ANH4IJ[0] + AOCIJ[0] + AECIJ[0] +
ANAIJ[0] + ACLIJ[0])
ANCOMIJ         ,ug/m3     ,AOMIJ[0]-AOCIJ[0]
AUNSPEC2IJ      ,ug/m3     ,AUNSPEC1IJ[0] - ANCOMIJ[0]
!! OM/OC ratios
AOMOCRAT_PRI     ,none      ,APOAIJ[0]/APOCIJ[0]
AOMOCRAT_TOT     ,none      ,AOMIJ[0]/AOCIJ[0]

!! PM2.5 sharp cutoff species
PM25_HP         ,ug/m3     ,(AH3OPI[1]*PM25AT[4]+AH3OPJ[1]*PM25AC[4]+AH3OPK[1]*PM25CO[4])*1.0/19.0
PM25_CL         ,ug/m3     ,ACLI[1]*PM25AT[4]+ACLJ[1]*PM25AC[4]+ACLK[1]*PM25CO[4]
PM25_EC         ,ug/m3     ,AECI[1]*PM25AT[4]+AECJ[1]*PM25AC[4]
PM25_NA         ,ug/m3     ,ANAI[1]*PM25AT[4]+ANAJ[1]*PM25AC[4]+ANAK[0]*PM25CO[4]
PM25_MG         ,ug/m3     ,                  AMGJ[1]*PM25AC[4]+AMGK[0]*PM25CO[4]
PM25_K          ,ug/m3     ,                  AKJ[1] *PM25AC[4]+AKK[0] *PM25CO[4]
PM25_CA         ,ug/m3     ,                  ACAJ[1]*PM25AC[4]+ACAK[0]*PM25CO[4]
PM25_NH4        ,ug/m3     ,ANH4I[1]*PM25AT[4]+ANH4J[1]*PM25AC[4]+ANH4K[1]*PM25CO[4]
PM25_NO3        ,ug/m3     ,ANO3I[1]*PM25AT[4]+ANO3J[1]*PM25AC[4]+ANO3K[1]*PM25CO[4]
PM25_OC         ,ugC/m3    ,APOCI[1]*PM25AT[4]+(AOCIJ[0]-APOCI[1])*PM25AC[4]
PM25_SOIL       ,ug/m3     ,ASOILJ[0]*PM25AC[4]+(ASOIL[1]+ACORS[1])*PM25CO[4]
PM25_SO4        ,ug/m3     ,ASO4I[1]*PM25AT[4]+ASO4J[1]*PM25AC[4]+ASO4K[1]*PM25CO[4]
PM25_TOT        ,ug/m3     ,ATOTI[0]*PM25AT[4]+ATOTJ[0]*PM25AC[4]+ATOTK[0]*PM25CO[4]
PM25_UNSPEC1    ,ug/m3     ,PM25_TOT[0]-
(PM25_CL[0]+PM25_EC[0]+PM25_NA[0]+PM25_NH4[0]+PM25_NO3[0]+PM25_OC[0]+PM25_SOIL[0]+PM25_SO4[0])
PMC_CL          ,ug/m3     ,ACLI[1]+ACLJ[1]+ACLK[1]-PM25_CL[0]
PMC_NA          ,ug/m3     ,ANAIJ[0]+ANAK[0]*0.78-PM25_NA[0]
PMC_NH4         ,ug/m3     ,ANH4I[1]+ANH4J[1]+ANH4K[1]-PM25_NH4[0]
PMC_NO3         ,ug/m3     ,ANO3I[1]+ANO3J[1]+ANO3K[1]-PM25_NO3[0]
PMC_SO4         ,ug/m3     ,ASO4I[1]+ASO4J[1]+ASO4K[1]-PM25_SO4[0]
PMC_TOT         ,ug/m3     ,PM10[0]-PM25_TOT[0]


!Meteorology
DCV_Recon       ,deciview  ,DCV_Recon[2]
AIR_DENS        ,kg/m3     ,DENS[3]
RH              ,%         ,100.00*RH[4]
SFC_TMP         ,C         ,(TEMP2[5]-273.15)
PBLH            ,m         ,PBL[5]
SOL_RAD         ,WATTS/m2  ,RGRND[5]
precip          ,cm        ,RN[5]+RC[5]
WSPD10          ,m/s       ,WSPD10[5]
WDIR10          ,deg       ,WDIR10[5]

!FRM PM Equivalent Calculation
K               ,ppb^2   ,exp(118.87-24084/TEMP2[5]-6.025*log(TEMP2[5]))
P1              ,         ,exp(8763/TEMP2[5]+19.12*log(TEMP2[5])-135.94)
P2              ,         ,exp(9969/TEMP2[5]+16.22*log(TEMP2[5])-122.65)
P3              ,         ,exp(13875/TEMP2[5]+24.46*log(TEMP2[5])-182.61)
a               ,         ,1-RH[0]/100
K_prime         ,ppb^2     ,(P1[0]-P2[0]*a[0]+(P3[0]*a[0]*a[0]))*(a[0]^1.75)*K[0]
sqrt_Ki         ,ppb       ,sqrt(RH[0]<=61 ? K[0] : K_prime[0])
max_NO3_loss    ,ug/m3     ,745.7/TEMP2[5]*sqrt_Ki[0]
PM25_NO3_loss   ,ug/m3     ,max_NO3_loss[0]<=PM25_NO3[0] ? max_NO3_loss[0] : PM25_NO3[0]
ANO3IJ_loss     ,ug/m3     ,max_NO3_loss[0]<=ANO3IJ[0] ? max_NO3_loss[0] : ANO3IJ[0]
PM25_NH4_loss   ,ug/m3     ,PM25_NO3_loss[0]*(18/62)
ANH4IJ_loss     ,ug/m3     ,ANO3IJ_loss[0]*(18/62)
PMIJ_FRM        ,ug/m3     ,PMIJ[0]-(ANO3IJ_loss[0]+ANH4IJ_loss[0])+0.24*(ASO4IJ[0]+ANH4IJ[0]-ANH4IJ_loss[0])+0.5
PM25_FRM        ,ug/m3     ,PM25_TOT[0]-(PM25_NO3_loss[0]+PM25_NH4_loss[0])+0.24*(PM25_SO4[0]+PM25_NH4[0]-
PM25_NH4_loss[0])+0.5
```

S.3 CMAQv5.1 CB05e51 AE6 Wet/Dry Deposition Species Definitions (available with the CMAQv5.1 release code)

```
/ File [1]: DRYDEP
/ File [2]: WETDEP
/ File [3]: METCRO2D
/
/new species       ,units     ,expression

ANAK_D            ,kg/ha ,0.8373*ASEACAT[1]+0.0626*ASOIL[1]+0.0023*ACORS[1]
ANAK_W            ,kg/ha ,0.8373*ASEACAT[2]+0.0626*ASOIL[2]+0.0023*ACORS[2]
AMGK_D            ,kg/ha ,0.0997*ASEACAT[1]                +0.0032*ACORS[1]
AMGK_W            ,kg/ha ,0.0997*ASEACAT[2]                +0.0032*ACORS[2]
AKK_D             ,kg/ha ,0.0310*ASEACAT[1]+0.0242*ASOIL[1]+0.0176*ACORS[1]
AKK_W             ,kg/ha ,0.0310*ASEACAT[2]+0.0242*ASOIL[2]+0.0176*ACORS[2]
ACAK_D            ,kg/ha ,0.0320*ASEACAT[1]+0.0838*ASOIL[1]+0.0562*ACORS[1]
ACAK_W            ,kg/ha ,0.0320*ASEACAT[2]+0.0838*ASOIL[2]+0.0562*ACORS[2]

DDEP_NO2          ,kg/ha     ,NO2[1]
WDEP_NO2          ,kg/ha     ,NO2[2]
DDEP_NO           ,kg/ha     ,NO[1]
WDEP_NO           ,kg/ha     ,NO[2]
DDEP_FORM         ,kg/ha     ,FORM[1]
WDEP_FORM         ,kg/ha     ,FORM[2]
DDEP_H2O2         ,kg/ha     ,H2O2[1]
WDEP_H2O2         ,kg/ha     ,H2O2[2]
DDEP_N2O5         ,kg/ha     ,N2O5[1]
WDEP_N2O5         ,kg/ha     ,N2O5[2]
DDEP_HONO         ,kg/ha     ,HONO[1]
WDEP_HONO         ,kg/ha     ,HONO[2]
DDEP_HNO3         ,kg/ha     ,HNO3[1]
WDEP_HNO3         ,kg/ha     ,HNO3[2]
DDEP_ANO3IJ       ,kg/ha     ,ANO3I[1] + ANO3J[1]
DDEP_ANO3K        ,kg/ha     ,ANO3K[1]
WDEP_ANO3IJK      ,kg/ha     ,ANO3I[2] + ANO3J[2] + ANO3K[2]
DDEP_TNO3         ,kg/ha     ,ANO3I[1] + ANO3J[1] + ANO3K[1] + 0.984*HNO3[1]
WDEP_TNO3         ,kg/ha     ,ANO3I[2] + ANO3J[2] + ANO3K[2] + 0.984*HNO3[2]
DDEP_NTR          ,kg/ha     ,NTROH[1]+NTRALK[1]+NTRCN[1]+NTRPX[1]+NTRCNOH[1] + NTRM[1]+NTRI[1]
WDEP_NTR          ,kg/ha     ,NTROH[2]+NTRALK[2]+NTRCN[2]+NTRPX[2]+NTRCNOH[2] + NTRM[2]+NTRI[2]
DDEP_PANT         ,kg/ha     ,PAN[1] + PANX[1] + OPAN[1] + MAPAN[1]
WDEP_PANT         ,kg/ha     ,PAN[2] + PANX[2] + OPAN[2] + MAPAN[2]
DDEP_NH3          ,kg/ha     ,NH3[1]
WDEP_NH3          ,kg/ha     ,NH3[2]
DDEP_ANH4IJ       ,kg/ha     ,ANH4I[1] + ANH4J[1]
DDEP_ANH4K        ,kg/ha     ,ANH4K[1]
WDEP_ANH4IJK      ,kg/ha     ,ANH4I[2] + ANH4J[2] + ANH4K[2]
DDEP_NHX          ,kg/ha     ,ANH4I[1] + ANH4J[1] + ANH4K[1] + 1.059*NH3[1]
WDEP_NHX          ,kg/ha     ,ANH4I[2] + ANH4J[2] + ANH4K[2] + 1.059*NH3[2]
DDEP_SO2          ,kg/ha     ,SO2[1]
DDEP_ASO4IJ       ,kg/ha     ,ASO4I[1] + ASO4J[1]
DDEP_ASO4K        ,kg/ha     ,ASO4K[1]
WDEP_ASO4IJK      ,kg/ha     ,ASO4I[2] + ASO4J[2] + ASO4K[2]
WDEP_TSO4         ,kg/ha     ,ASO4I[2] + ASO4J[2] + ASO4K[2] + 1.5*SO2[2]
DDEP_AECIJ        ,kg/ha     ,AECI[1] +AECJ[1]
DDEP_AOCIJ        ,kg/ha
,(AXYL1J[1]+AXYL2J[1]+AXYL3J[1])/2.0+(ATOL1J[1]+ATOL2J[1]+ATOL3J[1])/2.0+(ABNZ1J[1]+ABNZ2J[1]+ABNZ3J[
1])/2.0+(AISO1J[1]+AISO2J[1])/1.6+AISO3J[1]/2.7+(ATRP1J[1]+ATRP2J[1])/1.4+ASQTJ[1]/2.1+0.64*(AALK1J[1]+AALK
2J[1])+(APAH1J[1]+APAH2J[1]+APAH3J[1])/2.03+AORGCJ[1]/2.0 +(AOLGBJ[1]+AOLGAJ[1])/2.1+APOCI[1]+APOCJ[1]
DDEP_SSSO4J       ,kg/ha     ,0.19579876*ANAJ[1]
```

DDEP_SSSO4K ,kg/ha ,0.19579876*ANAK_D[0]
WDEP_SSSO4JK ,kg/ha ,0.19579876*ANAJ[2] + 0.19579876*ANAK_W[0]
DDEP_ANAJ ,kg/ha ,ANAJ[1]
DDEP_ANAK ,kg/ha ,ANAK_D[0]
DDEP_ANAJK ,kg/ha ,ANAJ[1] + ANAK_D[0]
WDEP_ANAJK ,kg/ha ,ANAJ[2] + ANAK_W[0]
TDEP_ANAJK ,kg/ha ,DDEP_ANAJK[0] + WDEP_ANAJK[0]
DDEP_ACLJ ,kg/ha ,ACLJ[1]
DDEP_ACLK ,kg/ha ,ACLK[1]
DDEP_ACLJK ,kg/ha ,ACLJ[1] + ACLK[1]
WDEP_TCL ,kg/ha ,0.972*HCL[2]+0.435*CLNO2[2] + ACLJ[2] + ACLK[2]
TDEP_CL ,kg/ha ,DDEP_ACLJK[0] + WDEP_TCL[0]
DDEP_CAJ ,kg/ha ,ACAJ[1]
WDEP_CAJ ,kg/ha ,ACAJ[2]
DDEP_CAJK ,kg/ha ,ACAJ[1]+ACAK_D[0]
WDEP_CAJK ,kg/ha ,ACAJ[2]+ACAK_W[0]
DDEP_FEJ ,kg/ha ,AFEJ[1]
WDEP_FEJ ,kg/ha ,AFEJ[2]
DDEP_ALJ ,kg/ha ,AALJ[1]
WDEP_ALJ ,kg/ha ,AALJ[2]
DDEP_SIJ ,kg/ha ,ASIJ[1]
WDEP_SIJ ,kg/ha ,ASIJ[2]
DDEP_TIJ ,kg/ha ,ATIJ[1]
WDEP_TIJ ,kg/ha ,ATIJ[2]
DDEP_MGJ ,kg/ha ,AMGJ[1]
WDEP_MGJ ,kg/ha ,AMGJ[2]
DDEP_MGJK ,kg/ha ,AMGJ[1]+AMGK_D[0]
WDEP_MGJK ,kg/ha ,AMGJ[2]+AMGK_W[0]
DDEP_KJ ,kg/ha ,AKJ[1]
WDEP_KJ ,kg/ha ,AKJ[2]
DDEP_KJK ,kg/ha ,AKJ[1]+AKK_D[0]
WDEP_KJK ,kg/ha ,AKJ[2]+AKK_W[0]
DDEP_MNJ ,kg/ha ,AMNJ[1]
WDEP_MNJ ,kg/ha ,AMNJ[2]
DDEP_O3 ,kg/ha ,O3[1]
WDEP_O3 ,kg/ha ,O3[2]
WDEP_PNA ,kg/ha ,PNA[2]
RT ,cm ,RN[3] + RC[3]
DD_OXN_NOX ,kg/ha ,0.30435*NO2[1] + 0.46667*NO[1]
WD_OXN_NOX ,kg/ha ,0.30435*NO2[2] + 0.46667*NO[2]
DD_OXN_TNO3 ,kg/ha ,0.22222*HNO3[1] + 0.22581*ANO3I[1] + 0.22581*ANO3J[1] + 0.22581*ANO3K[1]
WD_OXN_TNO3 ,kg/ha ,0.22581*WDEP_TNO3[0]
DD_OXN_PANT ,kg/ha ,0.11570*PAN[1] + 0.11570*PANX[1] + 0.11570*OPAN[1] + 0.11570*MAPAN[1]
WD_OXN_PANT ,kg/ha ,0.11570*PAN[2] + 0.11570*PANX[2] + 0.11570*OPAN[2] + 0.11570*MAPAN[2]
DD_OXN_ORGN ,kg/ha ,0.10770*(NTROH[1]+NTRALK[1]+NTRCN[1]+NTRPX[1]+NTRCNOH[1] + NTRM[1]+NTRI[1])
WD_OXN_ORGN ,kg/ha ,0.10770*(NTROH[2]+NTRALK[2]+NTRCN[2]+NTRPX[2]+NTRCNOH[2] + NTRM[2]+NTRI[2])
/DD_OXN_OTHR ,kg/ha ,0.25926*N2O5[1] + 0.29787*HONO[1]+0.1717*CLNO2[1]
WD_OXN_OTHR ,kg/ha ,0.25926*N2O5[2] + 0.29787*HONO[2]+0.177720*PNA[2]+0.1717*CLNO2[2]
/DD_OXN_TOT ,kg/ha ,DD_OXN_NOX[0] + DD_OXN_TNO3[0] + DD_OXN_PANT[0] + DD_OXN_ORGN[0] + DD_OXN_OTHR[0]
WD_OXN_TOT ,kg/ha ,WD_OXN_NOX[0] + WD_OXN_TNO3[0] + WD_OXN_PANT[0] + WD_OXN_ORGN[0] + WD_OXN_OTHR[0]
/TD_OXN_TOT ,kg/ha ,DD_OXN_TOT[0] + WD_OXN_TOT[0]
/DD_OXN_TOTMEQ ,meq/m2 ,7.14*DD_OXN_TOT[0]
WD_OXN_TOTMEQ ,meq/m2 ,7.14*WD_OXN_TOT[0]
/TD_OXN_TOTMEQ ,meq/m2 ,DD_OXN_TOTMEQ[0] + WD_OXN_TOTMEQ[0]
DD_REDN_TOT ,kg/ha ,0.7777*DDEP_NHX[0]
WD_REDN_TOT ,kg/ha ,0.7777*WDEP_NHX[0]

```
TD_REDN_TOT        ,kg/ha     ,DD_REDN_TOT[0] + WD_REDN_TOT[0]
DD_REDN_TOTMEQ     ,meq/m2    ,7.14*DD_REDN_TOT[0]
WD_REDN_TOTMEQ     ,meq/m2    ,7.14*WD_REDN_TOT[0]
TD_REDN_TOTMEQ     ,meq/m2    ,DD_REDN_TOTMEQ[0] + WD_REDN_TOTMEQ[0]
DD_S_TOT           ,kg/ha     ,0.5*SO2[1] + 0.33333*ASO4I[1] + 0.33333*ASO4J[1] + 0.33333*ASO4K[1]
WD_S_TOT           ,kg/ha     ,0.33333*WDEP_TSO4[0]
TD_S_TOT           ,kg/ha     ,DD_S_TOT[0] + WD_S_TOT[0]
DD_S_TOTMEQ        ,meq/m2    ,6.24*DD_S_TOT[0]
WD_S_TOTMEQ        ,meq/m2    ,6.24*WD_S_TOT[0]
TD_S_TOTMEQ        ,meq/m2    ,DD_S_TOTMEQ[0] + WD_S_TOTMEQ[0]
DD_S_SeaS          ,kg/ha     ,0.33333*DDEP_SSSO4J[0] + 0.33333*DDEP_SSSO4K[0]
WD_S_SeaS          ,kg/ha     ,0.33333*WDEP_SSSO4JK[0]
TD_S_SeaS          ,kg/ha     ,DD_S_SeaS[0] + WD_S_SeaS[0]
DD_S_SeaSMEQ       ,meq/m2    ,6.24*DD_S_SeaS[0]
WD_S_SeaSMEQ       ,meq/m2    ,6.24*WD_S_SeaS[0]
TD_S_SeaSMEQ       ,meq/m2    ,DD_S_SeaSMEQ[0] + WD_S_SeaSMEQ[0]
```

S.4 – WRFv3.4 Namelist

```
&time_control
 start_year                          = 2011,
 start_month                         = 12,
 start_day                           = 27,
 start_hour                          = 00,
 start_minute                        = 00,
 start_second                        = 00,
 end_year                            = 2012,
 end_month                           = 01,
 end_day                             = 01,
 end_hour                            = 00,
 end_minute                          = 00,
 end_second                          = 00,
 interval_seconds                    = 10800,
 input_from_file                     = .true.,
 history_interval                    = 60,
 frames_per_outfile                  = 24,
 restart                             = .TRUE.,
 restart_interval                    = 7200,
 io_form_history                     = 2
 io_form_restart                     = 2
 io_form_input                       = 2
 io_form_boundary                    = 2
 debug_level                         = 0
 io_form_auxinput2                   = 2
 io_form_auxinput4                   = 2
 auxinput1_inname                    = "metoa_em.d01.<date>"
 auxinput4_inname                    = "wrflowinp_d01"
 auxinput4_interval                  = 180
 auxinput4_end_h                     = 9001
 write_hist_at_0h_rst                = .true.,
 /

 &domains
 time_step                           = 60,
 time_step_fract_num                 = 0,
 time_step_fract_den                 = 1,
 use_adaptive_time_step              = .false.
 max_dom                             = 1,
 s_we                                = 1,
 e_we                                = 472,
 s_sn                                = 1,
 e_sn                                = 312,
 s_vert                              = 1,
 e_vert                              = 36,
 p_top_requested                     = 5000,
 eta_levels                          = 1.000, 0.9975, 0.995, 0.990, 0.985,
                                   0.980, 0.970, 0.960, 0.950,
                                   0.940, 0.930, 0.920, 0.910,
                                   0.900, 0.880, 0.860, 0.840,
                                   0.820, 0.800, 0.770, 0.740,
                                   0.700, 0.650, 0.600, 0.550,
                                   0.500, 0.450, 0.400, 0.350,
                                   0.300, 0.250, 0.200, 0.150,
                                   0.100, 0.050, 0.000
 dx                                = 12000,
 dy                                = 12000,
 grid_id                             = 1,
```

```
parent_id                          = 0,
i_parent_start                     = 0,
j_parent_start                     = 0,
parent_grid_ratio                  = 1,
parent_time_step_ratio             = 1,
feedback                           = 1,
smooth_option                      = 0,
/

&physics
mp_physics                         = 10,
ra_lw_physics                      = 4,
ra_sw_physics                      = 4,
radt                               = 20,
sf_sfclay_physics                  = 7,
sf_surface_physics                 = 7,
bl_pbl_physics                     = 7,
bldt                               = 0,
cu_physics                         = 1,
kfeta_trigger                      = 2,
cudt                               = 0,
isfflx                             = 1,
ifsnow                             = 1,
icloud                             = 1,
surface_input_source               = 1,
num_soil_layers                    = 2,
sst_update                         = 1,
pxlsm_smois_init                   = 0,
slope_rad                          = 1,
topo_shading                       = 1,
shadlen                            = 25000.,
num_land_cat                       = 40,
prec_acc_dt                        = 60,
mp_zero_out                        = 2,
fractional_seaice                  = 1,
seaice_threshold                   = 0.0,
/

&fdda
grid_fdda                          = 1,
grid_sfdda                         = 1,
pxlsm_soil_nudge                   = 1,
sgfdda_inname                      = "wrfsfdda_d01",
sgfdda_end_h                       = 9001,
sgfdda_interval_m                  = 180,
sgfdda_interval                    = 10800,
gfdda_inname                       = "wrffdda_d<domain>",
gfdda_end_h                        = 9001,
gfdda_interval_m                   = 180,
fgdt                               = 0,
if_no_pbl_nudging_uv               = 1,
if_no_pbl_nudging_t                = 1,
if_no_pbl_nudging_q                = 1,
if_zfac_uv                         = 0,
 k_zfac_uv                         = 13,
if_zfac_t                          = 0,
 k_zfac_t                          = 13,
if_zfac_q                          = 0,
 k_zfac_q                          = 13,
guv                                = 0.0001,
```

```
gt                              = 0.0001,
gq                              = 0.00001,
guv_sfc                         = 0.0000,
gt_sfc                          = 0.0000,
gq_sfc                          = 0.0000,
if_ramping                      = 0,
dtramp_min                      = 60.0,
io_form_gfdda                   = 2,
rinblw                          = 250.0
/

&dynamics
w_damping                       = 1,
diff_opt                        = 1,
km_opt                          = 4,
diff_6th_opt                    = 2,
diff_6th_factor                 = 0.12,
damp_opt                        = 3,
base_temp                       = 290.
zdamp                           = 5000.,
dampcoef                        = 0.05,
khdif                           = 0,
kvdif                           = 0,
non_hydrostatic                 = .true.,
moist_adv_opt                   = 2,
tke_adv_opt                     = 2,
scalar_adv_opt                  = 2,
/

&dfi_control
dfi_opt                         = 0
dfi_nfilter                     = 7
dfi_write_filtered_input        = .true.
dfi_write_dfi_history           = .false.
dfi_cutoff_seconds              = 60
dfi_time_dim                    = 1000
dfi_bckstop_year                = 2006
dfi_bckstop_month               = 08
dfi_bckstop_day                 = 04
dfi_bckstop_hour                = 12
dfi_bckstop_minute              = 00
dfi_bckstop_second              = 00
dfi_fwdstop_year                = 2006
dfi_fwdstop_month               = 08
dfi_fwdstop_day                 = 04
dfi_fwdstop_hour                = 13
dfi_fwdstop_minute              = 00
dfi_fwdstop_second              = 00
/

&bdy_control
spec_bdy_width                  = 5,
spec_zone                       = 1,
relax_zone                      = 4,
specified                       = .true.,
nested                          = .false.,
/

&grib2
/
```

```
&namelist_quilt
nio_tasks_per_group = 0,
nio_groups = 1,
/
```

S.5 – WRFv3.7 Namelist

```
&time_control
 start_year                          = 2011,
 start_month                         = 12,
 start_day                           = 27,
 start_hour                          = 00,
 start_minute                        = 00,
 start_second                        = 00,
 end_year                            = 2012,
 end_month                           = 01,
 end_day                             = 01,
 end_hour                            = 00,
 end_minute                          = 00,
 end_second                          = 00,
 interval_seconds                    = 10800,
 input_from_file                     = .true.,
 history_interval                    = 60,
 frames_per_outfile                  = 24,
 restart                             = .TRUE.,
 restart_interval                    = 7200,
 io_form_history                     = 2
 io_form_restart                     = 2
 io_form_input                       = 2
 io_form_boundary                    = 2
 debug_level                         = 0
 io_form_auxinput2                   = 2
 io_form_auxinput4                   = 2
 auxinput1_inname                    = "metoa_em.d01.<date>"
 auxinput4_inname                    = "wrflowinp_d01"
 auxinput4_interval                  = 180
 auxinput4_end_h                     = 9001
 write_hist_at_0h_rst                = .true.,
 /

 &domains
 time_step                           = 60,
 time_step_fract_num                 = 0,
 time_step_fract_den                 = 1,
 use_adaptive_time_step              = .false.
 max_dom                             = 1,
 s_we                                = 1,
 e_we                                = 472,
 s_sn                                = 1,
 e_sn                                = 312,
 s_vert                              = 1,
 e_vert                              = 36,
 p_top_requested                     = 5000,
 eta_levels                          = 1.000, 0.9975, 0.995, 0.990, 0.985,
                                  0.980, 0.970, 0.960, 0.950,
                                  0.940, 0.930, 0.920, 0.910,
                                  0.900, 0.880, 0.860, 0.840,
                                  0.820, 0.800, 0.770, 0.740,
                                  0.700, 0.650, 0.600, 0.550,
                                  0.500, 0.450, 0.400, 0.350,
                                  0.300, 0.250, 0.200, 0.150,
                                  0.100, 0.050, 0.000
 dx                               = 12000,
 dy                               = 12000,
 grid_id                             = 1,
```

```
parent_id                          = 0,
i_parent_start                     = 0,
j_parent_start                     = 0,
parent_grid_ratio                  = 1,
parent_time_step_ratio             = 1,
feedback                           = 1,
smooth_option                      = 0,
/

&physics
mp_physics                         = 10,
ra_lw_physics                      = 4,
ra_sw_physics                      = 4,
radt                               = 20,
sf_sfclay_physics                  = 7,
sf_surface_physics                 = 7,
bl_pbl_physics                     = 7,
bldt                               = 0,
cu_physics                         = 1,
kfeta_trigger                      = 2,
cudt                               = 0,
isfflx                             = 1,
ifsnow                             = 1,
icloud                             = 1,
cu_rad_feedback                    = .true.,
surface_input_source               = 1,
num_soil_layers                    = 2,
sst_update                         = 1,
pxlsm_smois_init                   = 0,
slope_rad                          = 1,
topo_shading                       = 1,
shadlen                            = 25000.,
num_land_cat                       = 40,
prec_acc_dt                        = 60,
mp_zero_out                        = 2,
fractional_seaice                  = 1,
seaice_threshold                   = 0.0,
/

&fdda
grid_fdda                          = 1,
grid_sfdda                         = 1,
pxlsm_soil_nudge                   = 1,
sgfdda_inname                      = "wrfsfdda_d01",
sgfdda_end_h                       = 9001,
sgfdda_interval_m                  = 180,
sgfdda_interval                    = 10800,
gfdda_inname                       = "wrffdda_d<domain>",
gfdda_end_h                        = 9001,
gfdda_interval_m                   = 180,
fgdt                               = 0,
if_no_pbl_nudging_uv               = 1,
if_no_pbl_nudging_t                = 1,
if_no_pbl_nudging_q                = 1,
if_zfac_uv                         = 0,
 k_zfac_uv                         = 13,
if_zfac_t                          = 0,
 k_zfac_t                          = 13,
if_zfac_q                          = 0,
 k_zfac_q                          = 13,
```

```
guv                              = 0.0001,
gt                               = 0.0001,
gq                               = 0.00001,
guv_sfc                          = 0.0000,
gt_sfc                           = 0.0000,
gq_sfc                           = 0.0000,
if_ramping                       = 0,
dtramp_min                       = 60.0,
io_form_gfdda                    = 2,
rinblw                           = 250.0
/

&dynamics
w_damping                        = 1,
diff_opt                         = 1,
km_opt                           = 4,
diff_6th_opt                     = 2,
diff_6th_factor                  = 0.12,
damp_opt                         = 3,
base_temp                        = 290.
zdamp                            = 5000.,
dampcoef                         = 0.05,
khdif                            = 0,
kvdif                            = 0,
non_hydrostatic                  = .true.,
moist_adv_opt                    = 2,
tke_adv_opt                      = 2,
scalar_adv_opt                   = 2,
/

&dfi_control
dfi_opt                          = 0
dfi_nfilter                      = 7
dfi_write_filtered_input         = .true.
dfi_write_dfi_history            = .false.
dfi_cutoff_seconds               = 60
dfi_time_dim                     = 1000
dfi_bckstop_year                 = 2006
dfi_bckstop_month                = 08
dfi_bckstop_day                  = 04
dfi_bckstop_hour                 = 12
dfi_bckstop_minute               = 00
dfi_bckstop_second               = 00
dfi_fwdstop_year                 = 2006
dfi_fwdstop_month                = 08
dfi_fwdstop_day                  = 04
dfi_fwdstop_hour                 = 13
dfi_fwdstop_minute               = 00
dfi_fwdstop_second               = 00
/

&bdy_control
spec_bdy_width                   = 5,
spec_zone                        = 1,
relax_zone                       = 4,
specified                        = .true.,
nested                           = .false.,
/

&grib2
```

```
/

&namelist_quilt
nio_tasks_per_group = 0,
nio_groups = 1,
/
```

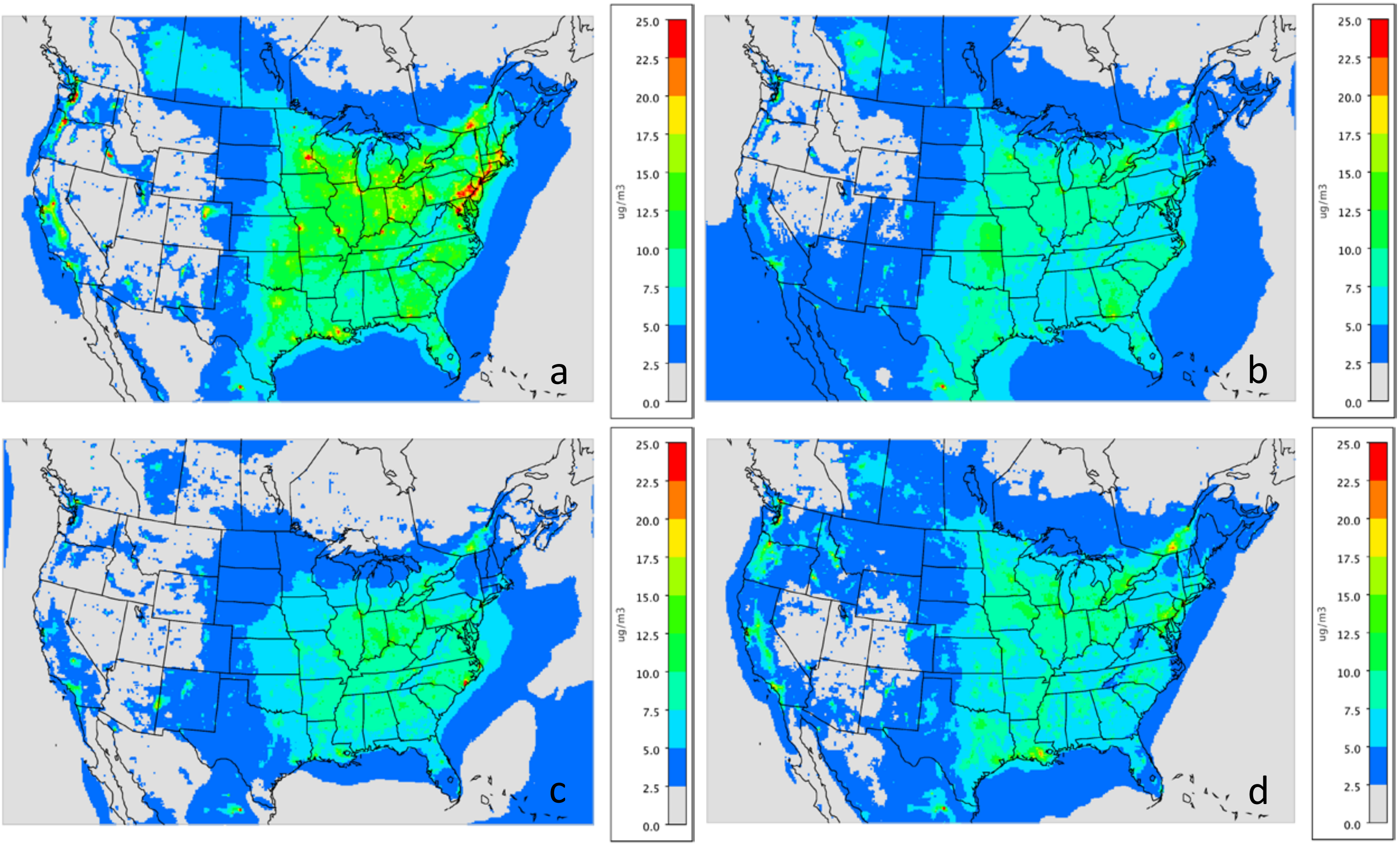

Figure S1: CMAQv5.0.2 simulation seasonal average $PM_{2.5}$ concentrations (μgm^{-3}) for a) winter b) spring c) summer and d) fall.

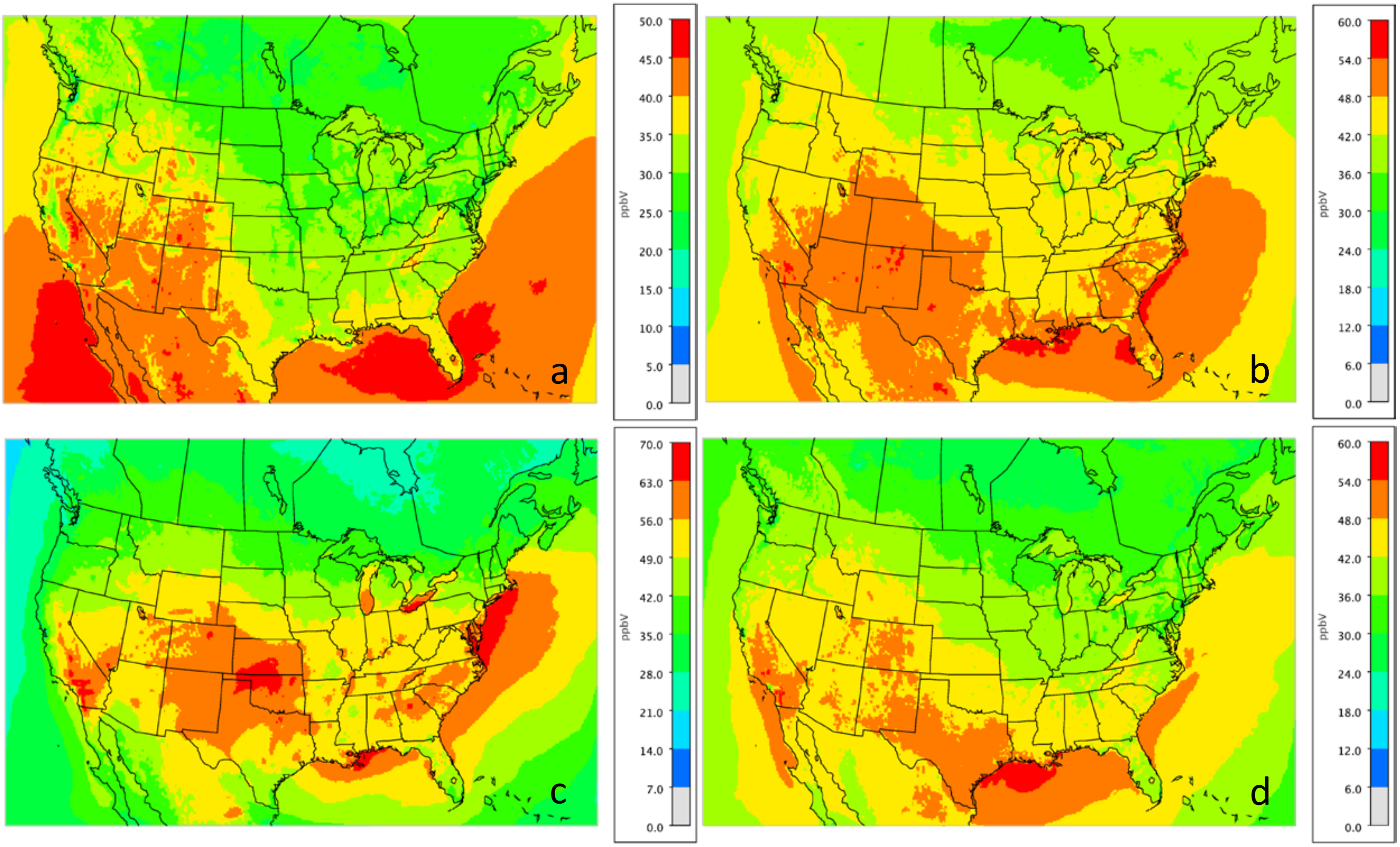

Figure S2: CMAQv5.0.2 simulation seasonal average MDA8 O_3 mixing ratio (ppbv) for a) winter b) spring c) summer and d) fall. Note that the scales for each plot can vary.

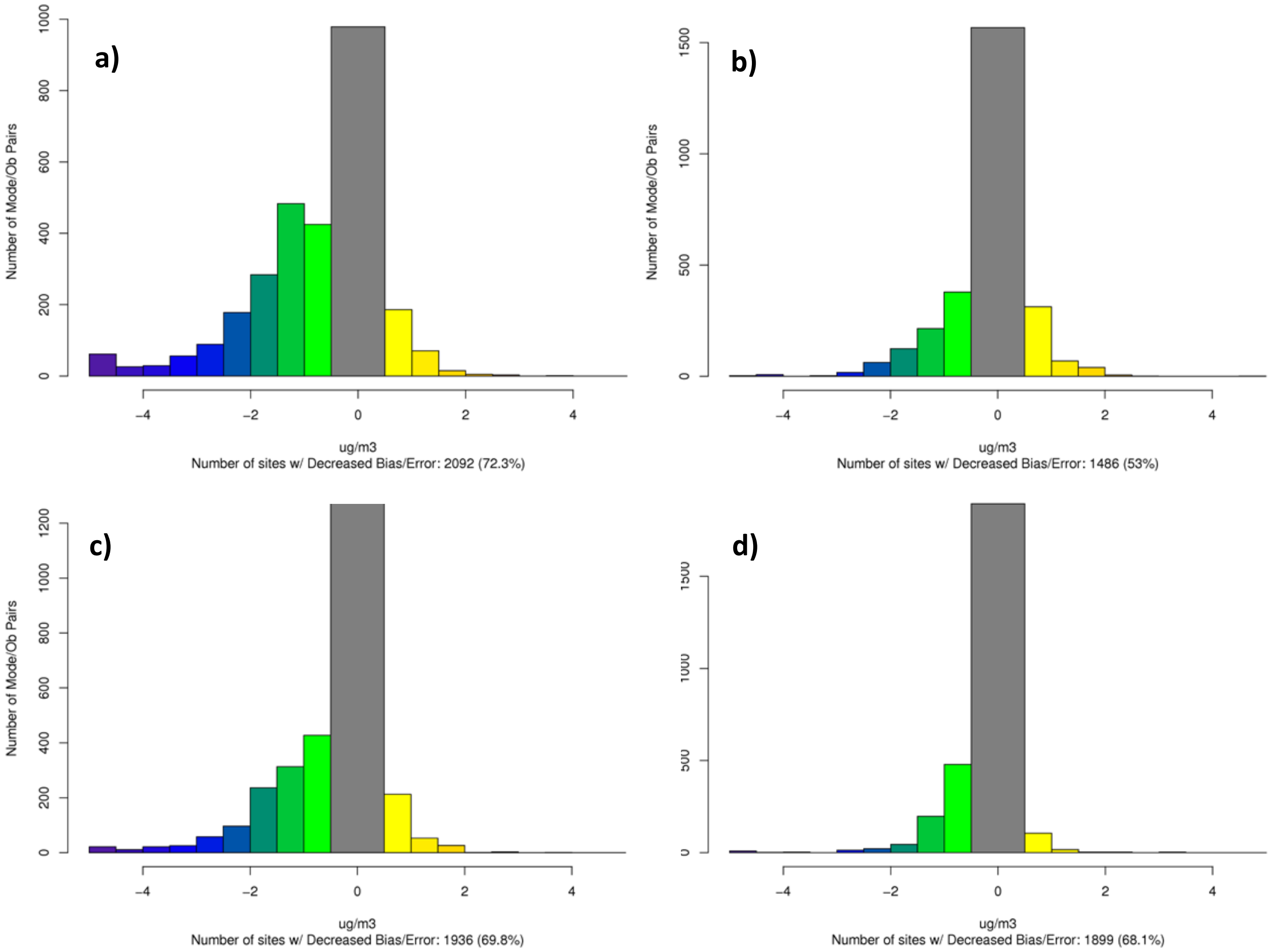

Figure S3: Histograms of the difference in the absolute value of monthly average (2011) $PM_{2.5}$ mean bias for a) winter (DJF), b) spring (MAM), c) summer (JJA) and d) fall (SON) between CMAQ v5.0.2_Base and v5.1_Base_NEIv1 (CMAQv5.1_Base _NEIv1 – CMAQv5.0.2_Base). All plots are in units of μgm^{-3}. Cool colors indicate a reduction in $PM_{2.5}$ mean bias in CMAQv5.1_Base_NEIv1 while warm color indicate an increase in $PM_{2.5}$ mean bias CMAQv5.1_Base_NEIv1.

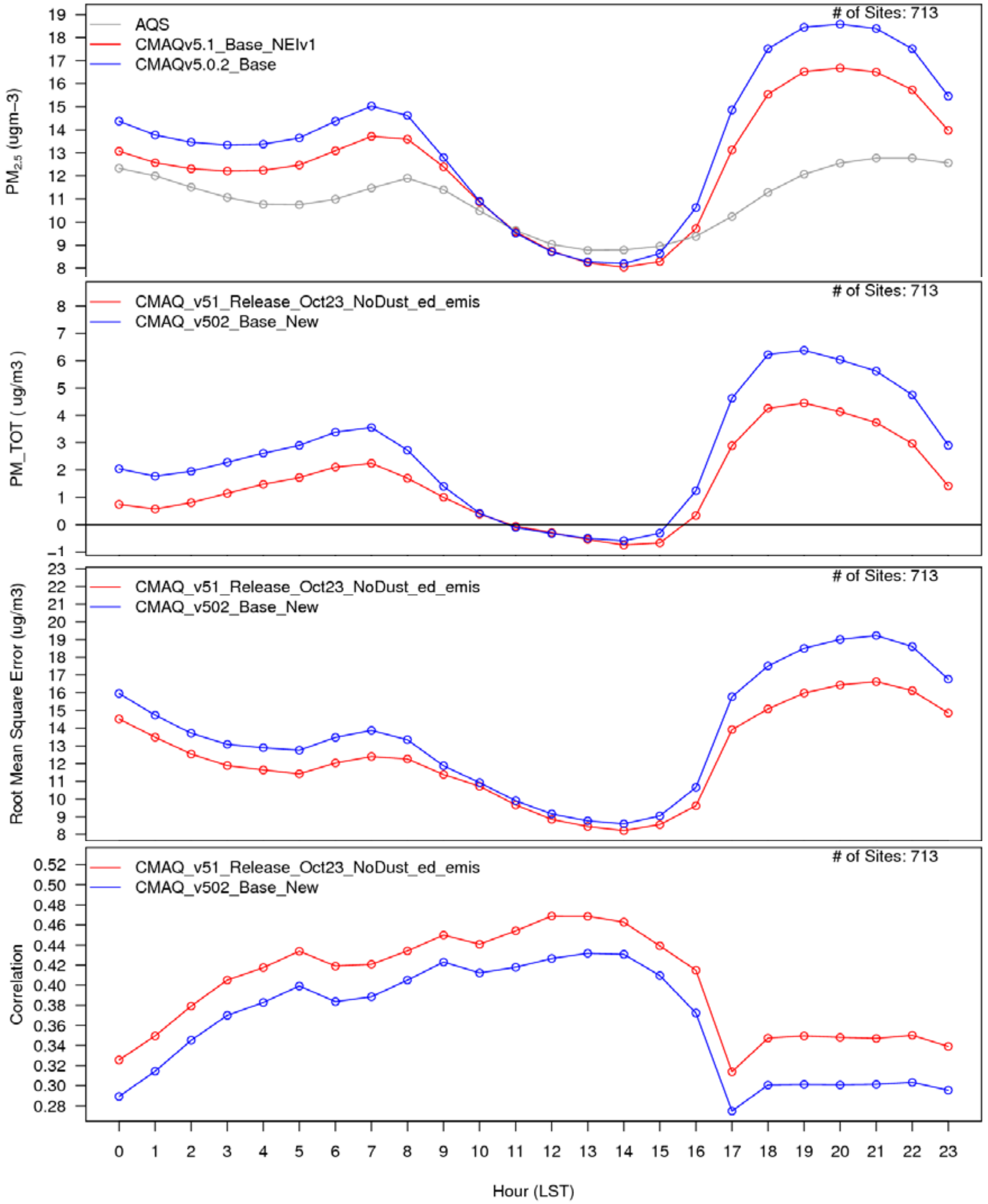

Figure S4: Diurnal time series of winter $PM_{2.5}$ from AQS observations (grey), CMAQv5.0.2_Base (blue) and CMAQv5.1_Base_NEIv1 (red) for concentration (top), mean bias (top middle), root mean square error (bottom middle) and correlation (bottom). All units are in μgm^{-3} except for correlation.

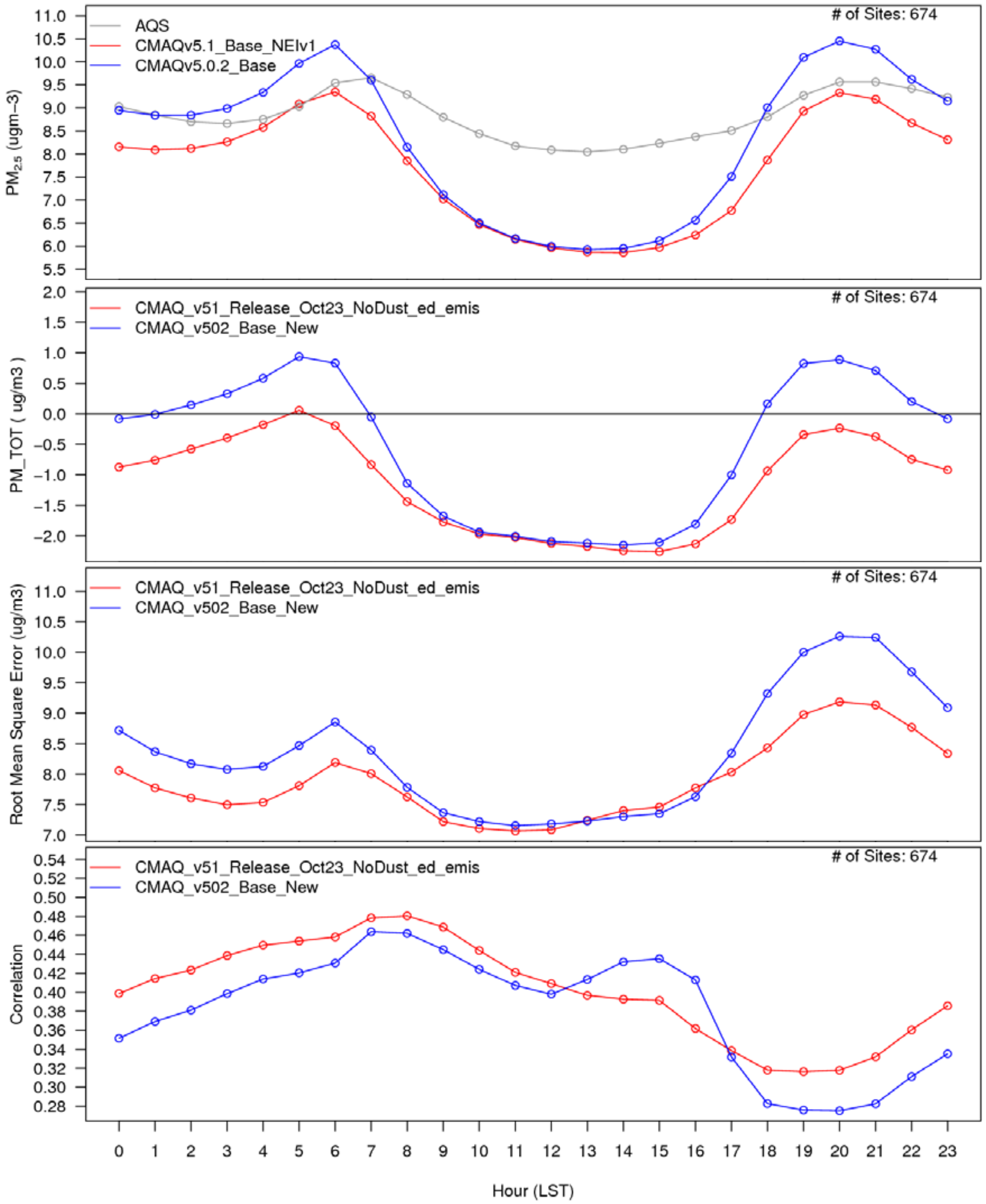

Figure S5: Diurnal time series of spring $PM_{2.5}$ from AQS observations (grey), CMAQv5.0.2_Base (blue) and CMAQv5.1_Base_NEIv1 (red) for concentration (top), mean bias (top middle), root mean square error (bottom middle) and correlation (bottom). All units are in μgm^{-3} except for correlation.

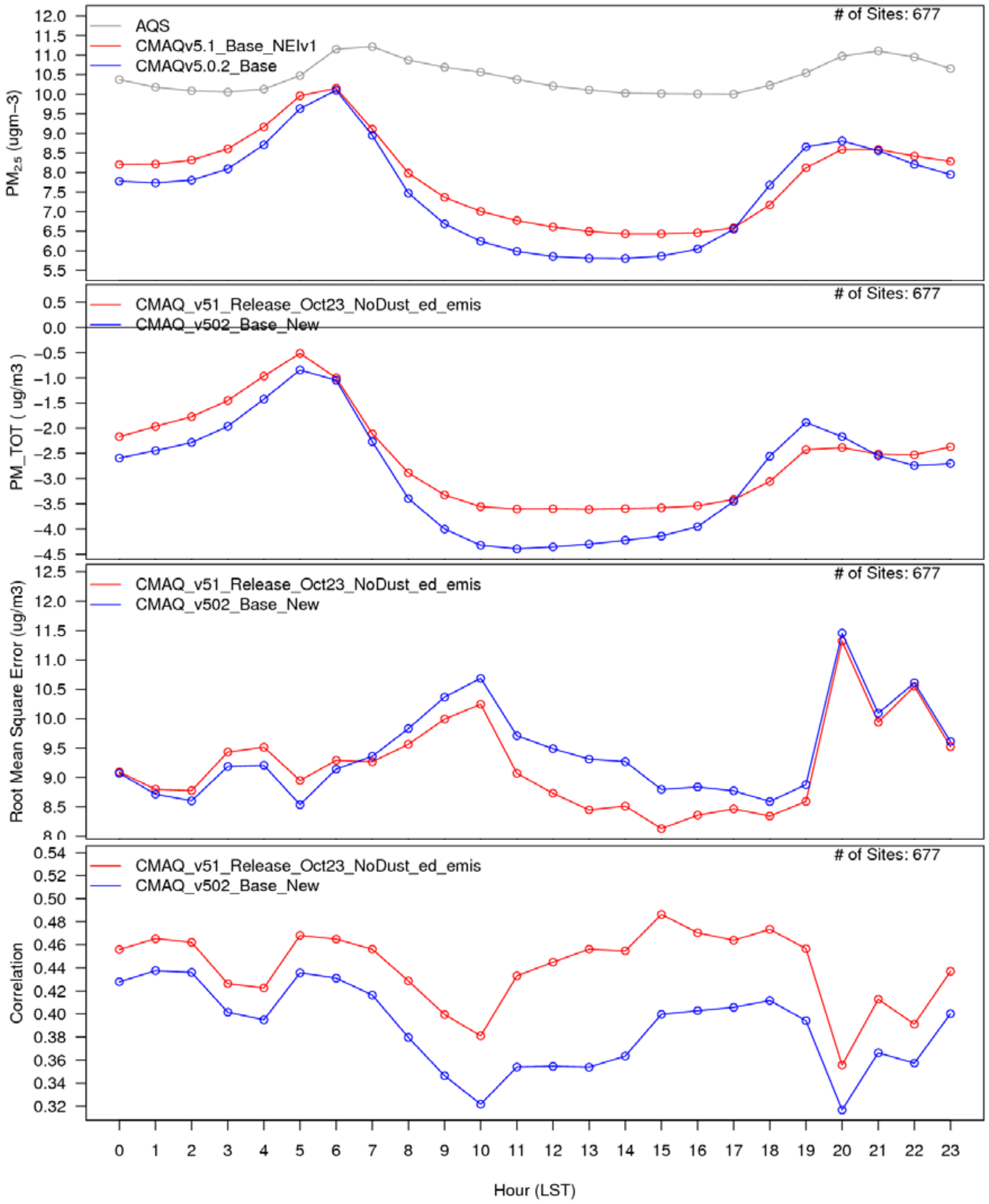

Figure S6: Diurnal time series of summer $PM_{2.5}$ from AQS observations (grey), CMAQv5.0.2_Base (blue) and CMAQv5.1_Base_NEIv1 (red) for concentration (top), mean bias (top middle), root mean square error (bottom middle) and correlation (bottom). All units are in μgm^{-3} except for correlation.

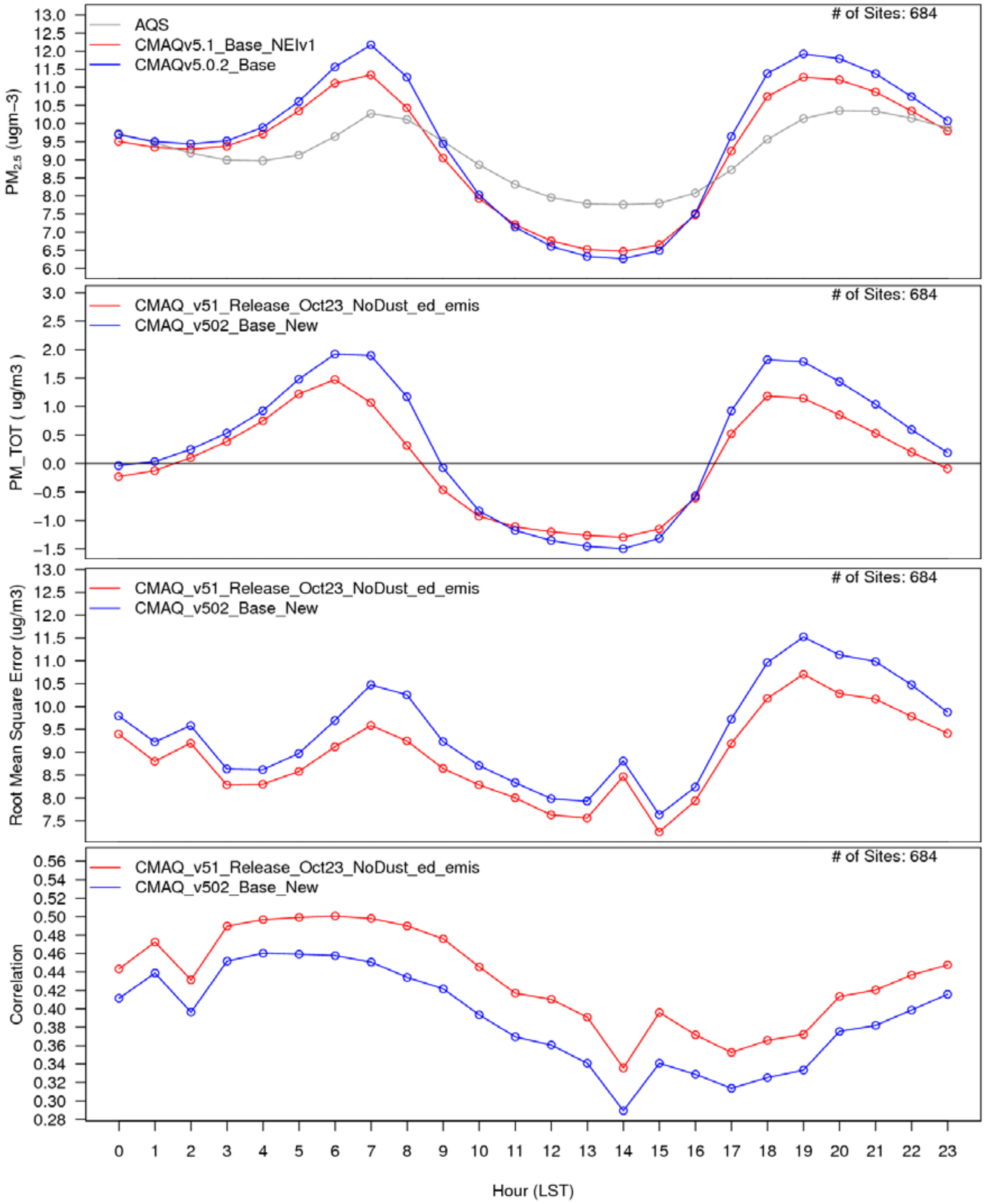

Figure S7: Diurnal time series of fall $PM_{2.5}$ from AQS observations (grey), CMAQv5.0.2_Base (blue) and CMAQv5.1_Base_NEIv1 (red) for concentration (top), mean bias (top middle), root mean square error (bottom middle) and correlation (bottom). All units are in μgm^{-3} except for correlation.

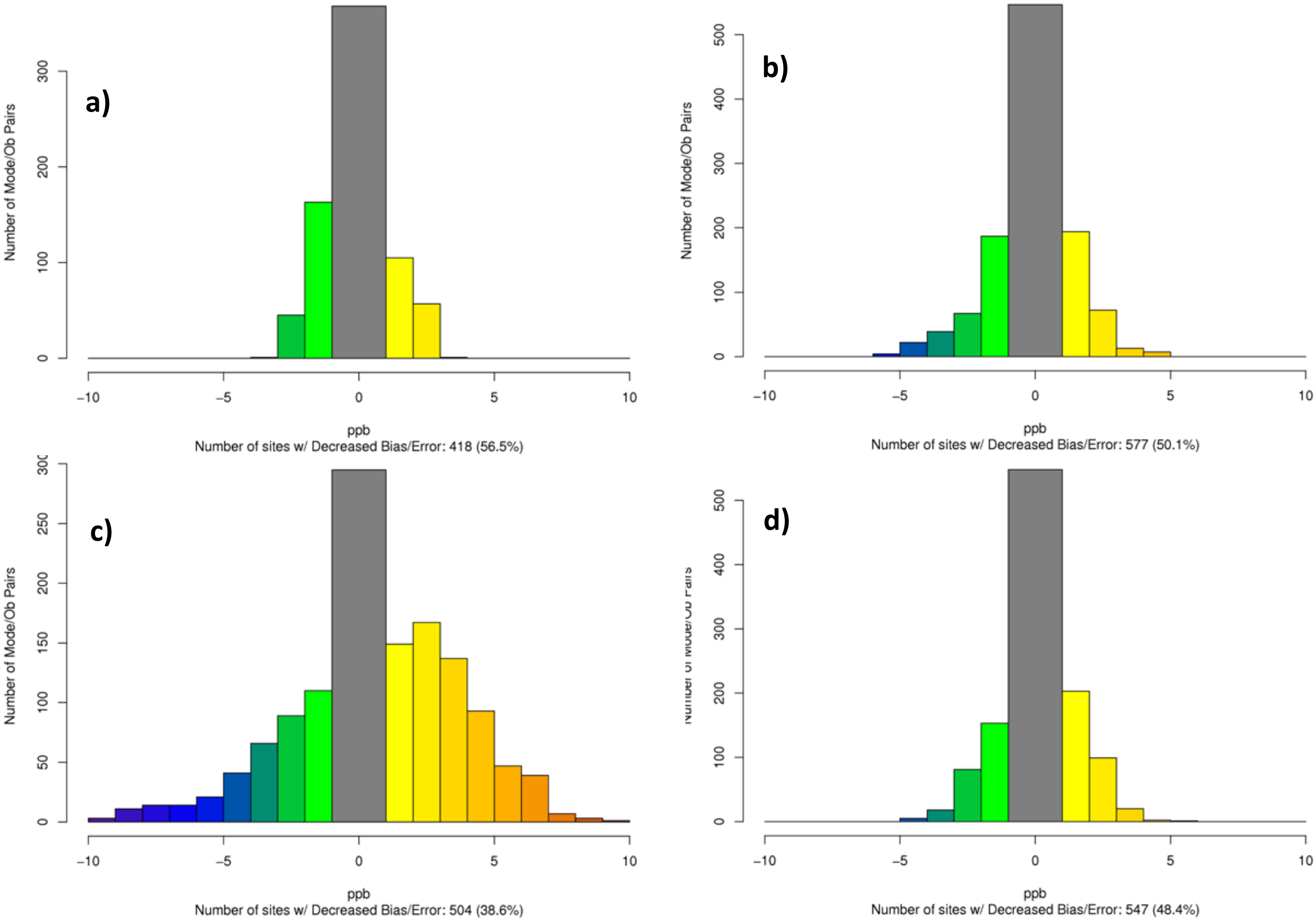

Figure S8: Histograms of the difference in the absolute value of monthly average O_3 mean bias for winter (DJF; top left), spring (MAM; top right), summer (JJA; bottom left) and fall (SON; bottom right) between CMAQ v5.0.2_Base and v5.1_Base_NEIv1 (CMAQv5.1_Base_NEIv1 – CMAQv5.0.2_Base). All plots are in units of ppbv. Cool colors indicate a reduction in O_3 mean bias in CMAQv5.1_Base_NEIv1 while warm color indicate an increase in O_3 mean bias CMAQv5.1_Base_NEIv1.

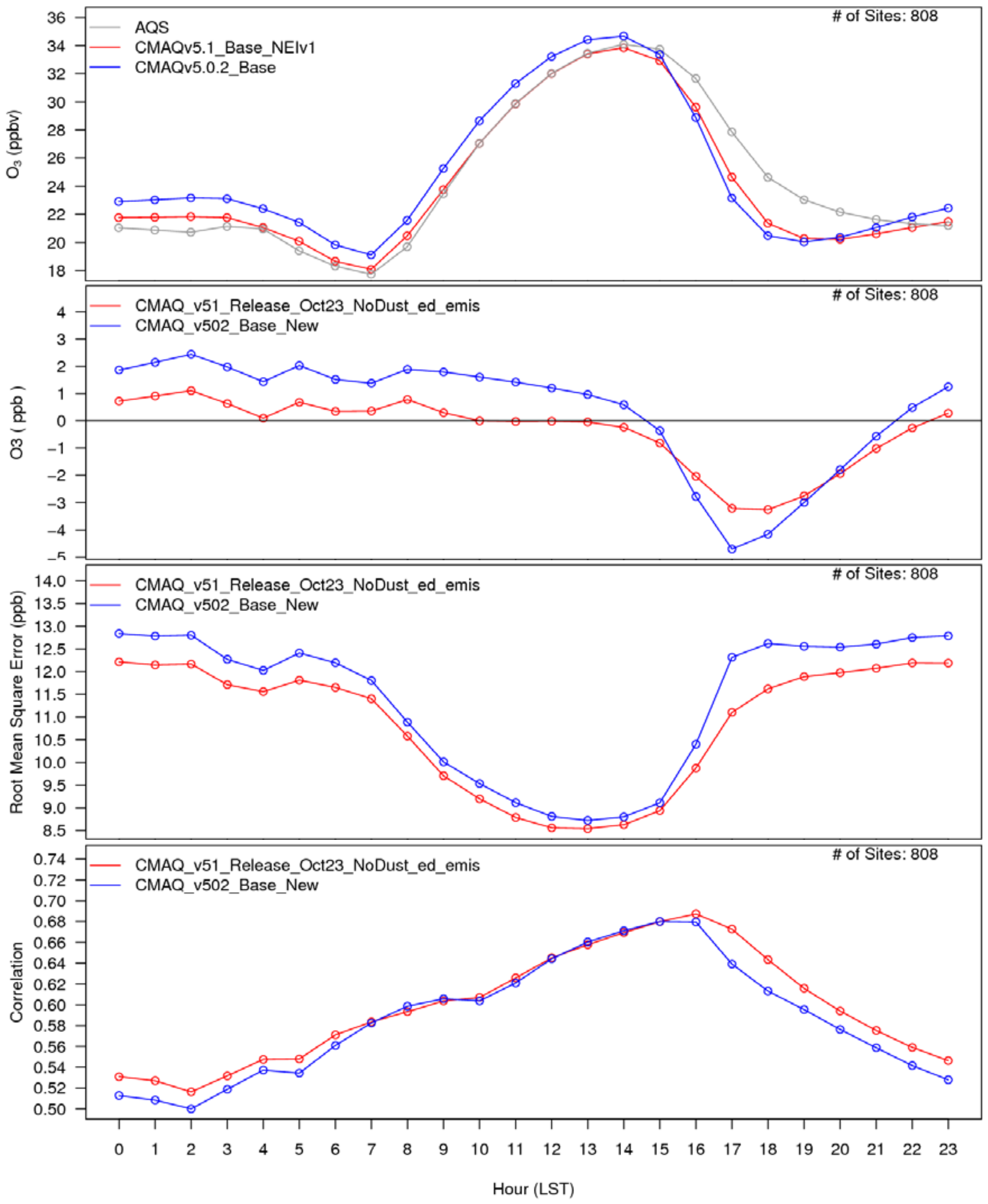

Figure S9: Diurnal time series of winter O_3 from AQS observations (grey), CMAQv5.0.2_Base (blue) and CMAQv5.1_Base_NEIv1 (red) for concentration (top), mean bias (top middle), root mean square error (bottom middle) and correlation (bottom). All units are in ppbv except for correlation.

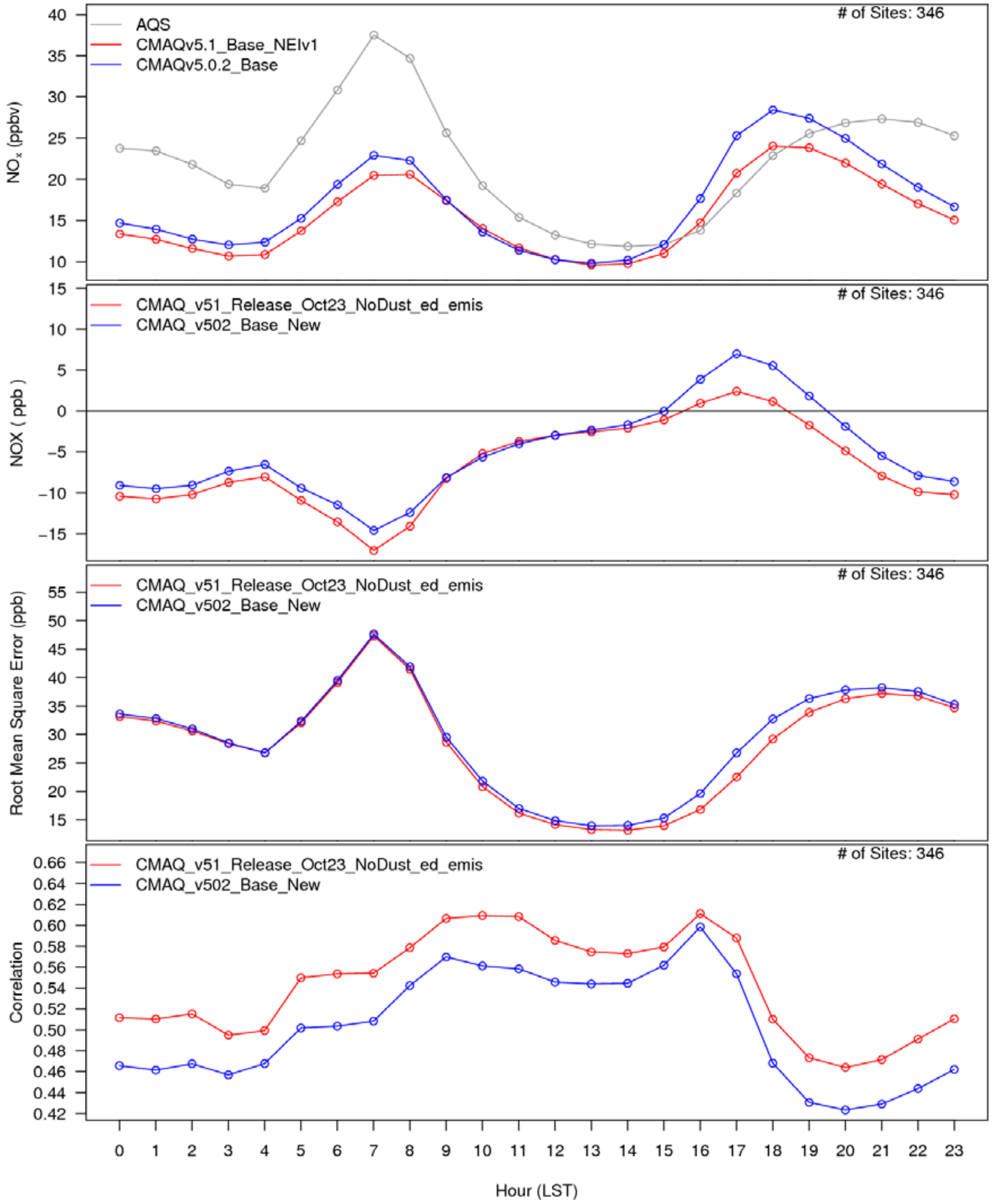

Figure S10: Diurnal time series of winter NO_x from AQS observations (grey), CMAQv5.0.2_Base (blue) and CMAQv5.1_Base_NEIv1 (red) for concentration (top), mean bias (top middle), root mean square error (bottom middle) and correlation (bottom). All units are in ppbv except for correlation.

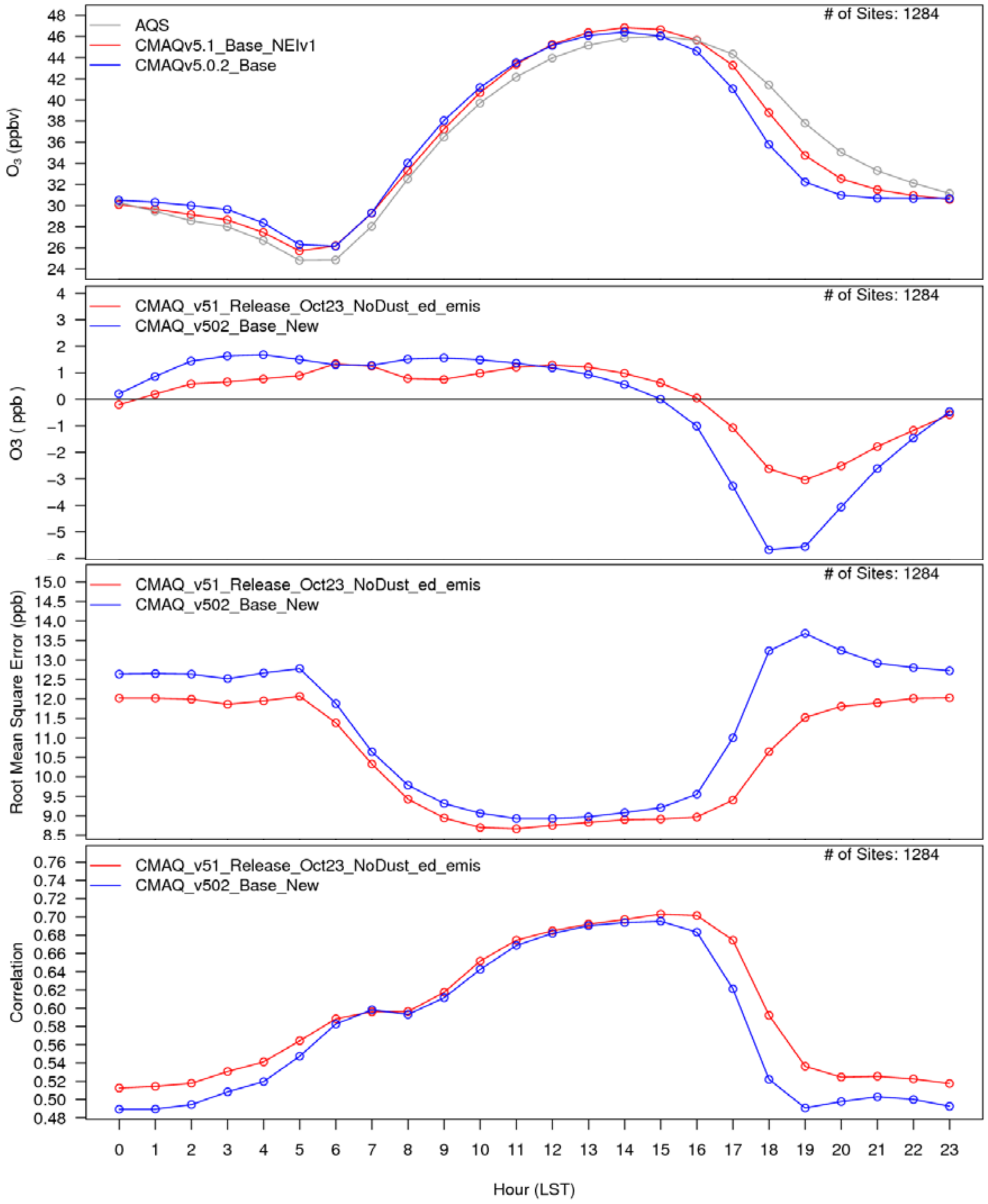

Figure S11: Diurnal time series of spring O_3 from AQS observations (grey), CMAQv5.0.2_Base (blue) and CMAQv5.1_Base_NEIv1 (red) for concentration (top), mean bias (top middle), root mean square error (bottom middle) and correlation (bottom). All units are in ppbv except for correlation.

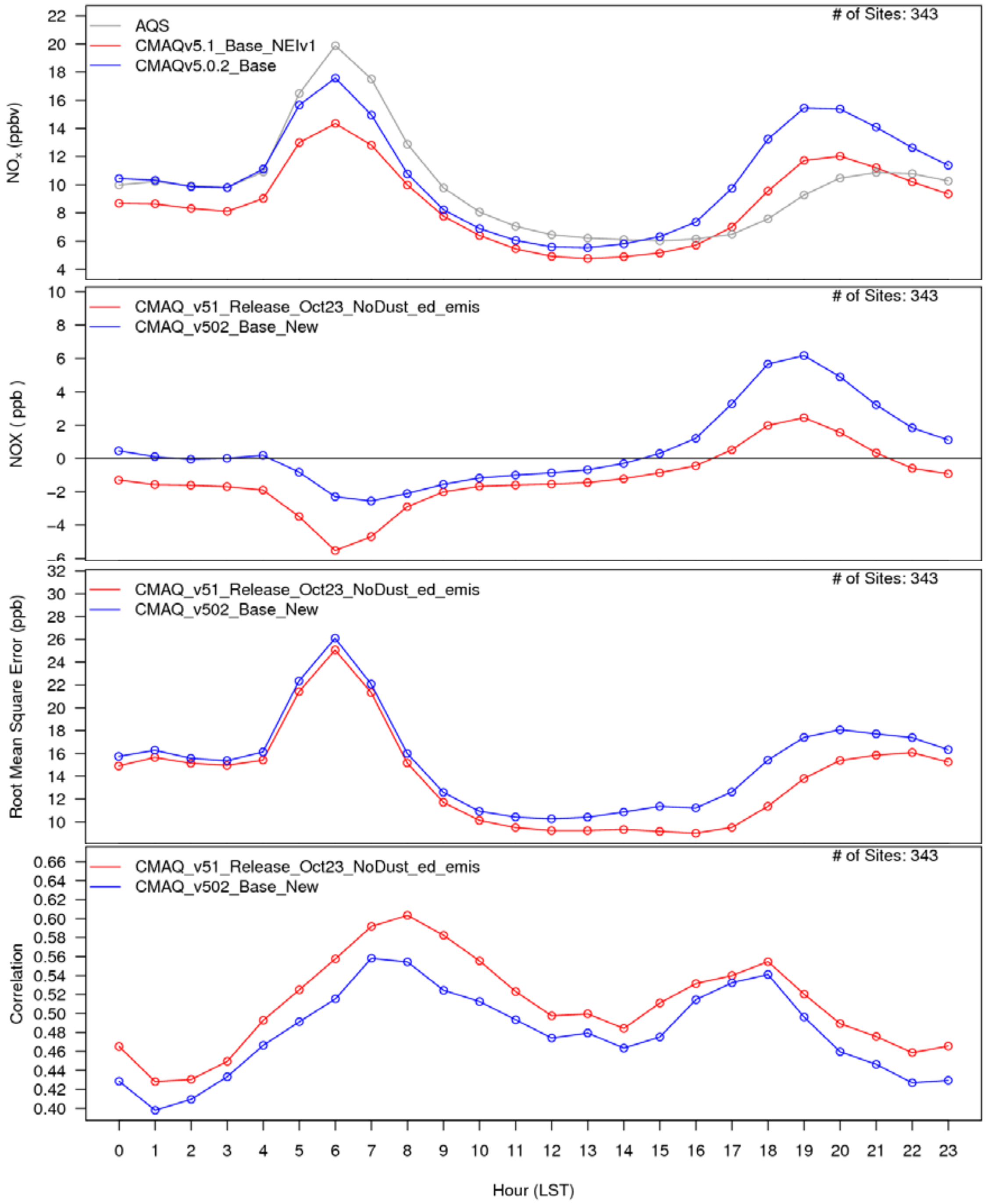

Figure S12. Diurnal time series of spring NO_x from AQS observations (grey), CMAQv5.0.2_Base (blue) and CMAQv5.1_Base_NEIv1 (red) for concentration (top), mean bias (top middle), root mean square error (bottom middle) and correlation (bottom). All units are in ppbV except for correlation.

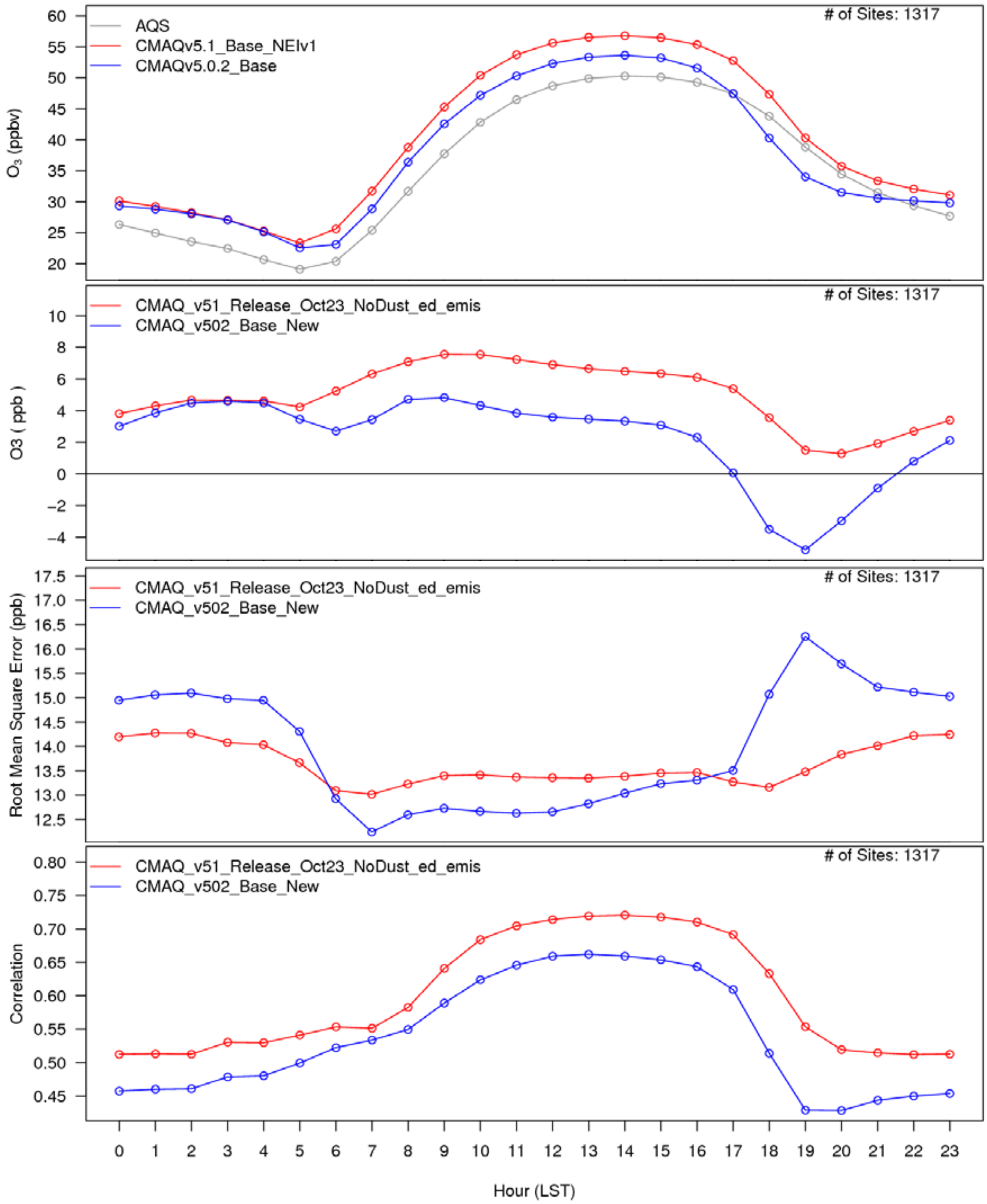

Figure S13: Diurnal time series of summer O_3 from AQS observations (grey), CMAQv5.0.2_Base (blue) and CMAQv5.1_Base_NEIv1 (red) for concentration (top), mean bias (top middle), root mean square error (bottom middle) and correlation (bottom). All units are in ppbv except for correlation.

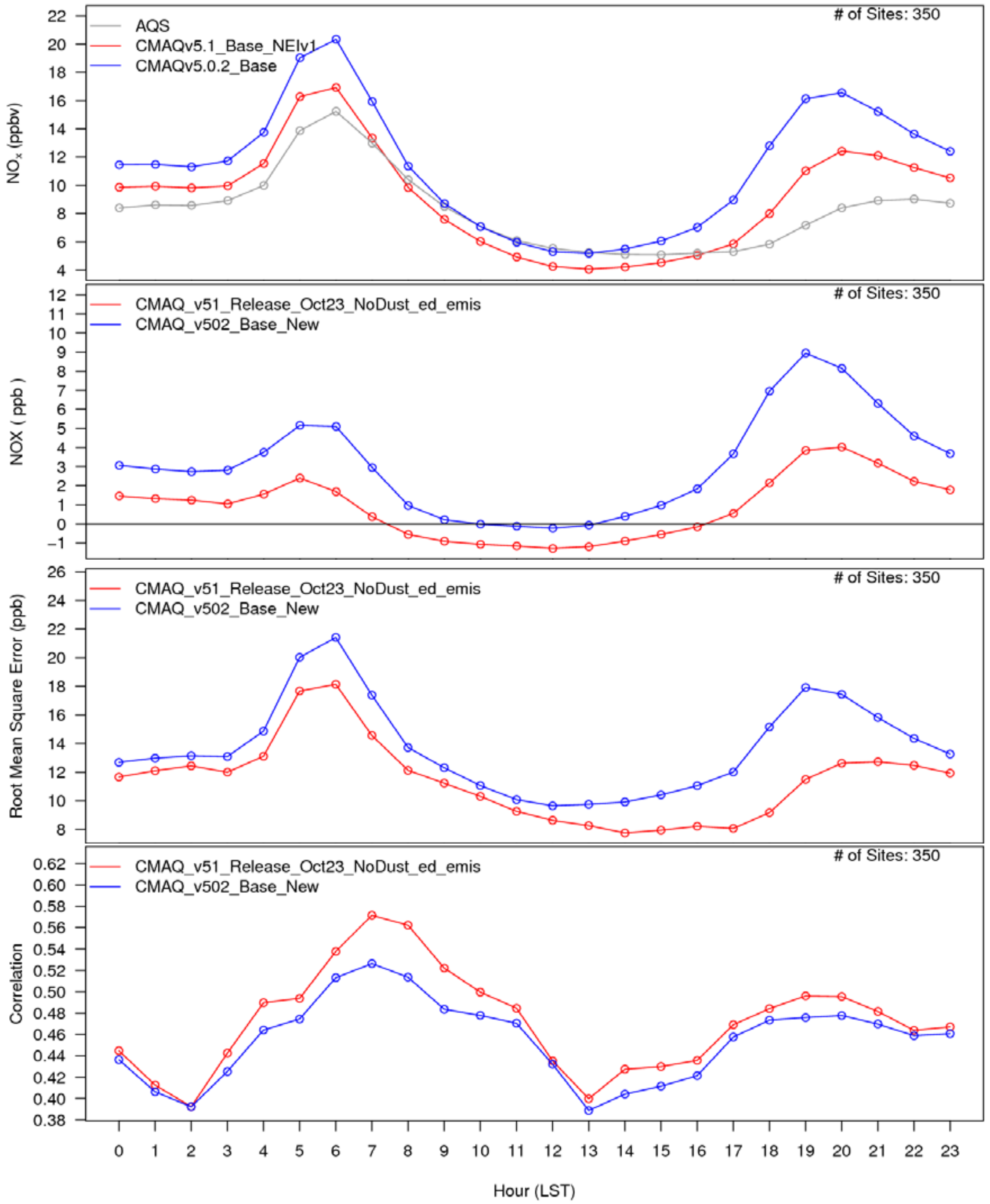

Figure S14: Diurnal time series of summer NO_x from AQS observations (grey), CMAQv5.0.2_Base (blue) and CMAQv5.1_Base_NEIv1 (red) for concentration (top), mean bias (top middle), root mean square error (bottom middle) and correlation (bottom). All units are in ppbv except for correlation.

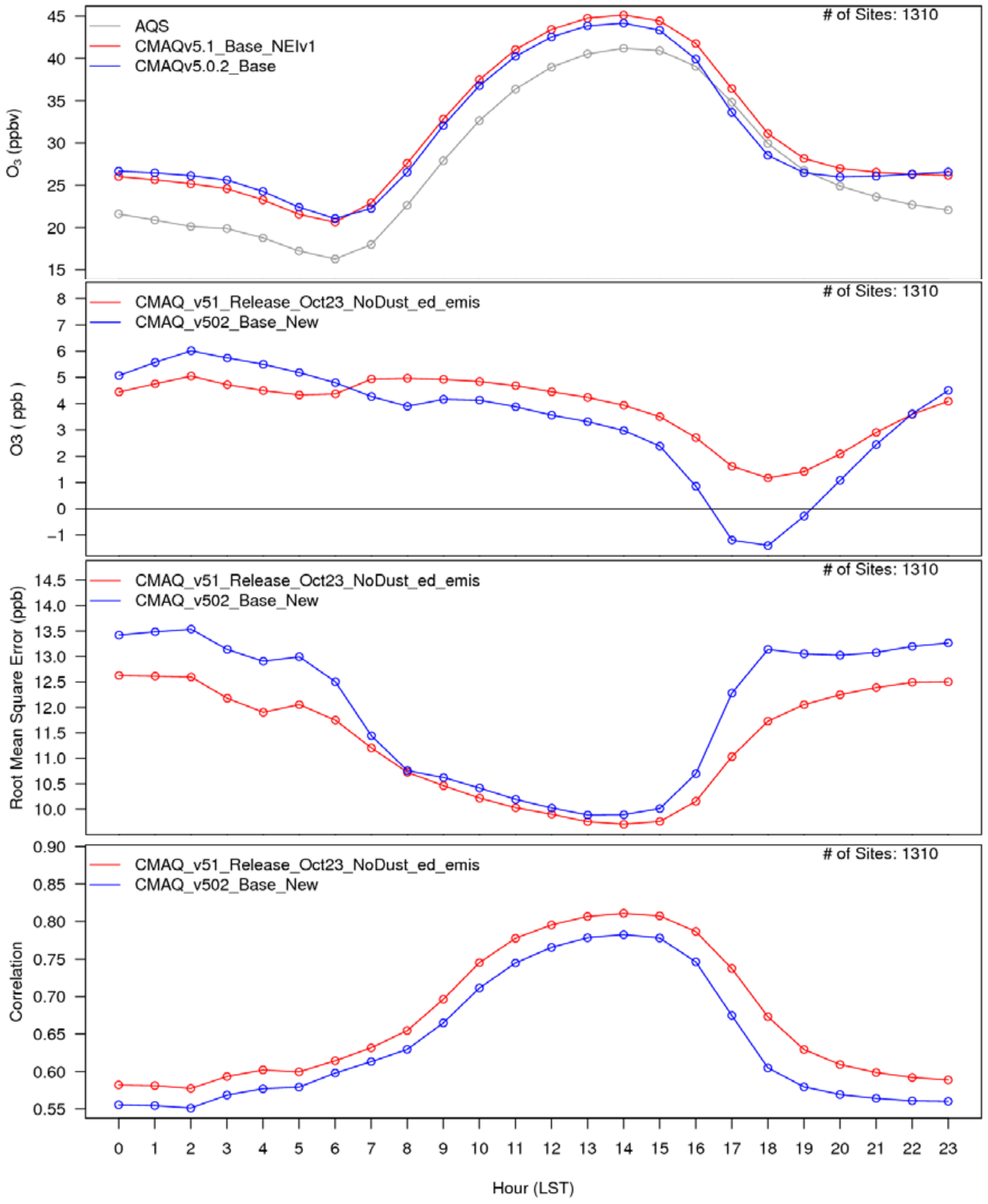

Figure S15: Diurnal time series of fall O_3 from AQS observations (grey), CMAQv5.0.2_Base (blue) and CMAQv5.1_Base_NEIv1 (red) for concentration (top), mean bias (top middle), root mean square error (bottom middle) and correlation (bottom). All units are in ppbv except for correlation.

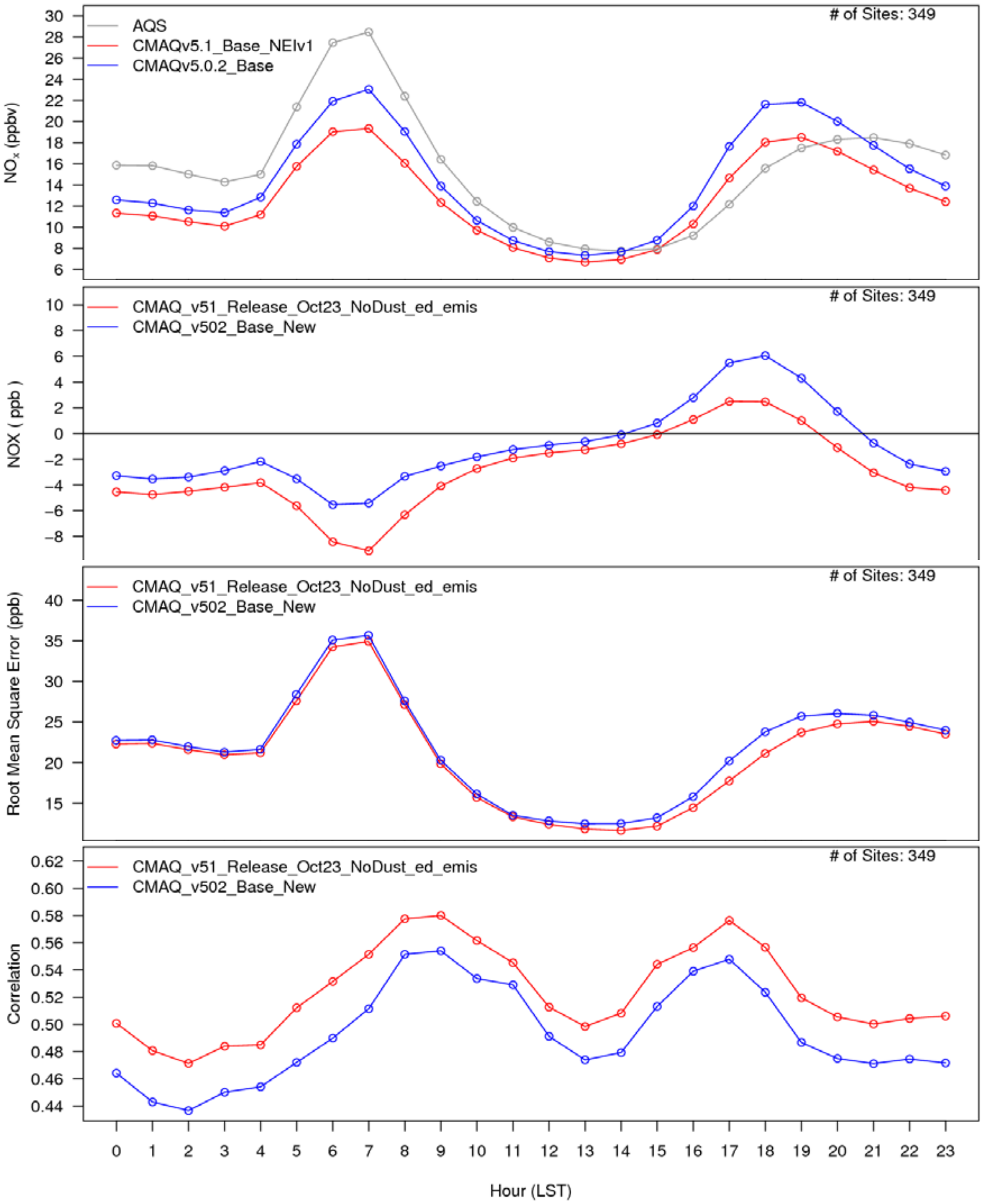

Figure S16: Diurnal time series of fall NO_x from AQS observations (grey), CMAQv5.0.2_Base (blue) and CMAQv5.1_Base_NEIv1 (red) for concentration (top), mean bias (top middle), root mean square error (bottom middle) and correlation (bottom). All units are in ppbv except for correlation.
